# Stability Studies of the Dilution Series of Different Antibiotic Stock Solutions in Culture Medium Incubated at 37 °C

**DOI:** 10.3390/antibiotics13060549

**Published:** 2024-06-12

**Authors:** Ádám Kerek, Bence G. Ecsedi, Ábel Szabó, Zoltán Szimrók, Bianka Paliczné Kustán, Ákos Jerzsele, Gábor Nagy

**Affiliations:** 1 Department of Pharmacology and Toxicology, University of Veterinary Medicine, István utca 2, H-1078 Budapest, Hungary; ecsedi.bence.gergo@student.univet.hu (B.G.E.); szabo.abel@student.univet.hu (Á.S.); szimrok.zoltan@univet.hu (Z.S.); paliczne.kustan.bianka@univet.hu (B.P.K.); jerzsele.akos@univet.hu (Á.J.); nagy.gabor@univet.hu (G.N.); 2 National Laboratory of Infectious Animal Diseases, Antimicrobial Resistance, Veterinary Public Health and Food Chain Safety, University of Veterinary Medicine, H-1078 Budapest, Hungary

**Keywords:** antibiotic, stability, dilution, culture medium, tryptone soy broth, HPLC

## Abstract

The long-term stability of antibiotics in culture media remains underexplored in scientific literature. This study evaluated the stability of eight distinct antibiotic stock solutions—amoxicillin, cefotaxime, neomycin, oxytetracycline, florfenicol, enrofloxacin, colistin, and potentiated sulfonamide—and their 10-fold dilution series in tryptone soy broth (TSB) at 37 °C, over 12 days. Samples were collected immediately after preparation and on days 1, 2, 5, 7, 9, and 12, with active substance concentrations measured using ultra-high-performance liquid chromatography (UHPLC) coupled with mass spectrometry. The results indicated that among the ultrapure water stock solutions, neomycin, florfenicol, and potentiated sulfonamide maintained stability (>95%). Within the culture medium, florfenicol showed consistent stability (100%) throughout the study, potentiated sulfonamide experienced minor degradation (>85%), and neomycin underwent significant degradation. Amoxicillin, oxytetracycline, and colistin displayed considerable degradation in both solution types but were more stable in ultrapure water solutions. The stability of cefotaxime and enrofloxacin in ultrapure water solutions and in the medium was very similar when compared; however, 3.6% of the former and 88.7% of the latter remained detectable by day 12. These findings are crucial for minimum inhibitory concentration (MIC) assessments, especially in minimum bactericidal concentration (MBC) studies, and in experiments concerning long-term evolution and co-selection. This study underscores the necessity of stability assessments in culture media to validate future experimental outcomes.

## 1. Introduction

The proliferation of antimicrobial resistance (AMR) poses one of the most significant threats to health of our time. A 2019 study highlighted that nearly five million cases of death were linked to AMR, with 1.27 million cases directly attributable to drug resistance [1]. If current trends persist, projections suggest that AMR could be responsible for up to 10 million deaths annually by 2050 [2]. This crisis is fueled not only by the irresponsible use of antibiotics, but also by their widespread dissemination [3]. At this critical juncture, testing antibiotics for antimicrobial susceptibility becomes paramount. The gold-standard methods for such assessments—the determination of minimum inhibitory concentration (MIC) and minimum bactericidal concentration (MBC)—vary in duration, lasting anywhere from 24 h to several days, depending on the organism involved. These evaluations are conducted across different culture media [4,5], with evolution and co-selection studies extending up to 10–12 days [6,7,8]. Therefore, particularly for extended-duration studies, an understanding of the stability of various antibiotics in both ultrapure water (UPW) and culture media is essential, as it can significantly influence experimental outcomes. However, stability is also a key issue for the substances that can replace antibiotics [9,10,11].

The predominant methodology for detecting and quantifying antibiotics utilizes high-pressure liquid chromatography (HPLC), often enhanced with mass spectrometry, UV, or fluorescence detection. A more advanced iteration, ultra-high-pressure liquid chromatography (UHPLC), offers further improvements, including increased accuracy and reduced analysis time [12]. Additionally, the emerging technique of nanofluid chromatography is coming to the fore, characterized by its heightened sensitivity in molecular mass determination [13]. These advances underscore the ongoing refinement in analytical methods, ensuring more precise and efficient antibiotic quantification.

Understanding the stability of antibiotics is crucial for their study, yet the literature on their long-term stability in food and feed is sparse. Research typically focuses on short-term stability in UPW media. Among these studies, amoxicillin has garnered the most attention, with its instability primarily attributed to hydrolysis, influenced by temperature, pH [14], and solution concentration [15]. Experimental data indicate that amoxicillin solutions maintain over 90% stability for 1–3 days at 20–25 °C [16,17], with a notable reduction at 37 °C [17,18]. Similarly, cefotaxime’s stability is compromised by hydrolysis, affected by temperature [19,20], and diminishes with high concentrations of macromolecules [21]. Various studies have shown that at room temperature, UPW solutions of cefotaxime exhibit stability for only a few hours [20,22,23]. Colistin’s stability in UPW solutions is susceptible to oxidation, reduction, hydrolysis, β-elimination, and racemization [24], with experiments suggesting pH has a more significant impact than temperature. In acidic conditions, colistin’s half-life remains extensive even at elevated temperatures, but it decreases notably under basic conditions [25,26]. Oxytetracycline’s stability is compromised by factors such as oxidation, hydrolysis, pH, temperature, and light exposure [27]. Data on the stability of oxytetracycline are notably scarce, presenting a challenge for comprehensive understanding. At −20 °C, the stability outcomes reported by various studies are markedly inconsistent [28,29,30]. German et al. found the solution to be stable for one week at 2–8 °C [28]. However, a significant finding from another study was the rapid degradation of oxytetracycline at 37 °C, where its half-life was determined to be only 34 h [31].

Literature reviews reveal that UPW solutions of enrofloxacin exhibit remarkable stability, maintaining their integrity at room temperature across various experiments, even in the presence of additional molecules, such as sweeteners and dexamethasone [32,33,34]. Similarly, the stability of florfenicol has predominantly been assessed under severe conditions, demonstrating robustness at elevated temperatures [35,36]. Conversely, data on the stability of potential sulfonamide (a sulfamethoxazole–trimethoprim combination) are scarce; however, a synthesis of available studies suggests that the stability of its UPW solutions is significantly influenced by the concentration of the active ingredient [37]. Remarkably, there is a notable gap in the literature regarding the stability of neomycin.

This study aims to elucidate the stability of various antibiotics critical to veterinary and public health in UPW solutions and their decimal dilution series in TSB at 37 °C, over an incubation period of 12 days. Our investigation seeks to address the shortfalls identified in previous studies, providing comprehensive insights into the behavior of these antibiotics, thereby contributing valuable data to the field of antimicrobial resistance research.

## 2. Results

The quality control methods for the stability measurements of various compounds were meticulously evaluated to ensure precise and reliable analytical results. Across all tested substances—amoxicillin, cefotaxime, oxytetracycline, florfenicol, enrofloxacin, colistin, neomycin, trimethoprim, and sulfamethoxazole—the percentages of relative standard deviation (RSD) were consistently low, indicating high measurement precision (Supplementary Tables S1, S6, S11, S16, S21, S26, S31, S36 and S41). Calibration curves demonstrated extremely high correlation coefficients (r) and coefficients of determination (r^2^), underscoring the accuracy and reliability of the data (Supplementary Tables S2, S7, S12, S17, S22, S27, S32, S37 and S42).

The analytical processes exhibited minimal standard deviation among replicates, further attesting to their stability and repeatability (Supplementary Tables S3, S8, S13, S18, S23, S28, S33, S38 and S43). The methods also showed low limits of detection (LOD) and quantification (LOQ), highlighting their sensitivity and accuracy, although significant inter-iteration variance (CV%) indicated substantial variability in background noise, potentially affecting determination accuracy at very low concentrations (Supplementary Tables S4, S9, S14, S19, S24, S29, S34, S39 and S44).

System suitability tests confirmed the proper operation and appropriateness of the chromatographic systems for analysis, showing consistent and reliable performance across the tested concentration ranges (Supplementary Tables S5, S10, S15, S20, S25, S30, S35, S40 and S45). Chromatographic analysis revealed optimal peak separation with symmetrical peak shapes and without significant adjacent peaks, ensuring clear separation from other components for precise quantification (Supplementary Figures S1–S9). The overlay chromatograms are displayed in Supplementary Figures S10–S18. In the COL overlay chromatogram, the second peak pair corresponds to Polymyxin E-1, while we examined Polymyxin E-2 (more sensitive, constant ratio due to the standard). In the enrofloxacin chromatogram, significantly more transition chromatograms are visible because the method was originally developed for the simultaneous determination of levofloxacin, enrofloxacin, ciprofloxacin, and moxifloxacin. In some cases, the last decimal place of the retention time in the raw chromatogram differs by one hundredth from that in the evaluated chromatogram. This discrepancy occurs because the MultiQuant software (v3.0.3) uses Gaussian Smoothing during evaluation, and—as with other smoothing methods—the peak may shift slightly in the smoothed data set.

**Figure 1:**
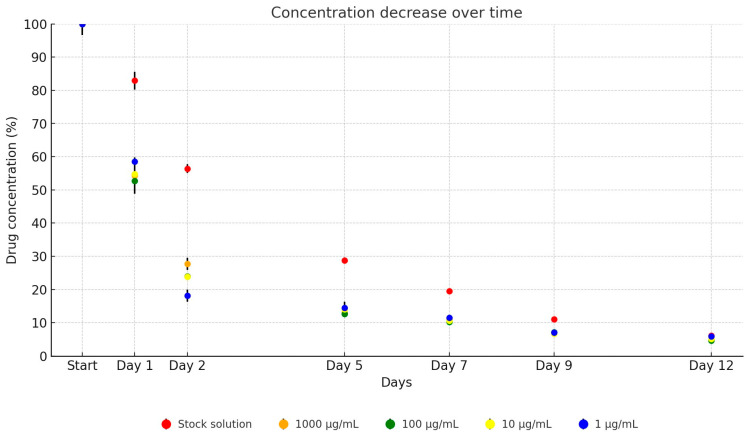
Change in active substance concentration of amoxicillin stock solution and its dilution in broth during incubation at 37 °C.

In our investigation, UPW solutions of amoxicillin demonstrated significantly enhanced stability compared to those diluted in tryptone soy broth (TSB), exhibiting nearly double the stability. A consistent trend of concentration decline was observed across all dilutions over the study period. On the first day, the average concentration of amoxicillin was 55.1% of the initial concentration, decreasing to 23.4% by the second day, 14.5% by the fifth day, 10.9% by the seventh day, 7% by the ninth day, and finally, to 5.1% by the twelfth day, as depicted in Figure 1. Over the 12-day period, the antibiotic active ingredient in the stock solution decreased to 6.1% of its original concentration (from 4728.42 µg/mL to 288.49 µg/mL). The dilutions showed similar decreases: to 4.8% (from 985.95 µg/mL to 46.85 µg/mL), to 4.8% (from 96.41 µg/mL to 4.67 µg/mL), to 5.0% (from 11.25 µg/mL to 0.56 µg/mL), and to 6.1% (from 1.15 µg/mL to 0.07 µg/mL).

**Figure 2:**
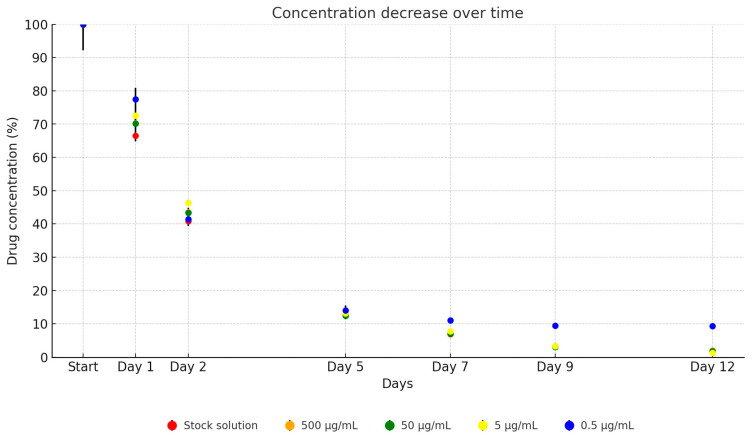
Change in active substance concentration of cefotaxime stock solution and its dilution in broth during incubation at 37 °C.

In our study, no significant difference was observed in the stability of cefotaxime between its UPW solution and the TSB dilution series. For the dilutions, the detectable drug concentration after one day averaged 72.7% of the initial concentration, dropping to 43.7% by the second day, 13.1% by the fifth day, 8.2% by the seventh day, 4.8% by the ninth day, and finally to 3.6% by the twelfth day, as illustrated in Figure 2. Over the 12-day period, the concentration of the antibiotic active ingredient in the stock solution decreased to 2.0% of its original level (from 4762.10 µg/mL to 95.05 µg/mL). The dilutions showed similar reductions: to 2.0% (from 511.97 µg/mL to 10.25 µg/mL), to 1.8% (from 54.59 µg/mL to 0.98 µg/mL), to 1.4% (from 5.6 µg/mL to 0.8 µg/mL), and to 10.3% (from 0.58 µg/mL to 0.06 µg/mL).

**Figure 3:**
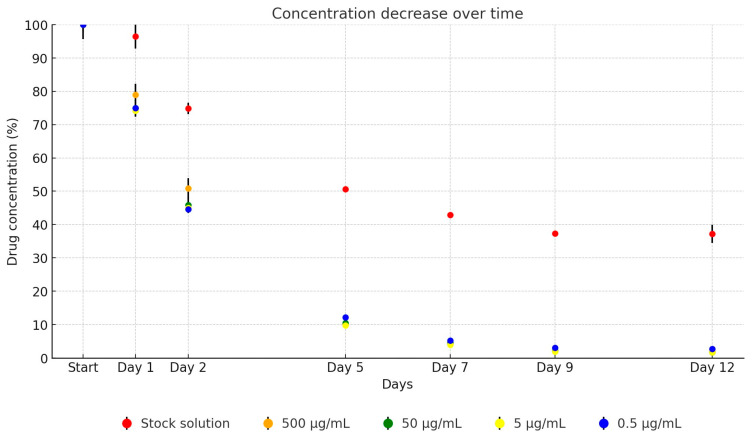
Change in active substance concentration of oxytetracycline stock solution and its dilution in broth during incubation at 37 °C.

Similar to the amoxicillin, the oxytetracycline stock solution exhibited significantly greater stability in UPW media compared to its dilutions in TSB. The dilution series retained, on average, 75.8% of its initial concentration on day 1, decreasing to 46.6% by day 2, 10.6% by day 5, 4.6% by day 7, 2.3% by day 9, and finally to 2% by day 12, as depicted in Figure 3. Over the 12-day period, the concentration of the antibiotic active ingredient in the stock solution decreased to 37.7% of its original level (from 5053.63 µg/mL to 1903.01 µg/mL). The dilutions showed the following reductions: to 2.0% (from 488.37 µg/mL to 9.59 µg/mL), to 1.7% (from 51.04 µg/mL to 0.86 µg/mL), to 1.8% (from 5.15 µg/mL to 0.09 µg/mL), and to 1.9% (from 0.54 µg/mL to 0.01 µg/mL).

**Figure 4:**
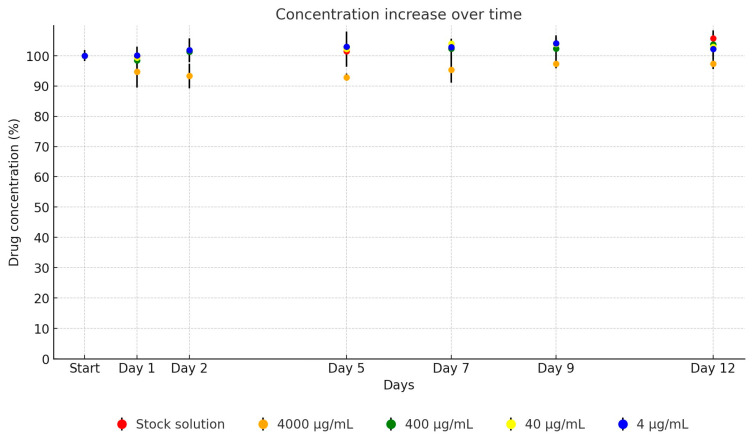
Change in active substance concentration of florfenicol stock solution and its dilution in broth during incubation at 37 °C.

Significantly, the florfenicol UPW solution and its TSB dilution series exhibited remarkable stability across the entire testing period. Near-complete stability, approaching 100% relative to the initial concentration, was consistently observed, as detailed in Figure 4. Over the 12-day period, no significant decrease in the concentration of the antibiotic active ingredient in the stock solution was observed. The measured results were within the margin of error, showing 105.8% (from 19,468.11 µg/mL to 20,594.18 µg/mL). The dilutions were measured as follows: 97.0% (from 3916.59 µg/mL to 3798 µg/mL), 105.8% (from 444.09 µg/mL to 469.64 µg/mL), 105.1% (from 44.03 µg/mL to 46.26 µg/mL), and 103.4% (from 5.25 µg/mL to 5.43 µg/mL).

**Figure 5:**
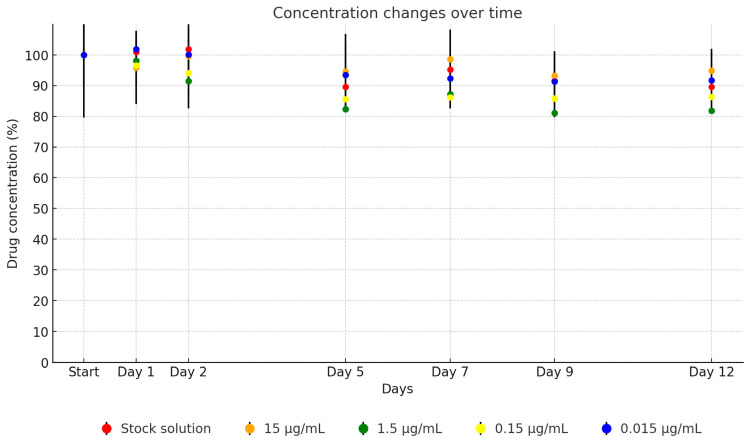
Change in active substance concentration of enrofloxacin stock solution and its dilution in broth during incubation at 37 °C.

The UPW solution of enrofloxacin, including its stock solution, displayed notable stability early in the testing period. On day 2, the stock solution remained highly stable, with the average concentration of the dilutions reducing very slightly to 98.1% on day 1 and slightly further to 96.3% on day 2. The slow decline continued, with an average of 88.7% of the enrofloxacin still detectable by day 12, as shown in Figure 5. Over the 12-day period, the concentration of the antibiotic active ingredient in the stock solution decreased to 88.2% of its original level (from 156.39 µg/mL to 138.0 µg/mL). The dilutions showed the following reductions: to 95.9% (from 16.77 µg/mL to 16.08 µg/mL), to 81.2% (from 2.07 µg/mL to 1.68 µg/mL), to 82.6% (from 0.23 µg/mL to 0.19 µg/mL), and to 95.0% (from 0.02 µg/mL to 0.019 µg/mL).

**Figure 6:**
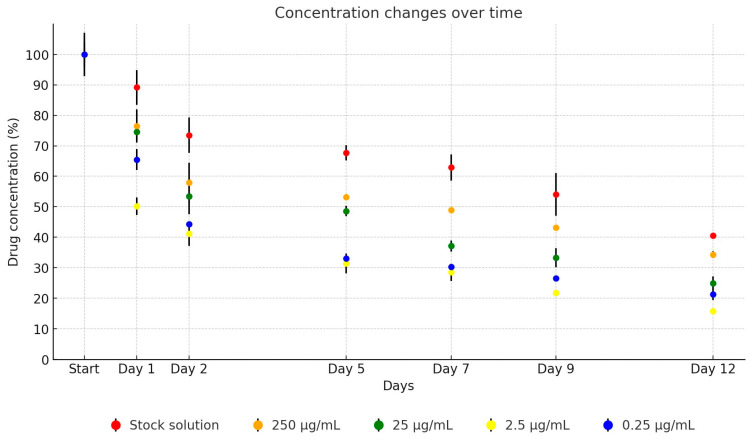
Change in active substance concentration of colistin stock solution and its dilution in broth during incubation at 37 °C.

For the UPW solution of colistin, a marked reduction in concentration was evident within the first 24 h of incubation, with a steady decrease observed over the remaining test period. By day 12, the average remaining concentration of the active substance had diminished to 40.5% of the initial level. When diluted in broth, the active substance demonstrated a more pronounced decline, with only 66.7% of the initial concentration detectable after one day, which dropped to an average of 24.1% by day 12, as illustrated in Figure 6. Over the 12-day period, the concentration of the antibiotic active ingredient in the stock solution decreased to 39.3% of its original level (from 2918.08 µg/mL to 1146.45 µg/mL). The dilutions showed the following reductions: to 34.1% (from 242.96 µg/mL to 82.78 µg/mL), to 24.5% (from 27.55 µg/mL to 6.75 µg/mL), to 16.3% (from 3.01 µg/mL to 0.49 µg/mL), and to 20.5% (from 0.39 µg/mL to 0.08 µg/mL).

**Figure 7:**
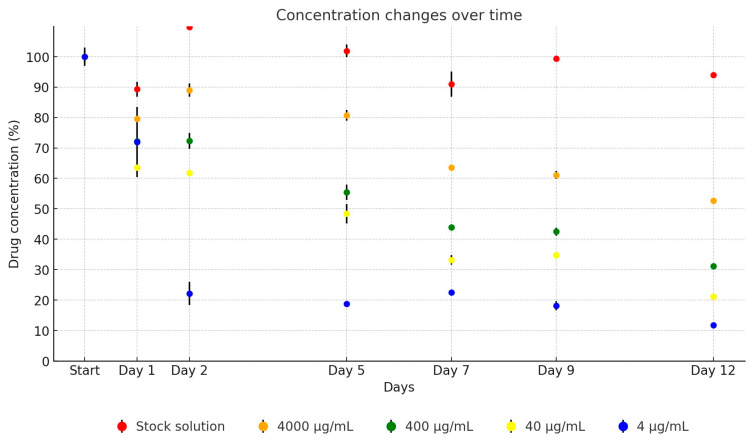
Change in active substance concentration of neomycin stock solution and its dilution in broth during incubation at 37 °C.

The neomycin stock solution exhibited relative stability throughout the study. However, when averaged across the dilutions in TSB, a notable degradation of the active substance was observed over time. After just one day of incubation, only 71.8% of the active ingredient remained on average, with a consistent average decrease of 10% per subsequent measurement. By the conclusion of the testing period, the remaining concentration of the original drug averaged at 29.2%, as demonstrated in Figure 7. Over the 12-day period, the concentration of the antibiotic active ingredient in the stock solution decreased to 93.3% of its original level (from 43,472.35 µg/mL to 40,552.25 µg/mL). The dilutions showed the following reductions: to 52.6% (from 4354.81 µg/mL to 2289.46 µg/mL), to 31.1% (from 409.3 µg/mL to 127.36 µg/mL), to 21.7% (from 35.69 µg/mL to 7.75 µg/mL), and to 11.8% (from 2.2 µg/mL to 0.26 µg/mL).

For potential sulfonamide, the stability dynamics of trimethoprim and sulfamethoxazole differed notably between UPW solutions and TSB dilutions. The stability of the UPW solution of trimethoprim remained largely unchanged until day 12, at which point a slight decrease to 96.5% was observed. In contrast, the average concentration of trimethoprim in TSB began to decline by day 5, dropping to 97.1%, and decreasing further to 91.1% by the end of the study. Throughout the 12-day observation period, the antibiotic active ingredient in the stock solution reduced to 96.2% of its initial concentration (from 910.93 µg/mL to 876.1 µg/mL). The dilutions showed the following reductions: 90.2% (from 99.42 µg/mL to 89.66 µg/mL), 92.9% (from 10.93 µg/mL to 10.15 µg/mL), 92.7% (from 1.23 µg/mL to 1.14 µg/mL), and 83.3% (from 0.12 µg/mL to 0.1 µg/mL).

**Figure 8:**
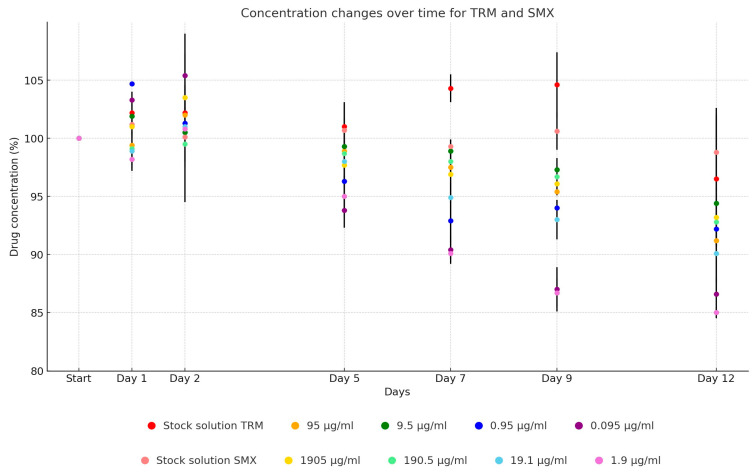
Change in active substance concentration of potential sulfonamide stock solution and its dilution in broth during incubation at 37 °C.

Sulfamethoxazole exhibited a similar pattern, with its UPW solution maintaining stability until a marginal reduction to 98.8% was noted on day 12. The TSB dilutions mirrored the trend observed with trimethoprim, starting to decline on day 5 (97.4%) and averaging at 90.3% of the initial concentration by day 12, as depicted in Figure 8. Over the span of 12 days, the antibiotic active ingredient concentration in the stock solution decreased to 99.2% of its initial level (from 19,573.0 µg/mL to 19,407.56 µg/mL). The dilutions showed reductions as follows: 94.6% (from 2002.74 µg/mL to 1895.02 µg/mL), 9.5% (from 201.86 µg/mL to 190.82 µg/mL), 89.8% (from 21.13 µg/mL to 18.98 µg/mL), and 81.6% (from 2.56 µg/mL to 2.09 µg/mL).

Supplementary Tables S49 and S50 summarize the concentration values for each active substance on the measurement days, expressed as a percentage of the initial baseline concentration. Table 1 provides a comprehensive summary of the equations and explanatory power of logarithmic trend lines applied to the mean dilution series for each active substance. It also evaluates the origin of each sample from the same distribution through a non-parametric statistical method. In instances where significance is observed (*p* < 0.05), it indicates that at least one sample exhibits stochastic dominance over another, highlighting variability in the stability or degradation patterns among the samples.

## 3. Discussion

We assessed the stability of stock solutions and TSB dilutions of eight antibiotics (amoxicillin, cefotaxime, neomycin, oxytetracycline, florfenicol, enrofloxacin, colistin, and sulfamethoxazole–trimethoprim) at 37 °C, over a 12-day incubation period. Our findings underscore the critical nature of such assays for ongoing antimicrobial resistance surveillance and the need for timely interventions. Additionally, the results emphasize the imperative to curtail antibiotic usage and, where feasible, to seek alternative treatments [11,38,39,40,41].

Among the antibiotics analyzed, notable for both veterinary and public health, amoxicillin exhibited the greatest stability in its UPW stock solution compared to its diluted form in TSB. One day post-incubation, the stock solution retained 82.9% of its initial concentration, whereas the dilution averaged at 55.1% retention. By the 12th day, only approximately 5% of the active substance was detectable in both mediums. While previous research has indicated that UPW amoxicillin solutions remain stable at −20 °C for up to three months [29,30], Lugoboni et al. reported stability up to 20 days before a notable concentration decline [14]. Vahdat et al. explored amoxicillin solutions at varying freezing temperatures and in buffer solutions, noting a stability exceeding 100 h; however, in acidic conditions, the concentration remained above 90% for only 50 h [16]. Temperature elevation above 0 °C markedly impacts the stability of antibiotics, as demonstrated by Binson et al., who noted that both the initial concentration and temperature significantly influence decomposition rates [12]. Specifically, they found that higher solution concentrations lead to greater decomposition, while increased temperatures correlate with diminished stability [15]. Amoxicillin’s decomposition at low concentrations adheres to pseudo-high-order kinetics [42]. Binson et al. observed that higher concentrations of amoxicillin decomposed more swiftly, with a 13% decomposition rate after 24 h at lower concentrations, compared to an 83% rate at the highest concentration. At 37 °C, the initial concentration fell to 16% within the same timeframe [15]. Furthermore, Tapia-Albarran and Villafuerte-Robles showed that pH levels are key; at 37 °C, in a solution with a pH of 1.2, amoxicillin’s half-life extends beyond 6 h, whereas at a pH of 7.4, this duration can increase significantly [18]. The susceptibility of amoxicillin’s β-lactam ring to hydrolytic degradation is well-documented, especially when the pH strays far from its isoelectric point (pH 4.8) [43]. Fawaz et al. reported that at 4 °C, the half-life in an UPW medium was 80.3 h, dropping to 24.8 h at 25 °C and to just 9 h at 37 °C [17]. Tarpia-Albarran et al. established the half-life in an acidic medium (pH 7.4) to be between 37.1 and 38.3 h, as determined by iodometric titration [18]. Hahne et al. found that incubation at 20 °C for 17 days resulted in a reduction to less than half of the original active substance concentration after 6 days, and by day 17, concentrations fell to below 10% [44].

For cefotaxime, the stability observed in the UPW stock solution closely mirrored that of concentrations diluted in TSB, showing that 70% of the drug was measurable after one day of incubation, 40% after two days, and an average of only 3.6% remained by day 12. Seraissol et al. noted that cefotaxime remained stable for 3 months at −20 °C and for 6 months when stored at −80 °C. It was also found to be stable in serum for 3 days at 4 °C and for 6 h at room temperature [45]. Conversely, an earlier study observed a reduction of more than 20% after just one week at −20 °C [30]. While cephalosporins exhibit general stability in their solid powder form, their UPW solutions are prone to hydrolysis, forming various degradation products, a process accelerated by increased temperatures [20]. The notable instability of cefotaxime in UPW solutions can be attributed to its chemical structure, with the β-lactam ring being particularly susceptible to hydrolysis [19]. Gáspár et al. reported less than 20% degradation of cefotaxime within the first 4 h at room temperature [20], Loeuille et al. reported stability lasting up to 6 h [23]. Qureshi et al. observed a 30% degradation within 30 h [22]. Iqbal et al. noted a 13% degradation over 72 h at lower concentrations, with degradation doubling at higher concentrations [21].

In our investigation, the neomycin UPW stock solution maintained relative stability, exceeding 90% throughout the study period. However, significant degradation was observed in TSB dilutions, with an average 30% decrease noted after just one day. This degradation progressed, resulting in only 29% of the initial drug concentration being detectable by day 12. Mascher et al. reported recovering 66.6% of the original neomycin concentration from human serum [46], but comparative studies on stability in stock solutions or TSB akin to ours are absent from the current literature.

For oxytetracycline, our findings also revealed a higher stability in the UPW stock solution, with 75% of the initial concentration remaining after two days, 50.6% after five days, and 37.6% after 12 days of incubation. Conversely, the dilutions in TSB exhibited more pronounced degradation, with only 46.6% of the drug present after two days, decreasing to 10.6% after five days, and plummeting to 2% by day 12. German et al. observed no significant degradation over a week at 2–8 °C, and stable solution concentrations after several decades of storage at −20 °C [28]. In contrast, Okerman et al. documented nearly 20% degradation after five months [29], while Llorca et al. reported a reduction of over 20% from the initial concentration after just one week [30]. Sah et al. conducted tests on an UPW solution of oxytetracycline at 37 °C and identified significant degradation, with a half-life of merely 34 h [31].

In our study, florfenicol exhibited remarkable stability, with the UPW stock solution and its dilutions in TSB maintaining nearly 100% of the initial concentration throughout the testing period. Similarly, Batrawi et al. reported minimal degradation (less than 2%) over 16 h at room temperature in UPW solution. However, under highly acidic conditions and at 40 °C, 10% of florfenicol degraded within two days, and 26% degraded in just two hours in a highly basic medium. Notably, only 7.5% of florfenicol degraded over 14 days at 75 °C [35], with similar resilience observed over two hours at 100 °C, showing less than 20% degradation [36]. Hayes et al. further demonstrated florfenicol’s stability, retaining over 90% of its initial concentration after 24 h at room temperature in UPW solution [47].

Like florfenicol, enrofloxacin showed considerable stability, with 88.7% of the initial concentration detectable on average by day 12 in both UPW solution and concentrations diluted in TSB. Okerman et al. found more than 90% of the initial concentration intact after six months at −20 °C [29], while Llorca et al. observed a 20% degradation after two weeks’ storage in similar conditions [30]. Metry et al. confirmed enrofloxacin’s stability, with no significant changes over 28 days at room temperature [48]. Marx et al., Park et al., and Petritz et al. conducted additional tests at room temperature, finding enrofloxacin stable over 7 days [32], 28 days [34], and 56 days [33], respectively.

In our analysis, the stability of colistin in UPW stock solutions decreased to 89.2% of the initial value on day 1, with a gradual reduction to 40.5% by day 12. Dilutions in TSB exhibited more pronounced degradation, with only 24.1% of the initial concentration detectable by the study’s end. However, the stability of colistin appears variable. German et al. reported that UPW solutions of colistin remained stable for several decades at −20 °C [28]. and Li et al. noted no significant change in concentration after 60 days at 4 °C [25]. Similarly, Pfeifer et al. reported no degradation for at least six months at 2–8 °C, although instability was observed at 25 °C [49].

In contrast to Li et al. and Pfeifer et al.’s results, Yuan et al. observed a 13% degradation after one month of storage at 4 °C [50]. Barco et al. also documented colistin degradation in blood plasma; 13% within six hours, 4% across multiple freeze–thaws, 11% at −80 °C, and 8% at −20 °C, over four weeks [51]. Conversely, Matar et al. found acceptable stability in blood plasma even after multiple freeze–thaw cycles over 20 days [52].

Colistin, a cyclic heptapeptide with a tripeptide L-dallate chain linked to a fatty acid at the N-terminus, comprises two main components: colistin A (polymyxin E_1_) and colistin B (polymyxin E_2_) [25]. The stability of colistin is influenced by oxidation, reduction, hydrolysis, β-elimination, and racemization [24]. Orwa et al. identified racemization as a key factor in instability across acidic and basic pH levels, noting a half-life of several thousand hours at 37 °C in an acidic medium, but approximately 70 h at pH 7.4 [26]. This is supported by Li et al.’s finding of a significant degradation in isotonic phosphate buffer (pH 7.4) [25].

The stability of trimethoprim and sulfamethoxazole, when measured together as components of potentiated sulfonamide, exhibited notable similarity. Their UPW solutions demonstrated higher stability, with 96.2% of trimethoprim and 99.2% of sulfamethoxazole’s active substances remaining detectable by day 12. In contrast, TSB dilutions showed accelerated degradation, with an average of 91.1% of trimethoprim and 90.3% of sulfamethoxazole still present by day 12. In their study, Rehm et al. reported less than 15% degradation for the sulfamethoxazole–trimethoprim combination across various conditions: in plasma at 4 °C for 72 h, at room temperature for 24 h, at −70 °C for six months, and for the stock solution at −70 °C for twelve months [53]. Khaleel et al. confirmed its stability for four hours at room temperature, with over 98% of the original drug concentration retained [37]. Previous investigations into the stability of these UPW solutions at room temperature over seven days in acidic pH broth yielded variable results, likely due to the uneven distribution of the active ingredient, with a marked decrease observed in acidic conditions [54]. Hahne et al. explored the stability of a trimethoprim and sulfadiazine combination at 20 °C, over 70 days, finding the drugs remained stable in physiological saline, broth, distilled water, phosphate buffer, and formic acid solutions throughout the testing period [44].

## 4. Materials and Methods

### 4.1. Active Substances and Matrices

To prepare the stock solutions of the active substances provided by Merck KGaA, Darmstadt, Germany, we adhered to the specifications set out by the Clinical and Laboratory Standards Institute (CLSI) [55]. The quantity of each active substance required the stock solution to be adjusted for purity as indicated in the batch certification. The stock solutions were prepared in 40 mL volumes with UPW, which had pH 6.91, containing concentrations tenfold higher than each subsequent dilution. The UPW was produced using an ion exchange resin system followed by UV radiation treatment. An ultrasonic water bath ensured the complete dissolution of the substances. The methodology for stock solution preparation is detailed in Table 2. Following preparation, the solutions were filtered through a 0.2 µm cellulose filter (VWR International, LLC., Debrecen, Hungary) to ensure sterility. Sterile 50 mL centrifuge tubes were then filled with 18 mL of TSB (Biolab Zrt., Budapest, Hungary) which had buffered pH 7.3, under sterile conditions. To create the initial 10× dilution, the most concentrated—2 mL of stock solution was added to the first tube and thoroughly mixed. This process was repeated, sequentially diluting the solution 10×, 100×, 1000×, and finally 10,000×. The stock solution and each dilution were sampled and analyzed in triplicate immediately to serve as the zero-day (initial) samples. The centrifuge tubes were incubated at 37 °C and sampled for HPLC analysis on days 1, 2, 5, 7, 9, and 12, with each measurement conducted in triplicate.

Regarding solubility in UPW media, amoxicillin trihydrate exhibits a solubility of 1–10 mg/mL [56], cefotaxime sodium > 50 mg/mL [57], neomycin sulphate 100 mg/mL [58], oxytetracycline 313 mg/mL [59], florfenicol 0.219 mg/mL [60], enrofloxacin 0.612 mg/mL [61], colistin sulphate 50 mg/mL [62], a sulfamethoxazole 0.459 mg/mL [63] and trimethoprim 0.615 mg/mL [64].

### 4.2. Sample Preparation

Prior to HPLC analysis, samples collected on the designated measurement days required dilution to ensure that active substances were within the optimal concentration range for accurate assessment. The specific dilution ratios employed are comprehensively outlined in Supplementary Table S46. For the measurements, 1 mL of each diluted solution was used, with each analysis performed in triplicate to ensure the reliability and reproducibility of the results.

For the HPLC analysis of amoxicillin and cefotaxime, dilutions were exclusively made using UPW, which had a pH of 6.91, with 200 µL of methanol (MeOH) subsequently added to each 1 mL of solution. Oxytetracycline and potentiated sulfonamide solutions were similarly diluted with UPW, but with 300 µL of acetonitrile (ACN) added to 1 mL of each solution. Given the dual components of potentiated sulfonamide, two distinct dilutions were prepared to ensure both sulfamethoxazole and trimethoprim concentrations fell within the optimal range for accurate measurement. Enrofloxacin solutions were also diluted using UPW, with an addition of 200 µL ACN to each 1 mL of sample. For neomycin, a specialized diluent mirroring the eluent’s composition was required. This ‘H-solution’ consisted of 70% UPW, 30% ACN, and 0.1% *v*/*v* heptafluorobutyric acid (HFBA). Florfenicol solutions underwent dilution with a 3:1 ratio of water to ACN. The approach to diluting colistin solutions varied with the active substance concentration; solutions above 10 µg/mL were diluted solely with UPW, while those below this concentration employed a 4:1 ACN to water mixture, enhanced with 0.2% *v*/*v* formic acid (HCOOH).

### 4.3. Preparation of Calibration Solutions

For HPLC analysis, we employed a 5-point calibration curve, preparing solutions according to standard dilution series. As with the samples, 1 mL of these calibration solutions was utilized for each measurement. Active substances for stock solutions, supplied by Merck KGaA, Darmstadt, Germany, were dissolved in UPW for all substances except florfenicol, for which methanol (MeOH) was used. Given the dual active components of potentiated sulfonamide, two separate dilutions were prepared during calibration—one for sulfamethoxazole and one for trimethoprim—to ensure the accurate measurement of each.

The protocols for diluting and preparing these calibration solutions mirrored those employed for the sample solutions. Details regarding the concentrations of the standard dilution series for stock solutions and the calibration solutions are documented in Supplementary Table S47.

### 4.4. Liquid Chromatograph and Mass Spectrometer Parameters

Analytical measurements were conducted utilizing a SCIEX Exion LC™ 2.0 UHPLC (AB Sciex LLC, Framingham, MA, USA) system, interfaced with a SCIEX QTRAP 4500 (AB Sciex Pte. Ltd., Estate, Singapore) triple quadrupole mass spectrometry system. Chromatographic separation was achieved on a Merck Purospher^®^ STAR RP-18 column (Merck, Darmstadt, Germany), featuring a 3 µm particle size, across all analyses (150 × 4.6 mm: amoxicillin, florfenicol, oxytetracycline, sulfamethoxazole, trimethoprim; 150 × 3 mm: cefotaxime; 100 × 4.6 mm: colistin, enrofloxacin, neomycin).

Isocratic elution facilitated the separation for all tested drugs, except neomycin, which necessitated gradient elution to ensure adequate separation from the nutrient solution, and to yield a precise, prominent chromatographic peak. The sample compartment temperature was maintained at 5 °C. The detailed settings employed for the chromatographic analysis of each active substance are delineated in Table 3. LC-MS grade solvents supplied by Merck KGaA (Darmstadt, Germany), were used for the mobile phases.

Electrospray ionization (ESI) in positive mode was used for mass spectrometric detection. Operational parameters for the mass spectrometer were uniform across all compounds, with variations only in the ion source temperature. The system operated at a gas pressure of 40 psi, an inlet potential of +10 V, and an ionization voltage of 5500 V. Ion source temperatures were set at 450 °C for sulfamethoxazole, florfenicol, colistin, and enrofloxacin; 600 °C for oxytetracycline; 650 °C for amoxicillin and neomycin; and 700 °C for cefotaxime analysis. Ion transitions monitored by the mass spectrometer for each compound are available in Supplementary Table S48.

Repeatability has been tested, which refers to the ability of a measurement procedure to produce consistent results when the same experiment is performed under identical conditions within a short period of time. This includes the use of the same instruments, methods, operators, and measurement locations. Repeatability indicates the stability and reliability of the measurement process and is typically quantified by the dispersion or relative dispersion of the measurement results, such as relative standard deviation. The Limit of Detection (LOD) refers to the lowest concentration of an analyte that can be reliably distinguished from background noise but not necessarily quantified as an exact value. The Limit of Quantification (LOQ) is the lowest concentration of an analyte that can be quantitatively determined with acceptable precision and accuracy. Precision refers to the ability of a measurement procedure to yield similar results under consistent conditions over multiple trials. It indicates the degree to which repeated measurements under unchanged conditions show the same results. Precision is concerned with the variability or spread of the measurement results, reflecting the consistency and reproducibility of the measurements, regardless of their proximity to the true or accepted value. System suitability refers to the evaluation of an analytical system’s capability to perform a specific analytical task. This test ensures that the entire analytical system, including instruments, reagents, and methods, is functioning properly and is suitable for the intended analytical purpose. Parameters characterizing system suitability include peak resolution, peak symmetry, signal-to-noise ratio, repeatability, and reproducibility. These parameters are regularly assessed to ensure that the analytical system produces stable and reliable results [65,66]. The LOD and LOQ limits were determined by measuring blank samples and evaluating the noise peaks observed around the retention time (7 parallels). The LOD was defined as three times the average noise level, and the LOQ as ten times the average noise level.

## 5. Conclusions

In summary, our study demonstrated that neomycin, florfenicol, and potentiated sulfonamide exhibit long-term stability in UPW media at an incubation temperature of 37 °C. Conversely, a significant reduction in drug concentration was observed in TSB, with amoxicillin experiencing an average 50% decrease and cefotaxime, neomycin, oxytetracycline, and colistin showing 25–30% reductions after just one day of incubation. These findings are crucial for short-term studies at 37 °C, such as those determining minimum inhibitory concentrations (MIC) over 18–24 h or minimum bactericidal concentrations (MBC) over 72 h, which are often conducted in TSB.

Moving forward, it would be beneficial to expand the scope of our investigations to include a broader array of active substances and to replicate these studies using different sources of TSB. The insights garnered from our research can significantly enhance the accuracy of susceptibility testing, offering a more dependable assessment of antimicrobial resistance, which is essential for public health and safety.

## Figures and Tables

**Table 1:** Equation and explanatory power of the logarithmic trend line of means and their non-parametric statistical Kruskal–Wallis H-test.

Active Substance	Equation	Explanatory Power	*p*-Value	
			Day	Dilution
Amoxicillin	y = −0.495ln(x) + 0.9096	R^2^ = 0.9363	<0.0001 *	0.9784
Cefotaxime	y = −0.548ln(x) + 1.0193	R^2^ = 0.9602	<0.0001 *	0.5722
Neomycin	y = −0.350ln(x) + 0.9881	R^2^ = 0.9930	0.0011 *	0.0001 *
Oxytetracycline	y = −0.571ln(x) + 1.0413	R^2^ = 0.9468	<0.0001 *	0.8379
Florfenicol	y = 0.0133ln(x) + 0.9875	R^2^ = 0.4948	0.3484	<0.0001 *
Enrofloxacin	y = −0.068ln(x) + 1.0128	R^2^ = 0.8609	0.0389 *	0.0028 *
Colistin	y = −0.377ln(x) + 0.9575	R^2^ = 0.9828	<0.0001 *	0.0054 *
Sulfamethoxazole	y = −0.051ln(x) + 1.0346	R^2^ = 0.6164	<0.0001 *	0.3279
Trimethoprim	y = −0.047ln(x) + 1.0232	R^2^ = 0.6513	<0.0001 *	0.1371

* significant difference (*p* < 0.05).

**Table 2:** Concentrations of stock solutions and compounds used for their preparation.

Active Substance	Applied Compound	Purity of the Applied Compound	Target Concentration of Stock Solution	Target Amount of Compound	Compound(s) Suspended for Dissolution
Amoxicillin	Amoxicillin trihydrate	86.0%	10,000 µg/mL	11,628 µg/mL	0.1 mol/L phosphate buffer
Cefotaxime	Cefotaxime sodium	89.1%	5000 µg/mL	5612.5 µg/mL	UPW
Neomycin	Neomycin sulphate	60.9%	40,000 µg/mL	65,680 µg/mL	UPW
Oxytetracycline	Oxytetracycline	100%	5000 µg/mL	5000 µg/mL	UPW
Florfenicol	Florfenicol	99.6%	40,000 µg/mL	40,160 µg/mL	UPW + 5% 96% ethanol
Enrofloxacin	Enrofloxacin	98%	150 µg/mL	153 µg/mL	UPW + 10% 0.1 mol sodium hydroxide
Colistin	Colistin sulphate	71.6%	2500 µg/mL	3490 µg/mL	UPW
Potential sulfonamide	Sulfamethoxazole	99.9%	19,047.5 µg/mL *	19,066.5 µg/mL	Warm UPW + 5% 2.5 mol NAOH
	Trimethoprim	99.9%	952.5 µg/mL *	953.8 µg/mL	UPW + 5% 0.05 mol HCl

* the stock solution concentration target for potentiated sulfonamide was 20,000 µg/mL using a 20:1 ratio sulfamethoxazole–trimethoprim mixture; UPW—ultrapure water.

**Table 3:** Settings used for different active substances during HPLC measurement.

Active Substance	Separation Procedure	Mobile Phase Composition		Column Diameter (mm)	Column Temperature (°C)	Flow Rate (mL/min)	Injection Volume (µL)	Measuring Time (min)
		“A”	“B”					
Amoxicillin	Isocratic; 95% “A”, 5% “B”	UPW + 0.2 V/V% HCOOH + 5 mM NH_4_OOCH	ACN	150 × 4.6	35	1.0	50	6
Cefotaxime	Isocratic; 80% “A”, 20% “B”	UPW + 0.1 V/V% HCOOH + 5 mM NH_4_OOCH	MeOH	150 × 3.0	45	0.8	25	7.5
Neomycin	Gradient (t/min) t_0_, t_1_: 70% “A”, 30% “B” t_11_, t_12_: 5% “A”, 95% “B” t_13_, t_15_: 70% “A”, 30% “B”	UPW + 0.1 V/V% HFBA	ACN + 0.1 V/V% HFBA	100 × 4.6	30	0.3	25	15
Oxytetracycline	Isocratic; 75% “A”, 25% “B”	UPW + 0.1 V/V% HCOOH	ACN + 0.1 V/V% HCOOH	150 × 4.6	45	1.0	20	5
Florfenicol	Isocratic; 75% “A”, 25% “B”	UPW + 0.1 V/V% HCOOH + 2 mM NH_4_OOCH	ACN	150 × 4.6	45	1.0	10	10
Enrofloxacin	Isocratic; 80% “A”, 20% “B”	UPW + 0.1 V/V% HCOOH + 2 mM NH_4_OOCH	ACN	100 × 4.6	45	1.0	10	6
Colistin	Isocratic; 20% “A”, 80% “B”	UPW + 0.2 V/V% HCOOH	ACN + 0.2 V/V% HCOOH	100 × 4.6	30	0.5	50	6
Sulfamethoxazole + trimethoprim	Isocratic; 70% “A”, 30% “B”	UPW + 0.1 V/V% HCOOH	ACN	150 × 4.6	45	1.0	25	6.5

HCOOH—formic acid; NH_4_OOCH—ammonium formate; HFBA—heptafluorobutyric acid; ACN—acetonitrile; MeOH—methanol; UPW—ultrapure water.

## Data Availability

The datasets used and/or analyzed during the current study are available from the corresponding author on reasonable request.

## Supplementary Materials

The following supporting information can be downloaded at: https://www.mdpi.com/article/10.3390/antibiotics13060549/s1.
