# Supplementary material for: Stability Studies of the Dilution Series of Different Antibiotic Stock Solutions in Culture Medium Incubated at 37 °C
Source: Antibiotics (Basel). 2024 Jun 12;13(6):549. doi: 10.3390/antibiotics13060549 (PMC11200527; doi:10.3390/antibiotics13060549)
Supplement: Supplementary file 1 [file antibiotics-13-00549-s001.zip › antibiotics-3008601-supplementary.pdf]

Supplementary Table S1 Analytical reliability of amoxicillin (AMX) samples. The standard deviation (Stdev) measures the deviation of individual measurements from the mean. Low values of the relative standard deviation (RSD%) indicate that measurements are consistent.

Sample	Area / counts	c (AMX base; STD) (ng/mL)	c (AMX base; sample) (µg/mL)	c (AMX base; sample) mean (µg/mL)	Stdev	RSD%
Day0-STOCK SOLUTION-1	93755	182.82	4570.62	4728.42	166.97	3.53
Day0-STOCK SOLUTION-2	96642	188.46	4711.38			
Day0-STOCK SOLUTION-3	100578	196.13	4903.26			
Day0-1000 µg/mL-1	102685	200.24	1001.19	985.95	34.18	3.47
Day0-1000 µg/mL-2	97106	189.36	946.80			
Day0-1000 µg/mL-3	103573	201.97	1009.85			
Day0-100µg/mL-1	99403	193.84	96.92	96.41	1.07	1.11
Day0-100 µg/mL-2	99620	194.26	97.13			
Day0-100 µg/mL-3	97614	190.35	95.18			
Day0-10 µg/mL-1	56232	109.65	10.97	11.25	0.38	3.34
Day0-10 µg/mL-2	56966	111.09	11.11			
Day0-10 µg/mL-3	59872	116.75	11.68			
Day0-1 µg/mL-1	59137	115.32	1.15	1.15	0.01	0.75
Day0-1 µg/mL-2	58262	113.61	1.14			
Day0-1 µg/mL-3	58817	114.69	1.15			
Day1-STOCK SOLUTION-1	79899	155.24	3881.07	3832.83	42.70	1.11
Day1-STOCK SOLUTION-2	78591	152.70	3817.56			
Day1-STOCK SOLUTION-3	78227	151.99	3799.87			
Day1-1000 µg/mL-1	52005	101.05	505.23	515.99	32.05	6.21
Day1-1000 µg/mL-2	56823	110.41	552.03			
Day1-1000 µg/mL-3	50509	98.14	490.70			
Day1-100µg/mL-1	51818	100.68	50.34	51.15	0.77	1.50
Day1-100 µg/mL-2	53394	103.75	51.87			
Day1-100 µg/mL-3	52746	102.48	51.24			
Day1-10 µg/mL-1	31250	60.72	6.07	6.06	0.03	0.55
Day1-10 µg/mL-2	31008	60.25	6.02			
Day1-10 µg/mL-3	31311	60.84	6.08			
Day1-1 µg/mL-1	34600	67.23	0.67	0.65	0.04	6.23
Day1-1 µg/mL-2	34274	66.59	0.67			
Day1-1 µg/mL-3	30861	59.96	0.60			
Day2-STOCK SOLUTION-1	33637	104.71	2617.76	2563.98	90.75	3.54
Day2-STOCK SOLUTION-2	33601	104.60	2614.97			

Day2-STOCK SOLUTION-3	31600	98.37	2459.21			
Day2-1000 μg/mL-1	17025	53.00	264.98	271.22	5.40	1.99
Day2-1000 μg/mL-2	17625	54.87	274.33			
Day2-1000 μg/mL-3	17626	54.87	274.35			
Day2-100μg/mL-1	15097	47.00	23.50	23.75	0.84	3.53
Day2-100 μg/mL-2	14815	46.12	23.06			
Day2-100 μg/mL-3	15856	49.36	24.68			
Day2-10 μg/mL-1	8616	26.82	2.68	2.60	0.08	2.89
Day2-10 μg/mL-2	8269	25.74	2.57			
Day2-10 μg/mL-3	8152	25.38	2.54			
Day2-1 μg/mL-1	7162	22.29	0.22	0.21	0.02	7.66
Day2-1 μg/mL-2	6142	19.12	0.19			
Day2-1 μg/mL-2	6671	20.77	0.21			
Day6-STOCK SOLUTION-1	22522	53.76	1344.02	1340.23	15.73	1.17
Day6-STOCK SOLUTION-2	22169	52.92	1322.96			
Day6-STOCK SOLUTION-3	22685	54.15	1353.73			
Day6-1000 μg/mL-1	10805	25.79	128.96	124.77	3.95	3.16
Day6-1000 μg/mL-2	10409	24.85	124.23			
Day6-1000 μg/mL-3	10148	24.22	121.12			
Day6-100μg/mL-1	10073	24.04	12.02	12.29	0.24	1.95
Day6-100 μg/mL-2	10344	24.69	12.35			
Day6-100 μg/mL-3	10466	24.98	12.49			
Day6-10 μg/mL-1	6597	15.75	1.57	1.53	0.05	3.09
Day6-10 μg/mL-2	6377	15.22	1.52			
Day6-10 μg/mL-3	6202	14.80	1.48			
Day6-1 μg/mL-1	6385	15.24	0.15	0.16	0.02	9.37
Day6-1 μg/mL-2	7477	17.85	0.18			
Day6-1 μg/mL-3	6380	15.23	0.15			
Day7-STOCK SOLUTION-1	12593	36.67	916.84	911.16	14.07	1.54
Day7-STOCK SOLUTION-2	12295	35.81	895.14			
Day7-STOCK SOLUTION-3	12657	36.86	921.50			
Day7-1000 μg/mL-1	7254	21.13	105.63	105.57	5.05	4.78
Day7-1000 μg/mL-2	7595	22.12	110.59			
Day7-1000 μg/mL-3	6902	20.10	100.49			
Day7-100μg/mL-1	6506	18.95	9.47	9.88	0.50	5.01
Day7-100 μg/mL-2	7164	20.86	10.43			
Day7-100 μg/mL-3	6686	19.47	9.74			
Day7-10 μg/mL-1	3887	11.32	1.13	1.17	0.03	2.87

Day7-10 µg/mL-2	4115	11.98	1.20			
Day7-10 µg/mL-3	4026	11.72	1.17			
Day7-1 µg/mL-1	4316	12.57	0.13	0.13	0.01	4.78
Day7-1 µg/mL-2	4749	13.83	0.14			
Day7-1 µg/mL-3	4560	13.28	0.13			
Day9-STOCK SOLUTION-1	7000	20.30	507.42	516.71	9.13	1.77
Day9-STOCK SOLUTION-2	7252	21.03	525.67			
Day9-STOCK SOLUTION-3	7133	20.68	517.03			
Day9-1000 µg/mL-1	4810	13.95	69.74	70.38	1.42	2.02
Day9-1000 µg/mL-2	4787	13.88	69.40			
Day9-1000 µg/mL-3	4968	14.40	72.01			
Day9-100µg/mL-1	4679	13.57	6.78	6.97	0.20	2.94
Day9-100 µg/mL-2	4958	14.38	7.19			
Day9-100 µg/mL-3	4780	13.86	6.93			
Day9-10 µg/mL-1	2623	7.60	0.76	0.77	0.05	6.82
Day9-10 µg/mL-2	2498	7.24	0.72			
Day9-10 µg/mL-3	2855	8.28	0.83			
Day9-1 µg/mL-1	2717	7.88	0.08	0.08	0.00	3.67
Day9-1 µg/mL-2	2908	8.43	0.08			
Day9-1 µg/mL-3	2750	7.97	0.08			
Day12-STOCK SOLUTION-1	4245	12.14	303.48	288.49	13.79	4.78
Day12-STOCK SOLUTION-2	3865	11.05	276.35			
Day12-STOCK SOLUTION-3	3995	11.43	285.65			
Day12-1000 µg/mL-1	3177	9.09	45.43	46.85	1.29	2.76
Day12-1000 µg/mL-2	3297	9.43	47.14			
Day12-1000 µg/mL-3	3355	9.59	47.97			
Day12-100µg/mL-1	3163	9.05	4.52	4.67	0.15	3.31
Day12-100 µg/mL-2	3253	9.30	4.65			
Day12-100 µg/mL-3	3378	9.66	4.83			
Day12-10 µg/mL-1	1989	5.69	0.57	0.56	0.01	1.99
Day12-10 µg/mL-2	1918	5.48	0.55			
Day12-10 µg/mL-3	1981	5.67	0.57			
Day12-1 µg/mL-1	2250	6.43	0.06	0.07	0.00	4.50
Day12-1 µg/mL-2	2459	7.03	0.07			
Day12-1 µg/mL-3	2334	6.67	0.07			

Supplementary Table S2 Standard curve data for the active substance amoxicillin (AMX). A calibration curve based on the concentrations and their corresponding peak areas can be used to determine the concentration of substances in the samples.

**STD concentration (ng/mL)**	**Peak area (Int unit)**	**Linearity (y=ax+b)**	
502	22919100	a (slope)	512.81238
200.8	9066570	b (intercept)	0
100.4	4615940	r	0.99999
50.2	2357230	r^2	0.99998
20.08	987497	Requirement: r ≥ 0.99	

STD - standard solutions concentration; a - slope; b - y-axis intercept; r - correlation coefficient; r2 - coefficient of determination

Supplementary Table S3 Repeatability test data for the active substance amoxicillin (AMX) in the analytical assay. It provides information on the consistency of the replicates, which is important for assessing the reliability of analytical measurements. The low CV% value shown here is favorable because it indicates that the analytical process is stable and repeatable.

**Repeatability**		
Sample	Area	c (AMX base; STD) (ng/mL)
Repeat1	50509	98.49
Repeat2	49952	97.41
Repeat3	51384	100.20
Repeat4	49843	97.20
Repeat5	48033	93.66
Repeat6	49467	96.46
Requirement: CV% < 10	Mean	97.24
	SD	2.18
	CV%	2.24

SD - standard deviation; CV% - variance between replicates

Supplementary Table S4 Determination of amoxicillin (AMX) limits. These data are key to assessing the sensitivity and accuracy of the analytical method. Low LOD (limit of detection) and LOQ (limit of quantitation) values indicate good sensitivity, but high CV% suggests that background noise shows considerable variability, which may affect the accuracy of determining very low concentrations.

**LOD, LOQ**				
Sample	Area	c (AMX base; STD) (ng/mL)	LOD (ng/mL)	LOQ (ng/mL)
Noise1	48	0.09	0.43	1.44
Noise2	61	0.12		
Noise3	64	0.12		
Noise4	92	0.18		
Noise5	105	0.20		
Noise6	43	0.08		
Noise7	105	0.21		
Mean	74	0.14		
SD	26.20	0.05		
CV%	35.4	35.4		

SD - standard deviation; CV% - variance between replicates

Supplementary Table S5 System assay for the measurement of the active substance amoxicillin (AMX). The CV% values are quite low, suggesting that the system is performing relatively consistently, the chromatography system is working properly and is suitable for analysis.

System suitability			
*t*0 (min) = 0.923			
	k'	N	Asymmetry factor
STD-500	3.061	6908	1.52
STD-200	3.082	5536	1.51
STD-100	3.086	5548	1.67
Mean	3.076	5997	1.57
SD	0.014	789	0.09
CV%	0.440	13.2	5.72
k'	> 2		
N	≥ 2000		
Asymmetry factor	≤ 2		

t0 – dead time; k' – capacity factor; N – theoretical number of plates; SD - standard deviation; CV% - variance between replicates

Supplementary Table S6 Analytical reliability of cefotaxime (CTXM) samples. The standard deviation (Stdev) measures the deviation of individual measurements from the mean. Low values of the relative standard deviation (RSD%) indicate that measurements are consistent.

Sample	Peak area (counts)	c (CTXM basis; STD) (ng/mL)	c (CTXM basis; Sample) (µg/mL)	c (CTXM basis; Sample) mean (µg/mL)	STDEV	CV%
Day0-STOCK SOLUTION-1	868543	47.76	4776.27	4762.10	13.81	0.3
Day0-STOCK SOLUTION-2	865830	47.61	4761.35			
Day0-STOCK SOLUTION-3	863525	47.49	4748.68			
Day0-500 µg/mL-1	921216	50.66	506.59	511.97	7.24	1.4
Day0-500 µg/mL-2	945964	52.02	520.20			
Day0-500 µg/mL-3	925824	50.91	509.13			
Day0-50 µg/mL-1	1000790	55.04	55.04	54.59	0.39	0.7
Day0-50 µg/mL-2	988035	54.33	54.33			
Day0-50 µg/mL-3	989335	54.41	54.41			
Day0-5 µg/mL-1	986826	54.27	5.43	5.60	0.19	3.4
Day0-5 µg/mL-2	1012790	55.70	5.57			
Day0-5 µg/mL-3	1055320	58.03	5.80			
Day0-0.5 µg/mL-1	201214	11.07	0.55	0.58	0.03	5.1
Day0-0.5 µg/mL-2	221255	12.17	0.61			
Day0-0.5 µg/mL-3	205298	11.29	0.56			
Day1-STOCK SOLUTION-1	603782	32.34	3234.07	3192.96	73.32	2.3

Day1-STOCK SOLUTION-2	580303	31.08	3108.31			
Day1-STOCK SOLUTION-3	604235	32.36	3236.50			
Day1-500 µg/mL-1	674195	36.11	361.12	359.54	2.24	0.6
Day1-500 µg/mL-2	673074	36.05	360.52			
Day1-500 µg/mL-3	666467	35.70	356.98			
Day1-50 µg/mL-1	705955	37.81	37.81	37.93	0.94	2.5
Day1-50 µg/mL-2	726835	38.93	38.93			
Day1-50 µg/mL-3	691768	37.05	37.05			
Day1-5 µg/mL-1	714873	38.29	3.83	3.98	0.16	4.1
Day1-5 µg/mL-2	774808	41.50	4.15			
Day1-5 µg/mL-3	739830	39.63	3.96			
Day1-0.5 µg/mL-1	165361	8.86	0.44	0.46	0.01	3.1
Day1-0.5 µg/mL-2	170886	9.15	0.46			
Day1-0.5 µg/mL-3	176071	9.43	0.47			
Day2-STOCK SOLUTION-1	352564	19.10	1910.25	1907.50	77.18	4.0
Day2-STOCK SOLUTION-2	366042	19.83	1983.27			
Day2-STOCK SOLUTION-3	337567	18.29	1828.99			
Day2-500 µg/mL-1	411934	22.32	223.19	221.48	4.61	2.1
Day2-500 µg/mL-2	415259	22.50	224.99			
Day2-500 µg/mL-3	399141	21.63	216.26			
Day2-50 µg/mL-1	428626	23.22	23.22	23.66	0.44	1.9
Day2-50 µg/mL-2	445038	24.11	24.11			
Day2-50 µg/mL-3	436619	23.66	23.66			
Day2-5 µg/mL-1	457258	24.77	2.48	2.51	0.09	3.5
Day2-5 µg/mL-2	481995	26.12	2.61			
Day2-5 µg/mL-3	451959	24.49	2.45			
Day2-0.5 µg/mL-1	87650	4.75	0.24	0.24	0.00	1.4
Day2-0.5 µg/mL-2	89962	4.87	0.24			
Day2-0.5 µg/mL-3	89509	4.85	0.24			
Day5-STOCK SOLUTION-1	104299	5.70	570.16	596.45	34.25	5.7
Day5-STOCK SOLUTION-2	116192	6.35	635.17			
Day5-STOCK SOLUTION-3	106832	5.84	584.01			
Day5-500 µg/mL-1	126185	6.90	68.98	65.43	4.08	6.2
Day5-500 µg/mL-2	111531	6.10	60.97			
Day5-500 µg/mL-3	121375	6.64	66.35			
Day5-50 µg/mL-1	129478	7.08	7.08	6.82	0.32	4.6
Day5-50 µg/mL-2	118346	6.47	6.47			
Day5-50 µg/mL-3	126554	6.92	6.92			
Day5-5 µg/mL-1	130115	7.11	0.71	0.72	0.01	1.2

Day5-5 µg/mL-2	133124	7.28	0.73			
Day5-5 µg/mL-3	130487	7.13	0.71			
Day5-0.5 µg/mL-1	30535	1.67	0.08	0.08	0.00	3.6
Day5-0.5 µg/mL-2	29011	1.59	0.08			
Day5-0.5 µg/mL-3	31121	1.70	0.09			
Day7-STOCK SOLUTION-1	56528	3.14	314.45	331.01	15.10	4.6
Day7-STOCK SOLUTION-2	61844	3.44	344.02			
Day7-STOCK SOLUTION-3	60145	3.35	334.56			
Day7-500 µg/mL-1	64237	3.57	35.73	36.28	0.48	1.3
Day7-500 µg/mL-2	65669	3.65	36.53			
Day7-500 µg/mL-3	65772	3.66	36.59			
Day7-50 µg/mL-1	69865	3.89	3.89	3.93	0.05	1.3
Day7-50 µg/mL-2	70449	3.92	3.92			
Day7-50 µg/mL-3	71618	3.98	3.98			
Day7-5 µg/mL-1	77624	4.32	0.43	0.42	0.01	2.5
Day7-5 µg/mL-2	74656	4.15	0.42			
Day7-5 µg/mL-3	74034	4.12	0.41			
Day7-0.5 µg/mL-1	22335	1.24	0.06	0.06	0.00	3.0
Day7-0.5 µg/mL-2	23658	1.32	0.07			
Day7-0.5 µg/mL-3	23408	1.30	0.07			
Day9-STOCK SOLUTION-1	29033	1.65	164.63	156.75	8.04	5.1
Day9-STOCK SOLUTION-2	26199	1.49	148.56			
Day9-STOCK SOLUTION-3	27698	1.57	157.05			
Day9-500 µg/mL-1	30687	1.74	17.40	17.16	0.50	2.9
Day9-500 µg/mL-2	29248	1.66	16.58			
Day9-500 µg/mL-3	30837	1.75	17.49			
Day9-50 µg/mL-1	30209	1.71	1.71	1.67	0.04	2.5
Day9-50 µg/mL-2	29026	1.65	1.65			
Day9-50 µg/mL-3	28878	1.64	1.64			
Day9-5 µg/mL-1	32860	1.86	0.19	0.18	0.01	3.4
Day9-5 µg/mL-2	31296	1.77	0.18			
Day9-5 µg/mL-3	33448	1.90	0.19			
Day9-0.5 µg/mL-1	18994	1.08	0.05	0.06	0.01	9.8
Day9-0.5 µg/mL-2	19522	1.11	0.06			
Day9-0.5 µg/mL-3	22685	1.29	0.06			
Day12-STOCK SOLUTION-1	18306	0.99	98.72	95.05	3.39	3.6
Day12-STOCK SOLUTION-2	17066	0.92	92.03			
Day12-STOCK SOLUTION-3	17503	0.94	94.39			
Day12-500 µg/mL-1	18993	1.02	10.24	10.25	0.49	4.7

Day12-500 µg/mL-2	19906	1.07	10.74			
Day12-500 µg/mL-3	18103	0.98	9.76			
Day12-50 µg/mL-1	17904	0.97	0.97	0.98	0.02	2.1
Day12-50 µg/mL-2	18630	1.00	1.00			
Day12-50 µg/mL-3	18024	0.97	0.97			
Day12-5 µg/mL-1	13694	0.74	0.07	0.08	0.00	4.2
Day12-5 µg/mL-2	14533	0.78	0.08			
Day12-5 µg/mL-3	14868	0.80	0.08			
Day12-0.5 µg/mL-1	19904	1.07	0.05	0.06	0.00	6.1
Day12-0.5 µg/mL-2	20130	1.09	0.05			
Day12-0.5 µg/mL-3	22190	1.20	0.06			

Supplementary Table S7 2 Standard curve data for the active substance cefotaxime (CTXM). A calibration curve based on the concentrations and their corresponding peak areas can be used to determine the concentration of substances in the samples.

**STD concentration (ng/mL)**	**Peak area (Int unit)**	**Linearity (y=ax+b)**	
100	1828070	a (slope)	18184.52951
50	891672	b (intercept)	0
20	358621	r	0.99995
10	181720	r^2	0.99990
5	94713	Requirement: r ≥ 0.99	

STD - standard solutions concentration; a - slope; b - y-axis intercept; r - correlation coefficient; r2 - coefficient of determination

Supplementary Table S8 Repeatability test data for the active substance cefotaxime (CTXM) in the analytical assay. It provides information on the consistency of the replicates, which is important for assessing the reliability of analytical measurements. The low CV% value shown here is favorable because it indicates that the analytical process is stable and repeatable.

**Repeatability**		
Sample	Area	c (CTXM; STD) (ng/mL)
Repeat1	841413	46.27
Repeat2	886653	48.76
Repeat3	890255	48.96
Repeat4	898320	49.40
Repeat5	857762	47.17
Repeat6	884695	48.65
Requirement: CV% < 10	Mean	48.20
	SD	1.21
	CV%	2.51

SD - standard deviation; CV% - variance between replicates

Supplementary Table S9 Determination of cefotaxime (CTXM) limits. These data are key to assessing the sensitivity and accuracy of the analytical method. Low LOD (limit of detection) and LOQ (limit of quantitation) values indicate good sensitivity, but high CV% suggests that background noise shows considerable variability, which may affect the accuracy of determining very low concentrations.

**LOD, LOQ**				
Sample	Area	c (CTXM; STD) (ng/mL)	LOD (ng/mL)	LOQ (ng/mL)
Noise1	240	0.01	0.05	0.15
Noise2	165	0.01		
Noise3	297	0.02		
Noise4	473	0.03		
Noise5	154	0.01		
Noise6	169	0.01		
Noise7	437	0.02		
Mean	276	0.02		
SD	132.36	0.01		
CV%	47.9	47.9		

SD - standard deviation; CV% - variance between replicates

Supplementary Table S10 System assay for the measurement of the active substance cefotaxime (CTXM). The CV% values are quite low, suggesting that the system is performing relatively consistently, the chromatography system is working properly and is suitable for analysis.

**System suitability**			
*t*0 (min) = 0.942			
	k'	N	Asymmetry factor
STD-100	5.037	10612	0.94
STD-50	5.040	10623	0.96
STD-20	5.016	10537	1.14
Mean	5.031	10591	1.01
SD	0.013	47	0.11
CV%	0.264	0.4	10.87
k'		> 2	
N		≥ 2000	
Asymmetry factor		≤ 2	

t0 – dead time; k' – capacity factor; N – theoretical number of plates; SD - standard deviation; CV% - variance between replicates

Supplementary Table S11 Analytical reliability of oxytetracycline (OTC) samples. The standard deviation (Stdev) measures the deviation of individual measurements from the mean. Low values of the relative standard deviation (RSD%) indicate that measurements are consistent.

Sample	Peak area (counts)	*c* (OTC basis; STD) (ng/mL)	*c* (OTC basis; Sample) (µg/mL)	*c* (OTC basis; Sample) mean (µg/mL)	STDEV	CV%
OTC-BASIC MATERIAL-D"0"-50ng/mL1	1025930	51.27	5126.62	5053.63	64.34	1.27
OTC-BASIC MATERIAL-D"0"-50ng/mL2	1006420	50.29	5029.12			
OTC-BASIC MATERIAL-D"0"-50ng/mL3	1001620	50.05	5005.14			
Day"0"-500 µg/mL1	999917	49.97	499.66	488.37	9.78	2.00
Day"0"-500 µg/mL2	966031	48.27	482.73			
Day"0"-500 µg/mL3	966032	48.27	482.73			
Day"0"-50µg/mL1	1055840	52.76	52.76	51.04	1.56	3.06
Day"0"-50µg/mL2	994916	49.72	49.72			
Day"0"-50µg/mL3	1013290	50.63	50.63			
Day"0"-5µg/mL1	1019290	50.93	5.09	5.15	0.08	1.50
Day"0"-5µg/mL2	1040170	51.98	5.20			
Day"0"-5µg/mL3	1049530	52.45	5.24			
Day"0"-0.5µg/mL1	435602	21.77	0.54	0.54	0.00	0.58
Day"0"-0.5µg/mL2	436432	21.81	0.55			
Day"0"-0.5µg/mL3	431676	21.57	0.54			
OTC-BASIC MATERIAL-D1-50ng/1	948314	48.15	4815.07	4916.44	90.49	1.84
OTC-BASIC MATERIAL-D1-50ng/2	982579	49.89	4989.05			
OTC-BASIC MATERIAL-D1-50ng/3	973945	49.45	4945.21			
Day1-500µg/mL1	756157	38.39	383.94	388.85	4.46	1.15
Day1-500µg/mL2	773295	39.26	392.64			
Day1-500µg/mL3	768054	39.00	389.98			
Day1-50µg/mL1	772252	39.21	39.21	38.59	0.83	2.16
Day1-50µg/mL2	741354	37.64	37.64			
Day1-50µg/mL3	766509	38.92	38.92			
Day1-5µg/mL1	754821	38.33	3.83	3.82	0.01	0.38
Day1-5µg/mL2	749088	38.03	3.80			
Day1-5µg/mL3	752598	38.21	3.82			
Day1-0.5µg/mL1	313586	15.92	0.40	0.41	0.01	2.74
Day1-0.5µg/mL2	330284	16.77	0.42			
Day1-0.5µg/mL3	326918	16.60	0.41			
OTC-BASIC MATERIAL-D2-50ng/1	493070	37.76	3775.53	3819.11	41.55	1.09

OTC-BASIC MATERIAL-D2-50ng/2	499337	38.24	3823.52			
OTC-BASIC MATERIAL-D2-50ng/3	503876	38.58	3858.27			
Day2-500μg/mL1	318113	24.36	243.59	249.84	8.96	3.59
Day2-500μg/mL2	334450	25.61	256.09			
Day2-500μg/mL3	340809	26.10	260.96			
Day2-50μg/mL1	304607	23.32	23.32	23.33	0.24	1.04
Day2-50μg/mL2	307861	23.57	23.57			
Day2-50μg/mL3	301546	23.09	23.09			
Day2-5μg/mL1	299074	22.90	2.29	2.32	0.03	1.26
Day2-5μg/mL2	305990	23.43	2.34			
Day2-5μg/mL3	299711	22.95	2.29			
Day2-0.5μg/mL1	125183	9.59	0.24	0.24	0.01	2.54
Day2-0.5μg/mL2	128714	9.86	0.25			
Day2-0.5μg/mL3	131614	10.08	0.25			
OTC-BASIC MATERIAL-D5-50ng/1	499242	25.65	2565.36	2559.49	20.36	0.80
OTC-BASIC MATERIAL-D5-50ng/2	501367	25.76	2576.28			
OTC-BASIC MATERIAL-D5-50ng/3	493692	25.37	2536.84			
Day5-500μg/mL1	93278	4.79	47.93	49.04	1.44	2.94
Day5-500μg/mL2	97605	5.02	50.15			
Day5-500μg/mL3	98525	5.06	50.63			
Day5-50μg/mL1	106122	5.45	5.45	5.38	0.07	1.33
Day5-50μg/mL2	103370	5.31	5.31			
Day5-50μg/mL3	104371	5.36	5.36			
Day5-5μg/mL1	94552	4.86	0.49	0.51	0.02	4.27
Day5-5μg/mL2	102669	5.28	0.53			
Day5-5μg/mL3	100557	5.17	0.52			
Day5-0.5μg/mL1	50571	2.60	0.06	0.07	0.00	3.03
Day5-0.5μg/mL2	52531	2.70	0.07			
Day5-0.5μg/mL3	53658	2.76	0.07			
OTC-BASIC MATERIAL-D7-50ng/1	439146	22.09	2209.16	2163.53	41.09	1.90
OTC-BASIC MATERIAL-D7-50ng/2	427779	21.52	2151.98			
OTC-BASIC MATERIAL-D7-50ng/3	423300	21.29	2129.44			
Day7-500μg/mL1	41589	2.09	20.92	21.38	0.47	2.22
Day7-500μg/mL2	43403	2.18	21.83			
Day7-500μg/mL3	42047	2.12	21.15			
Day7-50μg/mL1	49598	2.50	2.50	2.38	0.12	4.96
Day7-50μg/mL2	44919	2.26	2.26			
Day7-50μg/mL3	47655	2.40	2.40			
Day7-5μg/mL1	40396	2.03	0.20	0.21	0.00	2.41

Day7-5µg/mL2	42020	2.11	0.21			
Day7-5µg/mL3	42194	2.12	0.21			
Day7-0.5µg/mL1	22295	1.12	0.03	0.03	0.00	1.19
Day7-0.5µg/mL2	22794	1.15	0.03			
Day7-0.5µg/mL3	22378	1.13	0.03			
OTC-BASIC MATERIAL-D9-50ng/1	363639	18.93	1892.56	1905.43	19.34	1.01
OTC-BASIC MATERIAL-D9-50ng/2	364310	18.96	1896.06			
OTC-BASIC MATERIAL-D9-50ng/3	370383	19.28	1927.66			
Day9-500µg/mL1	19514	1.02	10.16	10.03	0.21	2.13
Day9-500µg/mL2	19012	0.99	9.90			
Day9-500µg/mL3	19827	1.03	10.32			
Day9-50µg/mL1	20902	1.09	1.09	1.08	0.01	0.76
Day9-50µg/mL2	20588	1.07	1.07			
Day9-50µg/mL3	20723	1.08	1.08			
Day9-5µg/mL1	19230	1.00	0.10	0.10	0.00	2.16
Day9-5µg/mL2	18660	0.97	0.10			
Day9-5µg/mL3	19453	1.01	0.10			
Day9-0.5µg/mL1	12929	0.67	0.02	0.02	0.00	3.44
Day9-0.5µg/mL2	13193	0.69	0.02			
Day9-0.5µg/mL3	12318	0.64	0.02			
OTC-BASIC MATERIAL-D12-50ng/1	369124	18.10	1809.64	1903.01	82.45	4.33
OTC-BASIC MATERIAL-D12-50ng/2	400975	19.66	1965.79			
OTC-BASIC MATERIAL-D12-50ng/3	394411	19.34	1933.61			
Day12-500µg/mL1	18669	0.92	9.15	9.59	0.44	4.63
Day12-500µg/mL2	20469	1.00	10.04			
Day12-500µg/mL3	19385	0.95	9.50			
Day12-50µg/mL1	16732	0.82	0.82	0.86	0.03	3.78
Day12-50µg/mL2	17936	0.88	0.88			
Day12-50µg/mL3	17810	0.87	0.87			
Day12-5µg/mL1	16533	0.81	0.08	0.09	0.01	10.01
Day12-5µg/mL2	20155	0.99	0.10			
Day12-5µg/mL3	17825	0.87	0.09			
Day12-0.5µg/mL1	12219	0.60	0.01	0.01	0.00	1.34
Day12-0.5µg/mL2	11951	0.59	0.01			
Day12-0.5µg/mL3	12242	0.60	0.02			

Supplementary Table S12 Standard curve data for the active substance oxytetracycline (OTC). A calibration curve based on the concentrations and their corresponding peak areas can be used to determine the concentration of substances in the samples.

**STD concentration (ng/mL)**	**Peak area (Int unit)**	**Linearity (y=ax+b)**	
100	2004840	a (slope)	20011.83967
50	993679	b (intercept)	0
20	400480	r	0.99999
10	196599	r^2	0.99998
5	102148	Requirement: r ≥ 0.99	

STD - standard solutions concentration; a - slope; b - y-axis intercept; r - correlation coefficient; r2 - coefficient of determination

Supplementary Table S13 Repeatability test data for the active substance oxytetracycline (OTC) in the analytical assay. It provides information on the consistency of the replicates, which is important for assessing the reliability of analytical measurements. The low CV% value shown here is favorable because it indicates that the analytical process is stable and repeatable.

**Repeatability**		
Sample	Peak area	c (OTC basis; STD) (ng/mL)
Repeat1	380203	19.00
Repeat2	372687	18.62
Repeat3	384502	19.21
Repeat4	408597	20.42
Repeat5	394594	19.72
Repeat6	409806	20.48
Requirement: CV% < 10	Mean	19.57
	SD	0.76
	CV%	3.90

SD - standard deviation; CV% - variance between replicates

Supplementary Table S14 Determination of oxytetracycline (OTC) limits. These data are key to assessing the sensitivity and accuracy of the analytical method. Low LOD (limit of detection) and LOQ (limit of quantitation) values indicate good sensitivity, but high CV% suggests that background noise shows considerable variability, which may affect the accuracy of determining very low concentrations.

LOD, LOQ				
Sample	Peak area	c (OTC basis; STD) (ng/mL)	LOD (ng/mL)	LOQ (ng/mL)
Noise1	893	0.04	0.14	0.48
Noise2	930	0.05		
Noise3	926	0.05		
Noise4	889	0.04		
Noise5	1251	0.06		
Noise6	761	0.04		
Noise7	1088	0.05		
**Mean**	962	0.05		
**SD**	159.40	0.01		
**CV%**	16.6	16.6		

SD - standard deviation; CV% - variance between replicates

Supplementary Table S15 System assay for the measurement of the active substance oxytetracycline (OTC). The CV% values are quite low, suggesting that the system is performing relatively consistently, the chromatography system is working properly and is suitable for analysis.

**System suitability**			
*t*0 (min) = 0.808			
	k'	N	Asymmetry factor
STD-100	2.235	8960	1.04
STD-50	2.235	8960	1.03
STD-20	2.235	8960	1.04
**Mean**	2.235	8960	1.04
**SD**	0.000	0	0.01
**CV%**	0.000	0.0	0.56
k'		> 2	
N		≥ 2000	
Asymmetry factor		≤ 2	

t0 – dead time; k' – capacity factor; N – theoretical number of plates; SD - standard deviation; CV% - variance between replicates

Supplementary Table S16 Analytical reliability of florfenicol (FLO) samples. The standard deviation (Stdev) measures the deviation of individual measurements from the mean. Low values of the relative standard deviation (RSD%) indicate that measurements are consistent.

**Sample**	**Peak area / counts**	**c (FLO basis; STD) (ng/mL)**	**c (FLO basis; Sample) (µg/mL)**	**c (FLO basis; Sample) mean (µg/mL)**	**STDEV**	**CV %**
Day0-STOCK SOLUTION-1	905879	193.80	19379.62	19468.11	130.8	0.7
Day0-STOCK SOLUTION-2	917040	196.18	19618.39			
Day0-STOCK SOLUTION-3	907127	194.06	19406.32			
Day0-4000 µg/mL-1	915996	195.96	3919.21	3916.59	20.3	0.5
Day0-4000 µg/mL-2	910351	194.75	3895.06			
Day0-4000 µg/mL-3	919801	196.77	3935.49			
Day0-400 µg/mL-1	1039840	222.45	444.91	444.09	7.0	1.6
Day0-400 µg/mL-2	1053310	225.34	450.67			
Day0-400 µg/mL-3	1020610	218.34	436.68			
Day0-40 µg/mL-1	1029510	220.24	44.05	44.03	1.3	2.9
Day0-40 µg/mL-2	1058810	226.51	45.30			
Day0-40 µg/mL-3	998752	213.66	42.73			
Day0-4 µg/mL-1	623712	133.43	5.34	5.25	0.1	1.8
Day0-4 µg/mL-2	614544	131.47	5.26			
Day0-4 µg/mL-3	601526	128.69	5.15			
Day1-STOCK SOLUTION-1	910250	195.03	19502.96	19477.11	47.8	0.2
Day1-STOCK SOLUTION-2	906469	194.22	19421.95			

Day1-STOCK SOLUTION-3	910412	195.06	19506.43			
Day1-4000 µg/mL-1	832253	178.32	3566.36	3700.90	131.9	3.6
Day1-4000 µg/mL-2	893756	191.50	3829.91			
Day1-4000 µg/mL-3	864942	185.32	3706.44			
Day1-400 µg/mL-1	1041090	223.06	446.13	435.01	11.7	2.7
Day1-400 µg/mL-2	1017710	218.05	436.11			
Day1-400 µg/mL-3	986660	211.40	422.80			
Day1-40 µg/mL-1	1048320	224.61	44.92	45.19	1.5	3.4
Day1-40 µg/mL-2	1022270	219.03	43.81			
Day1-40 µg/mL-3	1092810	234.14	46.83			
Day1-4 µg/mL-1	621569	133.18	5.33	5.26	0.1	1.5
Day1-4 µg/mL-2	615740	131.93	5.28			
Day1-4 µg/mL-3	603407	129.29	5.17			
Day2-STOCK SOLUTION-1	889258	195.63	19562.74	19931.84	348.5	1.7
Day2-STOCK SOLUTION-2	908112	199.78	19977.51			
Day2-STOCK SOLUTION-3	920738	202.55	20255.27			
Day2-4000 µg/mL-1	805652	177.23	3544.70	3641.55	101.0	2.8
Day2-4000 µg/mL-2	851445	187.31	3746.18			
Day2-4000 µg/mL-3	825898	181.69	3633.78			
Day2-400 µg/mL-1	1025150	225.52	451.04	454.37	3.0	0.7
Day2-400 µg/mL-2	1038440	228.45	456.89			
Day2-400 µg/mL-3	1034560	227.59	455.18			
Day2-40 µg/mL-1	1047570	230.45	46.09	45.76	0.8	1.7
Day2-40 µg/mL-2	1019910	224.37	44.87			
Day2-40 µg/mL-3	1052460	231.53	46.31			
Day2-4 µg/mL-1	633351	139.33	5.57	5.33	0.2	3.9
Day2-4 µg/mL-2	593110	130.48	5.22			
Day2-4 µg/mL-3	592173	130.27	5.21			
Day5-STOCK SOLUTION-1	877503	194.76	19476.45	19751.33	317.5	1.6
Day5-STOCK SOLUTION-2	905543	200.99	20098.80			
Day5-STOCK SOLUTION-3	886617	196.79	19678.73			
Day5-4000 µg/mL-1	812420	180.32	3606.38	3658.14	56.0	1.5
Day5-4000 µg/mL-2	822358	182.52	3650.50			
Day5-4000 µg/mL-3	837461	185.88	3717.54			
Day5-400 µg/mL-1	1039560	230.73	461.47	467.11	11.4	2.4
Day5-400 µg/mL-2	1035500	229.83	459.66			
Day5-400 µg/mL-3	1081740	240.10	480.19			
Day5-40 µg/mL-1	1055420	234.25	46.85	46.34	1.7	3.6
Day5-40 µg/mL-2	1001610	222.31	44.46			
Day5-40 µg/mL-3	1074680	238.53	47.71			

Day5-4 µg/mL-1	611900	135.81	5.43	5.41	0.1	1.6
Day5-4 µg/mL-2	617069	136.96	5.48			
Day5-4 µg/mL-3	598129	132.76	5.31			
Day7-STOCK SOLUTION-1	907547	202.82	20281.59	19896.75	346.5	1.7
Day7-STOCK SOLUTION-2	877474	196.10	19609.53			
Day7-STOCK SOLUTION-3	885958	197.99	19799.12			
Day7-4000 µg/mL-1	809976	181.01	3620.22	3749.60	113.9	3.0
Day7-4000 µg/mL-2	857996	191.74	3834.85			
Day7-4000 µg/mL-3	848799	189.69	3793.74			
Day7-400 µg/mL-1	1019150	227.76	455.51	465.17	12.3	2.6
Day7-400 µg/mL-2	1031360	230.49	460.97			
Day7-400 µg/mL-3	1071760	239.51	479.03			
Day7-40 µg/mL-1	1023090	228.64	45.73	47.17	1.4	3.0
Day7-40 µg/mL-2	1056690	236.15	47.23			
Day7-40 µg/mL-3	1086010	242.70	48.54			
Day7-4 µg/mL-1	608930	136.08	5.44	5.41	0.1	1.3
Day7-4 µg/mL-2	610196	136.36	5.45			
Day7-4 µg/mL-3	595640	133.11	5.32			
Day9-STOCK SOLUTION-1	901099	204.33	20432.63	20237.48	182.8	0.9
Day9-STOCK SOLUTION-2	885112	200.70	20070.12			
Day9-STOCK SOLUTION-3	891268	202.10	20209.71			
Day9-4000 µg/mL-1	851737	193.13	3862.67	3798.13	57.8	1.5
Day9-4000 µg/mL-2	827136	187.55	3751.10			
Day9-4000 µg/mL-3	833647	189.03	3780.63			
Day9-400 µg/mL-1	1033720	234.40	468.80	457.86	10.9	2.4
Day9-400 µg/mL-2	985522	223.47	446.94			
Day9-400 µg/mL-3	1009590	228.93	457.85			
Day9-40 µg/mL-1	1022330	231.82	46.36	46.76	0.7	1.5
Day9-40 µg/mL-2	1022130	231.77	46.35			
Day9-40 µg/mL-3	1048790	237.82	47.56			
Day9-4 µg/mL-1	605334	137.26	5.49	5.51	0.0	0.5
Day9-4 µg/mL-2	610640	138.46	5.54			
Day9-4 µg/mL-3	605086	137.20	5.49			
Day12-STOCK SOLUTION-1	877849	201.15	20114.61	20594.18	507.3	2.5
Day12-STOCK SOLUTION-2	921954	211.25	21125.21			
Day12-STOCK SOLUTION-3	896532	205.43	20542.70			
Day12-4000 µg/mL-1	822294	188.42	3768.33	3798.90	39.0	1.0

Day12-4000 µg/mL-2	838541	192.14	3842.79			
Day12-4000 µg/mL-3	826059	189.28	3785.58			
Day12-400 µg/mL-1	1032770	236.64	473.29	469.64	12.8	2.7
Day12-400 µg/mL-2	993679	227.69	455.37			
Day12-400 µg/mL-3	1048010	240.14	480.27			
Day12-40 µg/mL-1	1014680	232.50	46.50	46.26	0.7	1.4
Day12-40 µg/mL-2	993239	227.59	45.52			
Day12-40 µg/mL-3	1020590	233.85	46.77			
Day12-4 µg/mL-1	582631	133.50	5.34	5.43	0.1	1.4
Day12-4 µg/mL-2	599017	137.26	5.49			
Day12-4 µg/mL-3	594633	136.25	5.45			

Supplementary Table S17 Standard curve data for the active substance florfenicol (FLO). A calibration curve based on the concentrations and their corresponding peak areas can be used to determine the concentration of substances in the samples.

**STD concentration (ng/mL)**	**Peak area (Int unit)**	**Linearity (y=ax+b)**	
500	2338870	a (slope)	4674.38899
200	930729	b (intercept)	0
100	464376	r	0.99999
50	237605	r^2	0.99998
20	98689	Requirement: r ≥ 0.99	

STD - standard solutions concentration; a - slope; b - y-axis intercept; r - correlation coefficient; r2 - coefficient of determination

Supplementary Table S18 Repeatability test data for the active substance florfenicol (FLO) in the analytical assay. It provides information on the consistency of the replicates, which is important for assessing the reliability of analytical measurements. The low CV% value shown here is favorable because it indicates that the analytical process is stable and repeatable.

**Repeatability**		
Sample	Peak area	c (FF basis; STD) (ng/mL)
Repeat1	462344	98.91
Repeat2	468737	100.28
Repeat3	476349	101.91
Repeat4	457412	97.85
Repeat5	483913	103.52
Repeat6	477516	102.16
Requirement: CV% < 10	Mean	100.77
	SD	2.14
	CV%	2.13

SD - standard deviation; CV% - variance between replicates

Supplementary Table S19 Determination of florfenicol (FLO) limits. These data are key to assessing the sensitivity and accuracy of the analytical method. Low LOD (limit of detection) and LOQ (limit of quantitation) values indicate good sensitivity, but high CV% suggests that background noise shows considerable variability, which may affect the accuracy of determining very low concentrations.

LOD, LOQ				
Sample	Peak area	c (FLO basis; STD) (ng/mL)	LOD (ng/mL)	LOQ (ng/mL)
Noise1	809	0.17	0.43	1.42
Noise2	647	0.14		
Noise3	411	0.09		
Noise4	569	0.12		
Noise5	702	0.15		
Noise6	782	0.17		
Noise7	730	0.16		
Mean	664	0.14		
SD	137.98	0.03		
CV%	20.8	20.8		

SD - standard deviation; CV% - variance between replicates

Supplementary Table S20 System assay for the measurement of the active substance florfenicol (FLO). The CV% values are quite low, suggesting that the system is performing relatively consistently, the chromatography system is working properly and is suitable for analysis.

System suitability			
*t*0 (min) = 1.669			
	k'	N	Asymmetry factor
STD-500	2.866	9041	0.85
STD-200	2.856	10233	1.05
STD-100	2.865	9036	1.03
Mean	2.862	9436	0.98
SD	0.006	690	0.11
CV%	0.195	7.3	11.28
k'		> 2	
N		≥ 2000	
Asymmetry factor		≤ 2	

t0 – dead time; k' – capacity factor; N – theoretical number of plates; SD - standard deviation; CV% - variance between replicates

Supplementary Table S21 Analytical reliability of enrofloxacin (ENFX) samples. The standard deviation (Stdev) measures the deviation of individual measurements from the mean. Low values of the relative standard deviation (RSD%) indicate that measurements are consistent.

Sample	Peak area (counts)	c (ENFX basis; STD) (ng/ml)	c (ENFX basis; Sample) (µg/ml)	c (ENFX basis; Sample) mean (µg/ml)	STDEV	CV %
Day"0"-150µg/mL-1	2470100	78.352	156.704	156.39	0.9	0.6
Day"0"-150µg/mL-2	2448770	77.676	155.351			
Day"0"-150µg/mL-3	2476610	78.559	157.117			
Day"0"-15µg/mL-1	2866270	90.919	18.184	16.77	1.2	7.3
Day"0"-15µg/mL-2	2522180	80.004	16.001			
Day"0"-15µg/mL-3	2539660	80.559	16.112			
Day"0"-1.5µg/mL-1	1294000	41.046	2.052	2.07	0.0	0.9
Day"0"-1.5µg/mL-2	1317150	41.780	2.089			
Day"0"-1.5µg/mL-3	1308970	41.521	2.076			
Day"0"-0.15µg/mL-1	715454	22.694	0.227	0.23	0.0	1.4
Day"0"-0.15µg/mL-2	701076	22.238	0.222			
Day"0"-0.15µg/mL-3	719630	22.827	0.228			
Day"0"-0.015µg/mL-1	251888	7.990	0.024	0.02	0.0	1.5
Day"0"-0.015µg/mL-2	251889	7.990	0.024			
Day"0"-0.015µg/mL-3	245430	7.785	0.023			
Day1-150µg/mL-1	2560490	81.219	162.439	157.85	4.9	3.1
Day1-150µg/mL-2	2405480	76.302	152.605			
Day1-150µg/mL-3	2498510	79.253	158.507			
Day1-15µg/mL-1	2522160	80.004	16.001	16.55	0.5	2.9
Day1-15µg/mL-2	2643050	83.838	16.768			
Day1-15µg/mL-3	2662000	84.439	16.888			
Day1-1.5µg/mL-1	1197360	37.981	1.899	2.03	0.1	6.5
Day1-1.5µg/mL-2	1362980	43.234	2.162			
Day1-1.5µg/mL-3	1273860	40.407	2.020			
Day1-0.15µg/mL-1	699546	22.190	0.222	0.22	0.0	2.2
Day1-0.15µg/mL-2	669474	21.236	0.212			
Day1-0.15µg/mL-3	690435	21.901	0.219			
Day1-0.015µg/mL-1	247059	7.837	0.024	0.02	0.0	6.1
Day1-0.015µg/mL-2	219564	6.965	0.021			
Day1-0.015µg/mL-3	227997	7.232	0.022			
Day2-150µg/mL-1	2566660	81.415	162.830	158.51	5.0	3.1
Day2-150µg/mL-2	2412420	76.523	153.045			
Day2-150µg/mL-3	2516370	79.820	159.640			
Day2-15µg/mL-1	2681810	85.068	17.014	17.12	0.2	0.9
Day2-15µg/mL-2	2687670	85.254	17.051			
Day2-15µg/mL-3	2725730	86.461	17.292			
Day2-1.5µg/mL-1	1102240	34.963	1.748	1.93	0.2	8.1
Day2-1.5µg/mL-2	1287210	40.831	2.042			
Day2-1.5µg/mL-3	1255820	39.835	1.992			
Day2-0.15µg/mL-1	692214	21.957	0.220	0.21	0.0	4.0

Day2-0.15µg/mL-2	640278	20.310	0.203			
Day2-0.15µg/mL-3	676659	21.464	0.215			
Day2-0.015µg/mL-1	232053	7.361	0.022	0.02	0.0	2.0
Day2-0.015µg/mL-2	226584	7.187	0.022			
Day2-0.015µg/mL-3	223215	7.080	0.021			
Day5-150µg/mL-1	2201390	69.829	139.657	139.40	0.8	0.6
Day5-150µg/mL-2	2208300	70.048	140.096			
Day5-150µg/mL-3	2182500	69.229	138.459			
Day5-15µg/mL-1	2479560	78.652	15.730	16.07	0.5	3.0
Day5-15µg/mL-2	2620060	83.109	16.622			
Day5-15µg/mL-3	2498070	79.239	15.848			
Day5-1.5µg/mL-1	1069290	33.918	1.696	1.67	0.1	3.8
Day5-1.5µg/mL-2	1080260	34.266	1.713			
Day5-1.5µg/mL-3	1006440	31.925	1.596			
Day5-0.15µg/mL-1	630458	19.998	0.200	0.19	0.0	6.3
Day5-0.15µg/mL-2	581511	18.446	0.184			
Day5-0.15µg/mL-3	558032	17.701	0.177			
Day5-0.015µg/mL-1	218828	6.941	0.021	0.02	0.0	6.3
Day5-0.015µg/mL-2	209970	6.660	0.020			
Day5-0.015µg/mL-3	192953	6.121	0.018			
Day7-150µg/mL-1	2371750	75.233	150.465	149.41	2.4	1.6
Day7-150µg/mL-2	2311300	73.315	146.630			
Day7-150µg/mL-3	2382370	75.569	151.139			
Day7-15µg/mL-1	2642560	83.823	16.765	16.78	0.1	0.9
Day7-15µg/mL-2	2669090	84.664	16.933			
Day7-15µg/mL-3	2623140	83.207	16.641			
Day7-1.5µg/mL-1	1141940	36.223	1.811	1.80	0.0	1.0
Day7-1.5µg/mL-2	1134050	35.972	1.799			
Day7-1.5µg/mL-3	1119640	35.515	1.776			
Day7-0.15µg/mL-1	598806	18.994	0.190	0.20	0.0	2.5
Day7-0.15µg/mL-2	622356	19.741	0.197			
Day7-0.15µg/mL-3	628417	19.934	0.199			
Day7-0.015µg/mL-1	223157	7.079	0.021	0.02	0.0	5.5
Day7-0.015µg/mL-2	200355	6.355	0.019			
Day7-0.015µg/mL-3	215824	6.846	0.021			
Day9-150µg/mL-1	2326840	73.808	147.616	141.13	5.6	4.0
Day9-150µg/mL-2	2176680	69.045	138.090			
Day9-150µg/mL-3	2170120	68.837	137.674			
Day9-15µg/mL-1	2519360	79.915	15.983	15.99	0.1	0.8
Day9-15µg/mL-2	2501480	79.348	15.870			
Day9-15µg/mL-3	2541920	80.630	16.126			
Day9-1.5µg/mL-1	1063140	33.723	1.686	1.63	0.1	5.1
Day9-1.5µg/mL-2	1055300	33.474	1.674			
Day9-1.5µg/mL-3	967856	30.701	1.535			
Day9-0.15µg/mL-1	599160	19.006	0.190	0.19	0.0	2.3
Day9-0.15µg/mL-2	615740	19.531	0.195			

Day9-0.15µg/mL-3	627374	19.900	0.199			
Day9-0.015µg/mL-1	215251	6.828	0.020	0.02	0.0	5.7
Day9-0.015µg/mL-2	203347	6.450	0.019			
Day9-0.015µg/mL-3	227726	7.224	0.022			
Day12-150µg/mL-1	2162620	68.599	137.198	139.00	2.8	2.0
Day12-150µg/mL-2	2242230	71.124	142.248			
Day12-150µg/mL-3	2168040	68.771	137.542			
Day12-15µg/mL-1	2598330	82.420	16.484	16.08	0.4	2.2
Day12-15µg/mL-2	2508620	79.574	15.915			
Day12-15µg/mL-3	2495440	79.156	15.831			
Day12-1.5µg/mL-1	1059290	33.601	1.680	1.68	0.0	2.1
Day12-1.5µg/mL-2	1076220	34.138	1.707			
Day12-1.5µg/mL-3	1032860	32.763	1.638			
Day12-0.15µg/mL-1	615567	19.526	0.195	0.19	0.0	0.7
Day12-0.15µg/mL-2	608146	19.291	0.193			
Day12-0.15µg/mL-3	609086	19.320	0.193			
Day12-0.015µg/mL-1	214514	6.804	0.020	0.02	0.0	4.0
Day12-0.015µg/mL-2	206059	6.536	0.020			
Day12-0.015µg/mL-3	198067	6.283	0.019			

Supplementary Table S22 Standard curve data for the active substance enrofloxacin (ENFX). A calibration curve based on the concentrations and their corresponding peak areas can be used to determine the concentration of substances in the samples.

**STD concentration (ng/mL)**	**Peak area (Int unit)**	**Linearity (y=ax+b)**	
100	3153500	a (slope)	31525.6
50	1573070	b (intercept)	0
20	626997	r	0.99999
10	327032	r^2	0.99998
5	161488	Requirement: r ≥ 0.99	

STD - standard solutions concentration; a - slope; b - y-axis intercept; r - correlation coefficient; r2 - coefficient of determination

Supplementary Table S23 Repeatability test data for the active substance enrofloxacin (ENFX) in the analytical assay. It provides information on the consistency of the replicates, which is important for assessing the reliability of analytical measurements. The low CV% value shown here is favorable because it indicates that the analytical process is stable and repeatable.

**Repeatability**		
Sample	Peak area	c (ENFX basis; STD) (ng/mL)
Repeat1	628696	19.94
Repeat2	645702	20.48
Repeat3	623084	19.76
Repeat4	632528	20.06
Repeat5	638211	20.24
Repeat6	637747	20.23
Requirement: CV% < 10	Mean	20.12
	SD	0.25
	CV%	1.26

SD - standard deviation; CV% - variance between replicates

Supplementary Table S24 Determination of enrofloxacin (ENFX) limits. These data are key to assessing the sensitivity and accuracy of the analytical method. Low LOD (limit of detection) and LOQ (limit of quantitation) values indicate good sensitivity, but high CV% suggests that background noise shows considerable variability, which may affect the accuracy of determining very low concentrations.

**LOD, LOQ**				
Sample	Peak area	c (ENFX basis; STD) (ng/mL)	LOD (ng/mL)	LOQ (ng/mL)
Noise1	8715	0.28	1.09	3.63
Noise2	12272	0.39		
Noise3	9946	0.32		
Noise4	10258	0.33		
Noise5	14092	0.45		
Noise6	11855	0.38		
Noise7	12953	0.41		
Mean	11442	0.36		
SD	1881.44	0.06		
CV%	16.4	16.4		

SD - standard deviation; CV% - variance between replicates

Supplementary Table S25 System assay for the measurement of the active substance enrofloxacin (ENFX). The CV% values are quite low, suggesting that the system is performing relatively consistently, the chromatography system is working properly and is suitable for analysis.

**System suitability**			
*t*0 (min) = 0.556			
	k'	N	Asymmetry factor
STD-100	2.242	3932	1.76
STD-50	2.242	3821	1.73
STD-20	2.244	3517	1.74
Mean	2.243	3757	1.74
SD	0.001	215	0.02
CV%	0.045	5.7	0.88
k'		> 2	
N		≥ 2000	
Asymmetry factor		≤ 2	

t0 – dead time; k' – capacity factor; N – theoretical number of plates; SD - standard deviation; CV% - variance between replicates

Supplementary Table S26 Analytical reliability of colistin (COL) samples. The standard deviation (Stdev) measures the deviation of individual measurements from the mean. Low values of the relative standard deviation (RSD%) indicate that measurements are consistent.

**Sample**	**Peak area (counts)**	**c (COL basis; STD) (ng/ml)**	**c (COL basis; Sample) (µg/ml)**	**c (COL basis; Sample) mean (µg/ml)**	**STDEV**	**CV%**
Day0-Stock sol.-1	931718	110.54	2763.62	2918.08	139.9	4.8
Day0-Stock sol.-2	996033	118.18	2954.39			
Day0-Stock sol.-3	1023620	121.45	3036.22			
Day0-250 µg/mL-1	1957690	232.27	232.27	242.96	12.1	5.0
Day0-250 µg/mL-2	2158560	256.11	256.11			
Day0-250 µg/mL-3	2026940	240.49	240.49			
Day0-25 µg/mL-1	908264	107.76	26.94	27.55	1.0	3.5
Day0-25 µg/mL-2	966065	114.62	28.66			
Day0-25 µg/mL-3	912522	108.27	27.07			
Day0-2.5 µg/mL-1	505184	59.94	3.00	3.01	0.1	2.9
Day0-2.5 µg/mL-2	522896	62.04	3.10			
Day0-2.5 µg/mL-3	493708	58.58	2.93			
Day0-0.25 µg/mL-1	327219	38.82	0.39	0.39	0.0	1.0
Day0-0.25 µg/mL-2	331071	39.28	0.39			
Day0-0.25 µg/mL-3	324810	38.54	0.39			
Day1-Stock sol.-1	903690	103.23	2580.66	2588.22	71.8	2.8
Day1-Stock sol.-2	882618	100.82	2520.49			
Day1-Stock sol.-3	932704	106.54	2663.52			
Day1-250 µg/mL-1	1636360	186.92	186.92	184.25	4.2	2.3
Day1-250 µg/mL-2	1632340	186.46	186.46			
Day1-250 µg/mL-3	1570440	179.39	179.39			

Day1-25 µg/mL-1	721252	82.39	20.60			
Day1-25 µg/mL-2	730888	83.49	20.87	20.59	0.3	1.4
Day1-25 µg/mL-3	710489	81.16	20.29			
Day1-2.5 µg/mL-1	274262	31.33	1.57			
Day1-2.5 µg/mL-2	261935	29.92	1.50	1.52	0.0	2.9
Day1-2.5 µg/mL-3	260195	29.72	1.49			
Day1-0.25 µg/mL-1	231122	26.40	0.26			
Day1-0.25 µg/mL-2	216878	24.77	0.25	0.26	0.0	3.7
Day1-0.25 µg/mL-3	231388	26.43	0.26			
Day2-Stock sol.-1	783028	85.94	2148.50			
Day2-Stock sol.-2	748524	82.15	2053.83	2100.04	47.4	2.3
Day2-Stock sol.-3	764548	83.91	2097.80			
Day2-250 µg/mL-1	1328100	145.76	145.76			
Day2-250 µg/mL-2	1246920	136.85	136.85	139.44	5.5	4.0
Day2-250 µg/mL-3	1236310	135.69	135.69			
Day2-25 µg/mL-1	566890	62.22	15.55			
Day2-25 µg/mL-2	515906	56.62	14.16	14.73	0.7	5.0
Day2-25 µg/mL-3	527951	57.94	14.49			
Day2-2.5 µg/mL-1	240410	26.39	1.32			
Day2-2.5 µg/mL-2	216551	23.77	1.19	1.22	0.1	7.5
Day2-2.5 µg/mL-3	208235	22.85	1.14			
Day2-0.25 µg/mL-1	154649	16.97	0.17			
Day2-0.25 µg/mL-2	160683	17.64	0.18	0.17	0.0	2.6
Day2-0.25 µg/mL-3	152962	16.79	0.17			
Day5-Stock sol.-1	699913	76.85	1921.27			
Day5-Stock sol.-2	710057	77.96	1949.11	1946.33	23.8	1.2
Day5-Stock sol.-3	717157	78.74	1968.60			
Day5-250 µg/mL-1	1138850	125.05	125.05			
Day5-250 µg/mL-2	1226170	134.63	134.63	127.08	6.8	5.3
Day5-250 µg/mL-3	1107220	121.57	121.57			
Day5-25 µg/mL-1	489128	53.71	13.43			
Day5-25 µg/mL-2	495087	54.36	13.59	13.67	0.3	2.2
Day5-25 µg/mL-3	510200	56.02	14.01			
Day5-2.5 µg/mL-1	183769	20.18	1.01			
Day5-2.5 µg/mL-2	164949	18.11	0.91	0.93	0.1	7.2
Day5-2.5 µg/mL-3	161032	17.68	0.88			
Day5-0.25 µg/mL-1	118798	13.04	0.13			
Day5-0.25 µg/mL-2	115870	12.72	0.13	0.13	0.0	2.1
Day5-0.25 µg/mL-3	120823	13.27	0.13			
Day7-Stock sol.-1	662653	73.07	1826.68			
Day7-Stock sol.-2	643057	70.91	1772.66	1797.89	27.2	1.5
Day7-Stock sol.-3	650916	71.77	1794.33			
Day7-250 µg/mL-1	1043160	115.02	115.02			
Day7-250 µg/mL-2	1121180	123.63	123.63	117.71	5.1	4.4
Day7-250 µg/mL-3	1038300	114.49	114.49			
Day7-25 µg/mL-1	349674	38.56	9.64	10.25	0.7	6.6

Day7-25 µg/mL-2	398311	43.92	10.98			
Day7-25 µg/mL-3	367186	40.49	10.12			
Day7-2.5 µg/mL-1	165854	18.29	0.91			
Day7-2.5 µg/mL-2	149299	16.46	0.82	0.87	0.0	5.3
Day7-2.5 µg/mL-3	157463	17.36	0.87			
Day7-0.25 µg/mL-1	105407	11.62	0.12			
Day7-0.25 µg/mL-2	108887	12.01	0.12	0.12	0.0	2.2
Day7-0.25 µg/mL-3	109941	12.12	0.12			
Day9-Stock sol.-1	593038	65.33	1633.36			
Day9-Stock sol.-2	527478	58.11	1452.79	1513.99	103.4	6.8
Day9-Stock sol.-3	528571	58.23	1455.80			
Day9-250 µg/mL-1	910575	100.32	100.32			
Day9-250 µg/mL-2	1005700	110.80	110.80	107.65	6.4	5.9
Day9-250 µg/mL-3	1015100	111.83	111.83			
Day9-25 µg/mL-1	347926	38.33	9.58			
Day9-25 µg/mL-2	324810	35.78	8.95	9.11	0.4	4.6
Day9-25 µg/mL-3	319282	35.18	8.79			
Day9-2.5 µg/mL-1	117787	12.98	0.65			
Day9-2.5 µg/mL-2	123504	13.61	0.68	0.66	0.0	3.3
Day9-2.5 µg/mL-3	115972	12.78	0.64			
Day9-0.25 µg/mL-1	92139	10.15	0.10			
Day9-0.25 µg/mL-2	95795	10.55	0.11	0.11	0.0	5.4
Day9-0.25 µg/mL-3	102513	11.29	0.11			
Day12-Stock sol.-1	452938	44.71	1117.74			
Day12-Stock sol.-2	486124	47.99	1199.63	1146.45	46.1	4.0
Day12-Stock sol.-3	454656	44.88	1121.98			
Day12-250 µg/mL-1	787750	77.76	77.76			
Day12-250 µg/mL-2	909506	89.78	89.78	82.78	6.2	7.5
Day12-250 µg/mL-3	818652	80.81	80.81			
Day12-25 µg/mL-1	254283	25.10	6.28			
Day12-25 µg/mL-2	305968	30.20	7.55	6.75	0.7	10.3
Day12-25 µg/mL-3	260195	25.68	6.42			
Day12-2.5 µg/mL-1	94261	9.30	0.47			
Day12-2.5 µg/mL-2	99725	9.84	0.49	0.49	0.0	4.7
Day12-2.5 µg/mL-3	103534	10.22	0.51			
Day12-0.25 µg/mL-1	78394	7.74	0.08			
Day12-0.25 µg/mL-2	90172	8.90	0.09	0.08	0.0	7.1
Day12-0.25 µg/mL-3	86343	8.52	0.09			

Supplementary Table S27 Standard curve data for the active substance colistin (COL). A calibration curve based on the concentrations and their corresponding peak areas can be used to determine the concentration of substances in the samples.

**STD concentration (ng/mL)**	**Peak area (Int unit)**	**Linearity (y=ax+b)**	
500	4218210	a (slope)	8428.41446
200	1676010	b (intercept)	0
100	842579	r	0.99999
50	420256	r^2	0.99999
20	169452	Requirement: r ≥ 0.99	

STD - standard solutions concentration; a - slope; b - y-axis intercept; r - correlation coefficient; r2 - coefficient of determination

Supplementary Table S28 Repeatability test data for the active substance colistin (COL) in the analytical assay. It provides information on the consistency of the replicates, which is important for assessing the reliability of analytical measurements. The low CV% value shown here is favorable because it indicates that the analytical process is stable and repeatable.

**Repeatability**		
Sample	Area	c (COL base; STD) (ng/mL)
Repeat1	343762	40.79
Repeat2	376387	44.66
Repeat3	353210	41.91
Repeat4	342846	40.68
Repeat5	367445	43.60
Repeat6	354463	42.06
Requirement: CV% < 10	Mean	42.28
	SD	1.57
	CV%	3.72

SD - standard deviation; CV% - variance between replicates

Supplementary Table S29 Determination of colistin (COL) limits. These data are key to assessing the sensitivity and accuracy of the analytical method. Low LOD (limit of detection) and LOQ (limit of quantitation) values indicate good sensitivity, but high CV% suggests that background noise shows considerable variability, which may affect the accuracy of determining very low concentrations.

LOD, LOQ				
Sample	Area	c (COL base; STD) (ng/mL)	LOD (ng/mL)	LOQ (ng/mL)
Noise1	872	0.04	0.12	0.39
Noise2	794	0.04		
Noise3	485	0.02		
Noise4	1025	0.05		
Noise5	475	0.02		
Noise6	975	0.05		
Noise7	771	0.04		
Mean	771	0.04		
SD	218.38	0.01		
CV%	28.3	28.3		

SD - standard deviation; CV% - variance between replicates

Supplementary Table S30 System assay for the measurement of the active substance colistin (COL). The CV% values are quite low, suggesting that the system is performing relatively consistently, the chromatography system is working properly and is suitable for analysis.

System suitability			
*t*0 (min) = 0.729			
	k'	N	Asymmetry factor
STD-500	2.290	4986	1.73
STD-200	2.285	3806	1.72
STD-100	2.290	3817	1.84
Mean	2.288	4203	1.76
SD	0.003	678	0.07
CV%	0.119	16.1	3.78
k'	> 2		
N	≥ 2000		
Asymmetry factor	≤ 2		

t0 – dead time; k' – capacity factor; N – theoretical number of plates; SD - standard deviation; CV% - variance between replicates

Supplementary Table S31 Analytical reliability of neomycin (NEO) samples. The standard deviation (Stdev) measures the deviation of individual measurements from the mean. Low values of the relative standard deviation (RSD%) indicate that measurements are consistent.

Sample	Peak area (counts)	*c* (NEO basis; STD) (ng/mL)	*c* (NEO basis; Sample) (µg/mL)	*c* (NEO basis; Sample) mean (µg/mL)	STDEV	CV%
Day0-STOCK SOLUTION-1	2083020	432.13	43213.44	43472.35	656.8	1.5
Day0-STOCK SOLUTION-2	2071980	429.84	42984.41			
Day0-STOCK SOLUTION-3	2131500	442.19	44219.19			
Day0-4000 µg/mL-1	2057950	426.93	4269.34	4354.81	79.5	1.8
Day0-4000 µg/mL-2	2105770	436.85	4368.54			
Day0-4000 µg/mL-3	2133730	442.65	4426.55			
Day0-400 µg/mL-1	1980560	410.88	410.88	409.30	2.9	0.7
Day0-400 µg/mL-2	1956740	405.94	405.94			
Day0-400 µg/mL-3	1981520	411.08	411.08			
Day0-40 µg/mL-1	1747890	362.61	36.26	35.69	0.5	1.5
Day0-40 µg/mL-2	1696670	351.98	35.20			
Day0-40 µg/mL-3	1716020	356.00	35.60			
Day0-4 µg/mL-1	254046	52.70	2.11	2.20	0.1	4.0
Day0-4 µg/mL-2	274825	57.01	2.28			
Day0-4 µg/mL-3	267284	55.45	2.22			
Day1-STOCK SOLUTION-1	1955650	393.27	39327.44	38474.39	838.6	2.2
Day1-STOCK SOLUTION-2	1872290	376.51	37651.10			
Day1-STOCK SOLUTION-3	1911750	384.45	38444.63			
Day1-4000 µg/mL-1	1686760	339.20	3392.02	3402.43	73.6	2.2
Day1-4000 µg/mL-2	1730850	348.07	3480.68			
Day1-4000 µg/mL-3	1658200	333.46	3334.58			
Day1-400 µg/mL-1	1480650	297.75	297.75	300.56	10.5	3.5
Day1-400 µg/mL-2	1451000	291.79	291.79			
Day1-400 µg/mL-3	1552140	312.13	312.13			
Day1-40 µg/mL-1	1152040	231.67	23.17	22.74	0.4	1.9
Day1-40 µg/mL-2	1109600	223.14	22.31			
Day1-40 µg/mL-3	1130040	227.25	22.72			
Day1-4 µg/mL-1	210484	42.33	1.69	1.62	0.1	8.8
Day1-4 µg/mL-2	181668	36.53	1.46			
Day1-4 µg/mL-3	213832	43.00	1.72			
Day2-STOCK SOLUTION-1	2160720	472.60	47259.89	47186.18	159.9	0.3
Day2-STOCK SOLUTION-2	2162370	472.96	47295.98			
Day2-STOCK SOLUTION-3	2148960	470.03	47002.67			

Day2-4000 µg/mL-1	1767960	386.69	3866.93	3801.23	75.4	2.0
Day2-4000 µg/mL-2	1745510	381.78	3817.83			
Day2-4000 µg/mL-3	1700290	371.89	3718.92			
Day2-400 µg/mL-1	1324550	289.71	289.71	294.41	5.9	2.0
Day2-400 µg/mL-2	1376250	301.02	301.02			
Day2-400 µg/mL-3	1337290	292.50	292.50			
Day2-40 µg/mL-1	1030930	225.49	22.55	21.67	0.9	4.0
Day2-40 µg/mL-2	989132	216.35	21.63			
Day2-40 µg/mL-3	952169	208.26	20.83			
Day2-4 µg/mL-1	60233	13.17	0.53	0.49	0.0	8.3
Day2-4 µg/mL-2	51016	11.16	0.45			
Day2-4 µg/mL-3	55357	12.11	0.48			
Day5-STOCK SOLUTION-1	1790660	446.88	44687.54	43507.29	1050.5	2.4
Day5-STOCK SOLUTION-2	1729430	431.59	43159.49			
Day5-STOCK SOLUTION-3	1710010	426.75	42674.84			
Day5-4000 µg/mL-1	1358360	338.99	3389.91	3491.53	95.4	2.7
Day5-4000 µg/mL-2	1434200	357.92	3579.18			
Day5-4000 µg/mL-3	1404680	350.55	3505.51			
Day5-400 µg/mL-1	941769	235.03	235.03	226.67	8.7	3.8
Day5-400 µg/mL-2	872343	217.70	217.70			
Day5-400 µg/mL-3	910738	227.28	227.28			
Day5-40 µg/mL-1	670599	167.35	16.74	17.23	0.6	3.3
Day5-40 µg/mL-2	715636	178.59	17.86			
Day5-40 µg/mL-3	684795	170.90	17.09			
Day5-4 µg/mL-1	40254	10.05	0.40	0.42	0.0	4.1
Day5-4 µg/mL-2	42393	10.58	0.42			
Day5-4 µg/mL-3	43634	10.89	0.44			
Day7-STOCK SOLUTION-1	1546850	405.82	40581.67	39223.66	1360.8	3.5
Day7-STOCK SOLUTION-2	1443110	378.60	37860.05			
Day7-STOCK SOLUTION-3	1495300	392.29	39229.26			
Day7-4000 µg/mL-1	1038430	272.43	2724.33	2729.54	26.7	1.0
Day7-4000 µg/mL-2	1051440	275.85	2758.46			
Day7-4000 µg/mL-3	1031380	270.58	2705.83			
Day7-400 µg/mL-1	695792	182.54	182.54	178.39	3.6	2.0
Day7-400 µg/mL-2	671742	176.23	176.23			
Day7-400 µg/mL-3	672351	176.39	176.39			
Day7-40 µg/mL-1	443919	116.46	11.65	11.80	0.3	2.2
Day7-40 µg/mL-2	461261	121.01	12.10			
Day7-40 µg/mL-3	444316	116.57	11.66			
Day7-4 µg/mL-1	44797	11.75	0.47	0.50	0.0	4.7
Day7-4 µg/mL-2	49130	12.89	0.52			

Day7-4 µg/mL-3	47982	12.59	0.50			
Day9-STOCK SOLUTION-1	1419000	432.70	43270.20	42543.95	680.6	1.6
Day9-STOCK SOLUTION-2	1391800	424.41	42440.78			
Day9-STOCK SOLUTION-3	1374750	419.21	41920.87			
Day9-4000 µg/mL-1	868168	264.73	2647.34	2633.33	12.5	0.5
Day9-4000 µg/mL-2	862239	262.93	2629.26			
Day9-4000 µg/mL-3	860309	262.34	2623.38			
Day9-400 µg/mL-1	560067	170.78	170.78	173.56	2.8	1.6
Day9-400 µg/mL-2	578161	176.30	176.30			
Day9-400 µg/mL-3	569321	173.61	173.61			
Day9-40 µg/mL-1	411552	125.50	12.55	12.40	0.1	1.0
Day9-40 µg/mL-2	404014	123.20	12.32			
Day9-40 µg/mL-3	404332	123.29	12.33			
Day9-4 µg/mL-1	33210	10.13	0.41	0.40	0.0	2.1
Day9-4 µg/mL-2	31897	9.73	0.39			
Day9-4 µg/mL-3	32928	10.04	0.40			
Day12-STOCK SOLUTION-1	1426460	405.39	40538.61	40552.25	81.6	0.2
Day12-STOCK SOLUTION-2	1424340	404.78	40478.36			
Day12-STOCK SOLUTION-3	1430020	406.40	40639.78			
Day12-4000 µg/mL-1	798879	227.03	2270.34	2289.46	22.5	1.0
Day12-4000 µg/mL-2	803606	228.38	2283.77			
Day12-4000 µg/mL-3	814343	231.43	2314.28			
Day12-400 µg/mL-1	458260	130.23	130.23	127.36	2.7	2.1
Day12-400 µg/mL-2	439098	124.79	124.79			
Day12-400 µg/mL-3	447117	127.07	127.07			
Day12-40 µg/mL-1	272957	77.57	7.76	7.75	0.3	4.3
Day12-40 µg/mL-2	260768	74.11	7.41			
Day12-40 µg/mL-3	284461	80.84	8.08			
Day12-4 µg/mL-1	22998	6.54	0.26	0.26	0.0	2.2
Day12-4 µg/mL-2	22353	6.35	0.25			
Day12-4 µg/mL-3	22019	6.26	0.25			

Supplementary Table S32 Standard curve data for the active substance neomycin (NEO). A calibration curve based on the concentrations and their corresponding peak areas can be used to determine the concentration of substances in the samples.

**STD concentration (ng/mL)**	**Peak area (Int unit)**	**Linearity (y=ax+b)**	
1000	4786250	a (slope)	4820.30405
500	2451550	b (intercept)	0
200	1017210	r	0.99989
100	503673	r^2	0.99979
50	252271	Requirement: r ≥ 0.99	

STD - standard solutions concentration; a - slope; b - y-axis intercept; r - correlation coefficient; r2 - coefficient of determination

Supplementary Table S33 Repeatability test data for the active substance neomycin (NEO) in the analytical assay. It provides information on the consistency of the replicates, which is important for assessing the reliability of analytical measurements. The low CV% value shown here is favorable because it indicates that the analytical process is stable and repeatable.

**Repeatability**		
Sample	Area	c (NEO base; STD) (ng/mL)
Repeat1	1078670	223.78
Repeat2	989835	205.35
Repeat3	1048860	217.59
Repeat4	1063130	220.55
Repeat5	1097100	227.60
Repeat6	1017520	211.09
Requirement: CV% < 10	Mean	217.66
	SD	8.24
	CV%	3.78

SD - standard deviation; CV% - variance between replicates

Supplementary Table S34 Determination of neomycin (NEO) limits. These data are key to assessing the sensitivity and accuracy of the analytical method. Low LOD (limit of detection) and LOQ (limit of quantitation) values indicate good sensitivity, but high CV% suggests that background noise shows considerable variability, which may affect the accuracy of determining very low concentrations.

**LOD, LOQ**				
Sample	Area	c (NEO base; STD) (ng/mL)	LOD (ng/mL)	LOQ (ng/mL)
Noise1	2617	0.54	1.49	4.98
Noise2	2322	0.48		
Noise3	2766	0.57		
Noise4	2384	0.49		
Noise5	2440	0.51		
Noise6	2042	0.42		
Noise7	2234	0.46		
Mean	2401	0.50		
SD	239.72	0.05		
CV%	10.0	10.0		

SD - standard deviation; CV% - variance between replicates

Supplementary Table S35 System assay for the measurement of the active substance neomycin (NEO). The CV% values are quite low, suggesting that the system is performing relatively consistently, the chromatography system is working properly and is suitable for analysis.

**System suitability**			
*t*0 (min) = 0.620			
	k'	N	Asymmetry factor
STD-1000	10.610	28729	1.78
STD-500	10.600	28681	1.79
STD-200	10.618	28769	1.82
Mean	10.609	28727	1.80
SD	0.009	44	0.02
CV%	0.084	0.2	1.16
k'		> 2	
N		≥ 2000	
Asymmetry factor		≤ 2	

t0 – dead time; k' – capacity factor; N – theoretical number of plates; SD - standard deviation; CV% - variance between replicates

Supplementary Table S36 Analytical reliability of trimetoprim (TMP) samples. The standard deviation (Stdev) measures the deviation of individual measurements from the mean. Low values of the relative standard deviation (RSD%) indicate that measurements are consistent.

Sample	Peak area (counts)	c (TMP basis; STD) (ng/mL)	c (TMP basis; Sample) (µg/mL)	c (TMP basis; Sample) mean (µg/mL)	Stdev	CV%
Day0-STOCK SOLUTION-1	4587060	46.21	924.19	910.93	14.13	1.6
Day0-STOCK SOLUTION-2	4447470	44.80	896.07			
Day0-STOCK SOLUTION-3	4529100	45.63	912.52			
Day0-2000 µg/mL-1	4856800	48.93	97.85	99.42	3.87	3.9
Day0-2000 µg/mL-2	4793650	48.29	96.58			
Day0-2000 µg/mL-3	5153180	51.91	103.83			
Day0-200 µg/mL-1	5337150	53.77	10.75	10.93	0.18	1.7
Day0-200 µg/mL-2	5413630	54.54	10.91			
Day0-200 µg/mL-3	5519970	55.61	11.12			
Day0-20 µg/mL-1	2393660	24.11	1.21	1.23	0.02	1.7
Day0-20 µg/mL-2	2437240	24.55	1.23			
Day0-20 µg/mL-3	2474760	24.93	1.25			
Day0-2 µg/mL-1	577652	5.82	0.12	0.12	0.01	4.1
Day0-2 µg/mL-2	596937	6.01	0.12			
Day0-2 µg/mL-3	627025	6.32	0.13			
Day1-STOCK SOLUTION-1	4562300	46.68	933.58	929.84	3.27	0.4
Day1-STOCK SOLUTION-2	4532610	46.38	927.50			
Day1-STOCK SOLUTION-3	4537150	46.42	928.43			
Day1-2000 µg/mL-1	4741370	48.51	97.02	94.20	4.23	4.5
Day1-2000 µg/mL-2	4702840	48.12	96.23			
Day1-2000 µg/mL-3	4365810	44.67	89.34			
Day1-200 µg/mL-1	5293580	54.16	10.83	11.20	0.35	3.1
Day1-200 µg/mL-2	5488380	56.15	11.23			
Day1-200 µg/mL-3	5636920	57.67	11.53			
Day1-20 µg/mL-1	2465050	25.22	1.26	1.27	0.01	1.1
Day1-20 µg/mL-2	2514970	25.73	1.29			
Day1-20 µg/mL-3	2475490	25.33	1.27			
Day1-2 µg/mL-1	588695	6.02	0.12	0.13	0.01	5.8
Day1-2 µg/mL-2	606542	6.21	0.12			
Day1-2 µg/mL-3	657870	6.73	0.13			
Day2-STOCK SOLUTION-1	4521210	46.82	936.38	927.82	7.48	0.8
Day2-STOCK SOLUTION-2	4463830	46.22	924.50			

Day2-STOCK SOLUTION-3	4454490	46.13	922.56			
Day2-2000 μg/mL-1	4771740	49.41	98.83	98.26	1.52	1.6
Day2-2000 μg/mL-2	4800400	49.71	99.42			
Day2-2000 μg/mL-3	4661130	48.27	96.54			
Day2-200 μg/mL-1	5209870	53.95	10.79	11.07	0.33	3.0
Day2-200 μg/mL-2	5302960	54.91	10.98			
Day2-200 μg/mL-3	5523750	57.20	11.44			
Day2-20 μg/mL-1	2304240	23.86	1.19	1.21	0.06	4.7
Day2-20 μg/mL-2	2455750	25.43	1.27			
Day2-20 μg/mL-3	2244960	23.25	1.16			
Day2-2 μg/mL-1	607020	6.29	0.13	0.13	0.01	8.1
Day2-2 μg/mL-2	597890	6.19	0.12			
Day2-2 μg/mL-3	690869	7.15	0.14			
Day5-STOCK SOLUTION-1	3512080	46.06	921.14	920.98	4.35	0.5
Day5-STOCK SOLUTION-2	3494590	45.83	916.55			
Day5-STOCK SOLUTION-3	3527760	46.26	925.25			
Day5-2000 μg/mL-1	3581350	46.97	93.93	95.68	2.39	2.5
Day5-2000 μg/mL-2	3751760	49.20	98.40			
Day5-2000 μg/mL-3	3610610	47.35	94.70			
Day5-200 μg/mL-1	4129240	54.15	10.83	10.68	0.15	1.4
Day5-200 μg/mL-2	4068630	53.36	10.67			
Day5-200 μg/mL-3	4016870	52.68	10.54			
Day5-20 μg/mL-1	1764030	23.13	1.16	1.16	0.03	2.5
Day5-20 μg/mL-2	1809230	23.73	1.19			
Day5-20 μg/mL-3	1719970	22.56	1.13			
Day5-2 μg/mL-1	421036	5.52	0.11	0.11	0.00	0.6
Day5-2 μg/mL-2	425161	5.58	0.11			
Day5-2 μg/mL-3	425058	5.57	0.11			
Day7-STOCK SOLUTION-1	3640300	47.82	956.34	948.26	7.15	0.8
Day7-STOCK SOLUTION-2	3588580	47.14	942.75			
Day7-STOCK SOLUTION-3	3599820	47.29	945.70			
Day7-2000 μg/mL-1	3611540	47.44	94.88	94.17	1.01	1.1
Day7-2000 μg/mL-2	3601650	47.31	94.62			
Day7-2000 μg/mL-3	3540340	46.50	93.01			
Day7-200 μg/mL-1	4076760	53.55	10.71	10.65	0.10	0.9
Day7-200 μg/mL-2	4075490	53.53	10.71			
Day7-200 μg/mL-3	4011780	52.70	10.54			
Day7-20 μg/mL-1	1748870	22.97	1.15	1.12	0.03	2.6
Day7-20 μg/mL-2	1692820	22.24	1.11			
Day7-20 μg/mL-3	1663100	21.85	1.09			

Day7-2 µg/mL-1	396788	5.21	0.10	0.11	0.00	2.9
Day7-2 µg/mL-2	417523	5.48	0.11			
Day7-2 µg/mL-3	398190	5.23	0.10			
Day9-STOCK SOLUTION-1	3575400	47.44	948.72	948.02	7.32	0.8
Day9-STOCK SOLUTION-2	3598960	47.75	954.97			
Day9-STOCK SOLUTION-3	3543940	47.02	940.37			
Day9-2000 µg/mL-1	3509680	46.56	93.13	93.02	0.70	0.7
Day9-2000 µg/mL-2	3477720	46.14	92.28			
Day9-2000 µg/mL-3	3529730	46.83	93.66			
Day9-200 µg/mL-1	3945090	52.34	10.47	10.47	0.14	1.4
Day9-200 µg/mL-2	3999970	53.07	10.61			
Day9-200 µg/mL-3	3890920	51.62	10.32			
Day9-20 µg/mL-1	1698590	22.54	1.13	1.16	0.03	2.8
Day9-20 µg/mL-2	1748270	23.19	1.16			
Day9-20 µg/mL-3	1796140	23.83	1.19			
Day9-2 µg/mL-1	387534	5.14	0.10	0.10	0.00	1.6
Day9-2 µg/mL-2	388172	5.15	0.10			
Day9-2 µg/mL-3	377105	5.00	0.10			
Day12-STOCK SOLUTION-1	2668740	42.61	852.23	876.10	26.01	3.0
Day12-STOCK SOLUTION-2	2830300	45.19	903.83			
Day12-STOCK SOLUTION-3	2731340	43.61	872.22			
Day12-2000 µg/mL-1	2785820	44.48	88.96	89.66	1.67	1.9
Day12-2000 µg/mL-2	2769820	44.23	88.45			
Day12-2000 µg/mL-3	2867260	45.78	91.56			
Day12-200 µg/mL-1	3358810	53.63	10.73	10.15	0.52	5.1
Day12-200 µg/mL-2	3044330	48.61	9.72			
Day12-200 µg/mL-3	3131910	50.01	10.00			
Day12-20 µg/mL-1	1397510	22.31	1.12	1.14	0.04	3.1
Day12-20 µg/mL-2	1413540	22.57	1.13			
Day12-20 µg/mL-3	1481100	23.65	1.18			
Day12-2 µg/mL-1	319104	5.10	0.10	0.10	0.00	4.9
Day12-2 µg/mL-2	322459	5.15	0.10			
Day12-2 µg/mL-3	294439	4.70	0.09			

Supplementary Table S37 Standard curve data for the active substance trimetoprim (TMP). A calibration curve based on the concentrations and their corresponding peak areas can be used to determine the concentration of substances in the samples.

STD concentration (ng/mL)	Peak area (Int unit)	Linearity (y=ax+b)	
100	9912990	a (slope)	99266.18618
50	4948370	b (intercept)	0
20	2038210	r	0.99995
10	1089480	r^2	0.99990
5	513115	Requirement: r ≥ 0.99	

STD - standard solutions concentration; a - slope; b - y-axis intercept; r - correlation coefficient; r2 - coefficient of determination

Supplementary Table S38 Repeatability test data for the active substance trimetoprim (TMP) in the analytical assay. It provides information on the consistency of the replicates, which is important for assessing the reliability of analytical measurements. The low CV% value shown here is favorable because it indicates that the analytical process is stable and repeatable.

Repeatability		
Sample	Area	c (TMP; STD) (ng/mL)
Repeat1	2556770	25.76
Repeat2	2582360	26.01
Repeat3	2562070	25.81
Repeat4	2556770	25.76
Repeat5	2652390	26.72
Repeat6	2530320	25.49
Requirement: CV% < 10	Mean	25.92
	SD	0.42
	CV%	1.64

SD - standard deviation; CV% - variance between replicates

Supplementary Table S39 Determination of trimetoprim (TMP) limits. These data are key to assessing the sensitivity and accuracy of the analytical method. Low LOD (limit of detection) and LOQ (limit of quantitation) values indicate good sensitivity, but high CV% suggests that background noise shows considerable variability, which may affect the accuracy of determining very low concentrations.

**LOD, LOQ**				
Sample	Area	c (TMP; STD) (ng/mL)	LOD (ng/mL)	LOQ (ng/mL)
Noise1	1266	0.01	0.04	0.12
Noise2	1277	0.01		
Noise3	1035	0.01		
Noise4	1895	0.02		
Noise5	1388	0.01		
Noise6	1035	0.01		
Noise7	771	0.01		
Mean	1238	0.01		
SD	355.39	0.00		
CV%	28.7	28.7		

SD - standard deviation; CV% - variance between replicates

Supplementary Table S40 System assay for the measurement of the active substance trimetoprim (TRI). The CV% values are quite low, suggesting that the system is performing relatively consistently, the chromatography system is working properly and is suitable for analysis.

**System suitability**			
*t*0 (min) = 0.569			
	k'	N	Asymmetry factor
STD-100	3.130	8506	1.37
STD-50	3.139	8542	1.37
STD-20	3.141	6281	1.35
Mean	3.136	7777	1.36
SD	0.006	1295	0.01
CV%	0.180	16.7	0.85
k'	> 2		
N	≥ 2000		
Asymmetry factor	≤ 2		

t0 – dead time; k' – capacity factor; N – theoretical number of plates; SD - standard deviation; CV% - variance between replicates

Supplementary Table S41 Analytical reliability of sulfamethoxazole (SMEOX) samples. The standard deviation (Stdev) measures the deviation of individual measurements from the mean. Low values of the relative standard deviation (RSD%) indicate that measurements are consistent.

Sample	Peak area (counts)	c (SMEOX basis; STD) (ng/mL)	c (SMEOX basis; Sample) (µg/mL)	c (SMEOX basis; Sample) mean (µg/mL)	Stdev	CV%
Day0-STOCK SOLUTION-1	2067730	48.76	19503.96	19573.00	237.8	1.2
Day0-STOCK SOLUTION-2	2103110	49.59	19837.68			
Day0-STOCK SOLUTION-3	2054310	48.44	19377.37			
Day0-2000 µg/mL-1	2148380	50.66	2026.47	2002.74	35.3	1.8
Day0-2000 µg/mL-2	2141090	50.49	2019.59			
Day0-2000 µg/mL-3	2080210	49.05	1962.17			
Day0-200 µg/mL-1	2147270	50.64	202.54	201.86	5.3	2.6
Day0-200 µg/mL-2	2192330	51.70	206.79			
Day0-200 µg/mL-3	2080650	49.06	196.26			
Day0-20 µg/mL-1	2208450	52.08	20.83	21.13	0.3	1.3
Day0-20 µg/mL-2	2265310	53.42	21.37			
Day0-20 µg/mL-3	2246960	52.99	21.19			
Day0-2 µg/mL-1	1066500	25.15	2.51	2.56	0.2	6.4
Day0-2 µg/mL-2	1030690	24.31	2.43			
Day0-2 µg/mL-3	1165750	27.49	2.75			
Day1-STOCK SOLUTION-1	2086760	49.49	19794.94	19899.89	119.9	0.6
Day1-STOCK SOLUTION-2	2111600	50.08	20030.58			
Day1-STOCK SOLUTION-3	2095110	49.69	19874.15			
Day1-2000 µg/mL-1	2142940	50.82	2032.79	2034.85	18.7	0.9
Day1-2000 µg/mL-2	2165840	51.36	2054.51			
Day1-2000 µg/mL-3	2126570	50.43	2017.26			
Day1-200 µg/mL-1	2094590	49.67	198.69	205.90	6.7	3.3
Day1-200 µg/mL-2	2181990	51.75	206.98			
Day1-200 µg/mL-3	2235100	53.01	212.02			
Day1-20 µg/mL-1	2145950	50.89	20.36	20.98	0.6	2.6
Day1-20 µg/mL-2	2255870	53.50	21.40			
Day1-20 µg/mL-3	2234490	52.99	21.20			
Day1-2 µg/mL-1	1035360	24.55	2.46	2.44	0.0	1.3
Day1-2 µg/mL-2	1011470	23.99	2.40			
Day1-2 µg/mL-3	1035120	24.55	2.45			
Day2-STOCK SOLUTION-1	2071010	49.72	19888.57	19608.76	243.1	1.2
Day2-STOCK SOLUTION-2	2029350	48.72	19488.50			

Day2-STOCK SOLUTION-3	2025260	48.62	19449.22			
Day2-2000 μg/mL-1	2127330	51.07	2042.94	2094.59	51.5	2.5
Day2-2000 μg/mL-2	2234490	53.65	2145.85			
Day2-2000 μg/mL-3	2181500	52.37	2094.96			
Day2-200 μg/mL-1	2173500	52.18	208.73	203.09	5.2	2.5
Day2-200 μg/mL-2	2068320	49.66	198.63			
Day2-200 μg/mL-3	2102690	50.48	201.93			
Day2-20 μg/mL-1	2201630	52.86	21.14	21.20	0.3	1.3
Day2-20 μg/mL-2	2238410	53.74	21.50			
Day2-20 μg/mL-3	2181500	52.37	20.95			
Day2-2 μg/mL-1	1052910	25.28	2.53	2.47	0.0	1.9
Day2-2 μg/mL-2	1023760	24.58	2.46			
Day2-2 μg/mL-3	1015590	24.38	2.44			
Day5-STOCK SOLUTION-1	1658580	49.15	19660.80	20370.45	985.1	4.8
Day5-STOCK SOLUTION-2	1683430	49.89	19955.37			
Day5-STOCK SOLUTION-3	1813330	53.74	21495.20			
Day5-2000 μg/mL-1	1621420	48.05	1922.03	1975.87	54.0	2.7
Day5-2000 μg/mL-2	1712520	50.75	2030.02			
Day5-2000 μg/mL-3	1666570	49.39	1975.55			
Day5-200 μg/mL-1	1681620	49.83	199.34	202.41	2.7	1.3
Day5-200 μg/mL-2	1725260	51.13	204.51			
Day5-200 μg/mL-3	1715640	50.84	203.37			
Day5-20 μg/mL-1	1745890	51.74	20.70	20.66	0.0	0.2
Day5-20 μg/mL-2	1743360	51.66	20.67			
Day5-20 μg/mL-3	1739500	51.55	20.62			
Day5-2 μg/mL-1	795466	23.57	2.36	2.37	0.0	1.9
Day5-2 μg/mL-2	789380	23.39	2.34			
Day5-2 μg/mL-3	817681	24.23	2.42			
Day7-STOCK SOLUTION-1	1675680	48.20	19280.28	19524.25	256.99	1.3
Day7-STOCK SOLUTION-2	1720200	49.48	19792.53			
Day7-STOCK SOLUTION-3	1694770	48.75	19499.93			
Day7-2000 μg/mL-1	1675850	48.21	1928.22	1963.49	32.66	1.7
Day7-2000 μg/mL-2	1731890	49.82	1992.70			
Day7-2000 μg/mL-3	1711770	49.24	1969.55			
Day7-200 μg/mL-1	1713580	49.29	197.16	200.23	3.42	1.7
Day7-200 μg/mL-2	1772360	50.98	203.93			
Day7-200 μg/mL-3	1734880	49.90	199.61			
Day7-20 μg/mL-1	1767510	50.84	20.34	19.88	0.40	2.0
Day7-20 μg/mL-2	1713660	49.29	19.72			
Day7-20 μg/mL-3	1702820	48.98	19.59			

Day7-2 μg/mL-1	784167	22.56	2.26	2.25	0.05	2.3
Day7-2 μg/mL-2	764159	21.98	2.20			
Day7-2 μg/mL-3	800478	23.03	2.30			
Day9-STOCK SOLUTION-1	1646780	49.62	19849.45	19825.74	70.21	0.4
Day9-STOCK SOLUTION-2	1638260	49.37	19746.75			
Day9-STOCK SOLUTION-3	1649400	49.70	19881.03			
Day9-2000 μg/mL-1	1611410	48.56	1942.31	1941.05	4.25	0.2
Day9-2000 μg/mL-2	1613250	48.61	1944.53			
Day9-2000 μg/mL-3	1606440	48.41	1936.32			
Day9-200 μg/mL-1	1643890	49.54	198.15	196.68	2.18	1.1
Day9-200 μg/mL-2	1640300	49.43	197.71			
Day9-200 μg/mL-3	1610890	48.54	194.17			
Day9-20 μg/mL-1	1628150	49.06	19.62	19.54	0.13	0.7
Day9-20 μg/mL-2	1626850	49.02	19.61			
Day9-20 μg/mL-3	1608850	48.48	19.39			
Day9-2 μg/mL-1	716647	21.60	2.16	2.13	0.03	1.3
Day9-2 μg/mL-2	706302	21.28	2.13			
Day9-2 μg/mL-3	698068	21.04	2.10			
Day12-STOCK SOLUTION-1	1583780	49.33	19730.34	19407.56	294.4	1.5
Day12-STOCK SOLUTION-2	1537490	47.88	19153.67			
Day12-STOCK SOLUTION-3	1552340	48.35	19338.67			
Day12-2000 μg/mL-1	1488040	46.34	1853.76	1895.02	35.9	1.9
Day12-2000 μg/mL-2	1540460	47.98	1919.07			
Day12-2000 μg/mL-3	1534960	47.81	1912.22			
Day12-200 μg/mL-1	1532490	47.73	190.91	190.82	1.6	0.9
Day12-200 μg/mL-2	1518340	47.29	189.15			
Day12-200 μg/mL-3	1544350	48.10	192.39			
Day12-20 μg/mL-1	1536150	47.84	19.14	18.98	0.1	0.7
Day12-20 μg/mL-2	1515140	47.19	18.88			
Day12-20 μg/mL-3	1518720	47.30	18.92			
Day12-2 μg/mL-1	683550	21.29	2.13	2.09	0.0	1.5
Day12-2 μg/mL-2	666449	20.76	2.08			
Day12-2 μg/mL-3	665413	20.72	2.07			

Supplementary Table S42 Standard curve data for the active substance sulfamethoxazole (SMEOX). A calibration curve based on the concentrations and their corresponding peak areas can be used to determine the concentration of substances in the samples.

STD concentration (ng/mL)	Peak area (Int unit)	Linearity (y=ax+b)	
100	4232220	a (slope)	42406.36699
50	2135150	b (intercept)	0
20	849380	r	0.99999
10	429145	r^2	0.99999
5	216876	Requirement: r ≥ 0.99	

STD - standard solutions concentration; a - slope; b - y-axis intercept; r - correlation coefficient; r2 - coefficient of determination

Supplementary Table S43 Repeatability test data for the active substance sulfamethoxazole (SMEOX) in the analytical assay. It provides information on the consistency of the replicates, which is important for assessing the reliability of analytical measurements. The low CV% value shown here is favorable because it indicates that the analytical process is stable and repeatable.

Repeatability		
Sample	Area	c (SMEOX; STD) (ng/mL)
Repeat1	1152820	27.19
Repeat2	1035120	24.41
Repeat3	1014300	23.92
Repeat4	1015590	23.95
Repeat5	1017670	24.00
Repeat6	1202870	28.37
Requirement: CV% < 10	Mean	25.30
	SD	1.96
	CV%	7.74

SD - standard deviation; CV% - variance between replicates

Supplementary Table S44 Determination of sulfamethoxazole (SMEOX) limits. These data are key to assessing the sensitivity and accuracy of the analytical method. Low LOD (limit of detection) and LOQ (limit of quantitation) values indicate good sensitivity, but high CV% suggests that background noise shows considerable variability, which may affect the accuracy of determining very low concentrations.

**LOD, LOQ**				
Sample	Area	c (SMEOX; STD) (ng/mL)	LOD (ng/mL)	LOQ (ng/mL)
Noise1	137	0.00	0.01	0.03
Noise2	72	0.00		
Noise3	200	0.00		
Noise4	56	0.00		
Noise5	129	0.00		
Noise6	235	0.01		
Noise7	158	0.00		
Mean	141	0.00		
SD	64.30	0.00		
CV%	45.6	45.6		

SD - standard deviation; CV% - variance between replicates

Supplementary Table S45 System assay for the measurement of the active substance sulfamethoxazole (SMEOX). The CV% values are quite low, suggesting that the system is performing relatively consistently, the chromatography system is working properly and is suitable for analysis.

System suitability			
*t*0 (min) = 1.241			
	k'	N	Asymmetry factor
STD-100	3.925	8091	1.14
STD-50	3.925	8091	1.01
STD-20	3.946	8160	1.08
Mean	3.932	8114	1.08
SD	0.012	40	0.07
CV%	0.308	0.5	6.04
k'		> 2	
N		≥ 2000	
Asymmetry factor		≤ 2	

t0 – dead time; k' – capacity factor; N – theoretical number of plates; SD - standard deviation; CV% - variance between replicates

Supplementary Table S46 Dilutions used in the preparation of samples

Active substance	Stock solution	10× dilution	100× dilution	1000× dilution	10000× dilution
Amoxicillin	25000×	5000×	500×	100×	10×
Cefotaxime	100000×	10000×	1000×	100×	50×
Neomyicin	100000×	10000×	1000×	100×	40×
Oxytetracycline	100000×	10000×	1000×	100×	25×
Florfenicol	100000×	20000×	2000×	200×	40×
Enrofloxacin	2000×	200×	50×	10×	3×
Colistin	25000×	1000×	250×	50×	10×
Sulfamethoxazole	400000×	40000×	4000×	400×	100×
Trimethoprim	20000×	2000×	200×	50×	20×

Supplementary Table S47 Concentrations of calibration solutions and stock solutions

Active substance	Stock sol.oldat	Solution 1	Solution 2	Solution 3	Solution 4	Solution 5
		ng/mL				
Amoxicillin	100 μg/mL	500	200	100	50	20
Cefotaxime	1 mg/mL	100	50	20	10	5
Neomyicin	1 mg/mL	1000	500	200	100	50
Oxytetracycline	50 μg/mL	100	50	20	10	5
Florfenicol	1 mg/mL	500	200	100	50	20
Enrofloxacin	50 μg/mL	100	50	20	10	5
Colistin	2,5 mg/mL	500	200	100	50	20
Sulfamethoxazole	50 μg/mL	100	50	20	10	5
Trimethoprim	50 μg/mL	100	50	20	10	5

Supplementary Table S48 Ion transitions measured by the mass spectrometer. In bold is the ionic transition that was found to be the most suitable for the measurement in the tests

Active substance	[1]Q_1 mass (Da)	[1]Q_3 mass (Da)	Identity	[2]DP (V)	[3]CE (V)	[4]CXP (V)
Amoxicillin	**383.000**	**114.000**	**Amoxicillin_NH_4 1**	**16**	**31**	**8**
	383.000	349.000	Amoxicillin_NH_4 2	16	23	16
	366.000	114.000	Amoxicillin_prot	106	15	12
Cefotaxime	456.200	396.000	Cefotaxime 1	91	21	12
	456.200	241.000	Cefotaxime 2	91	23	10
	**456.200**	**125.000**	**Cefotaxime 3**	**91**	**77**	**10**
Neomycin	615.500	161.100	Neomycin-1	151	43	12
	615.500	163.100	Neomycin-2	146	37	12
	615.500	293.300	Neomycin-3	146	33	24
	**308.400**	**161.100**	**Neomycin-4**	**56**	**25**	**12**
Oxytetracycline	**461.000**	**426.000**	**Oxytetracycline 1**	**91**	**31**	**14**
	461.000	443.000	Oxytetracycline 2	96	19	14
Florfenicol	**375.200**	**241.000**	**Florfenicol_NH_4-1**	**66**	**33**	**10**
	375.200	206.100	Florfenicol_NH_4-2	31	41	8
	377.200	243.000	Florfenicol_NH_4-3	31	37	6
	377.200	208.100	Florfenicol_NH_4-4	21	35	6
Enrofloxacin	360.400	316.200	Enrofloxacin 1	96	29	12
	**360.400**	**342.200**	**Enrofloxacin 2**	**101**	**31**	**6**
Colistin	585.600	101.100	Polymyxin_E1-1	121	71	8
	**390.900**	**101.100**	**Polymyxin_E1-2**	**71**	**25**	**8**
	578.500	101.100	Polymyxin_E2-1	121	59	10
	**386.200**	**101.100**	**Polymyxin_E2-2**	**71**	**23**	**10**
Sulfamethoxazole	**254.000**	**156.000**	**Sulfamethoxazole 1**	**71**	**25**	**6**
	254.000	108.000	Sulfamethoxazole 2	81	31	8
Trimethoprim	**291.000**	**230.000**	**Trimetoprim 1**	**76**	**35**	**8**
	291.000	123.000	Trimetoprim 2	76	35	10

[1]quadrupole; [2] declustering potential; [3]collision energy; [4]collision cell exit potential

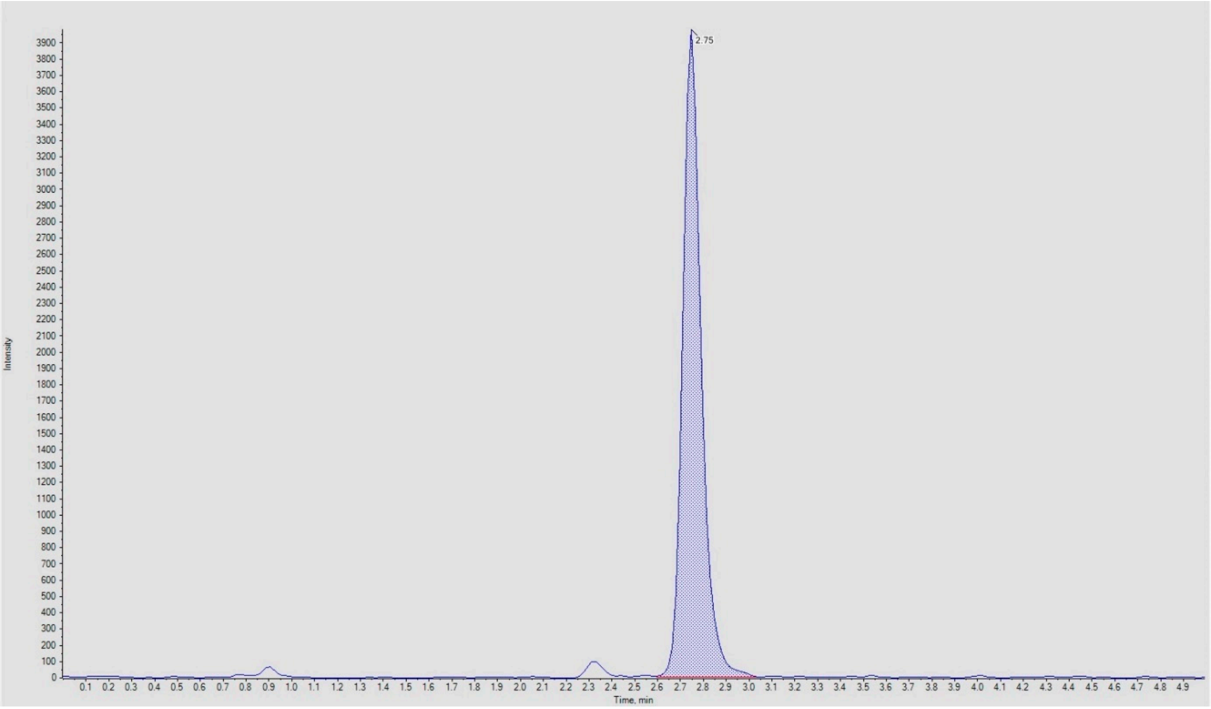

Supplementary Figure S1. Amoxicillin (AMX) Chromatogram: The dominant peak on the graph occurs at a retention time of approximately 2.75 minutes, indicating the arrival time of this component at the detector. The presence of smaller peaks likely represents noise from the measurement rather than significant substances. Furthermore, the chromatogram demonstrates good separation of the major peak; it is well-resolved and symmetric with no significant adjacent peaks, ensuring that this component is distinctly separated from others, allowing for accurate measurement.

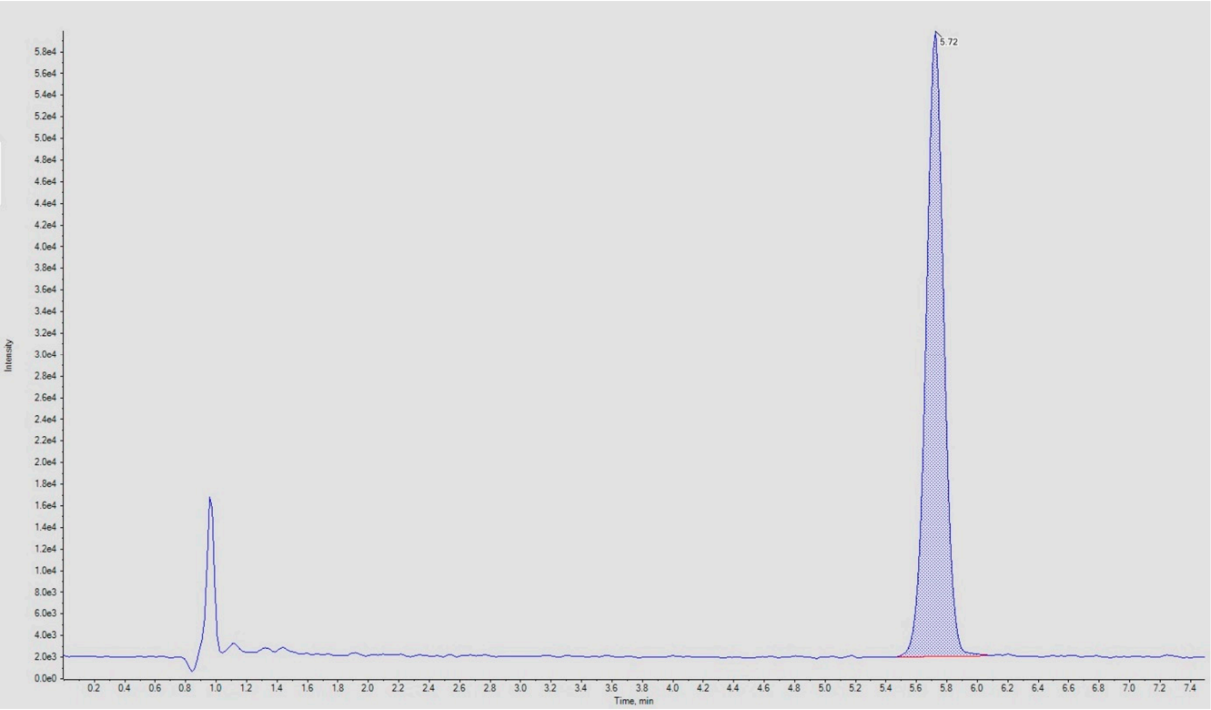

Supplementary Figure S2 Cefotaxime (CTXM) Chromatogram: The dominant peak on the graph occurs at a retention time of approximately 5.72 minutes, indicating the arrival time of this component at the detector. The presence of the smaller peak at around the minute mark represents the dead volume; the other smaller peaks

represent noise. Furthermore, the chromatogram demonstrates good separation of the major peak; it is well-resolved and symmetric with no significant adjacent peaks, ensuring that this component is distinctly separated from others, allowing for accurate measurement.

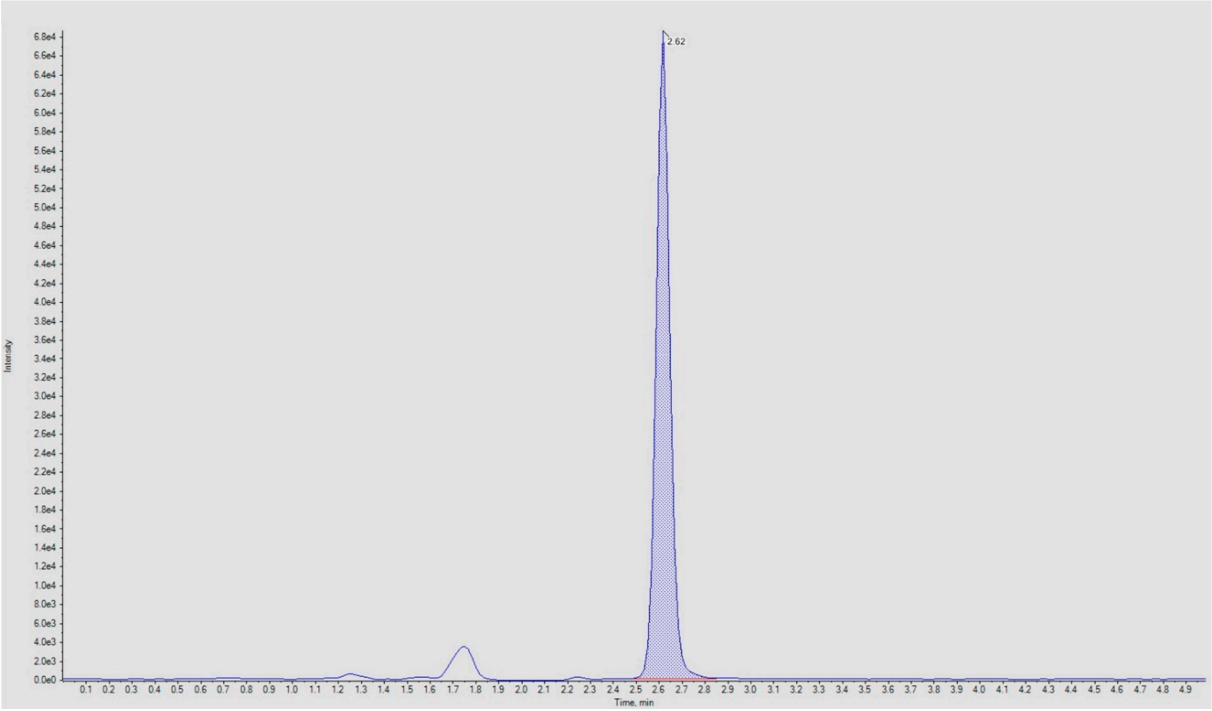

Supplementary Figure S3 Oxytetracycline (OTC) Chromatogram: The dominant peak on the graph occurs at a retention time of approximately 2.62 minutes, indicating the arrival time of this component at the detector. The presence of smaller peaks likely represents noise from the measurement rather than significant substances. Furthermore, the chromatogram demonstrates good separation of the major peak; it is well-resolved and symmetric with no significant adjacent peaks, ensuring that this component is distinctly separated from others, allowing for accurate measurement.

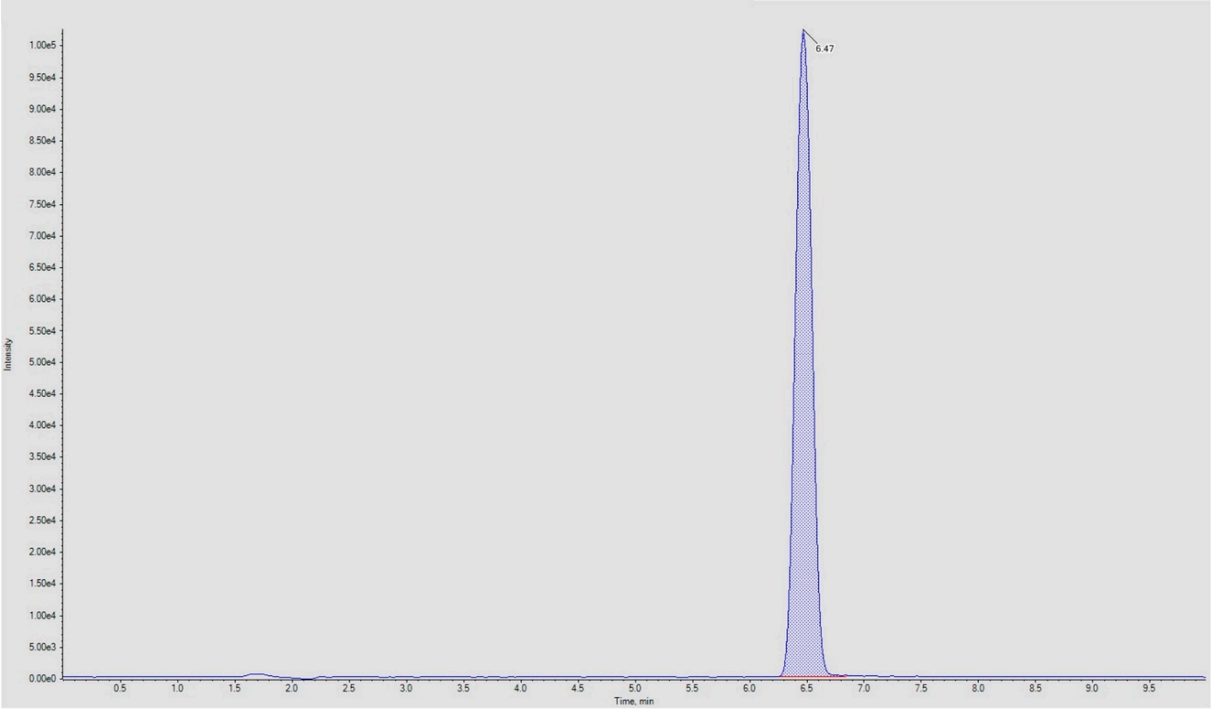

Supplementary Figure S4 Florfenicol (FLO) Chromatogram: The dominant peak on the graph occurs at a retention time of approximately 6.47 minutes, indicating the arrival time of this component at the detector. The presence of smaller peaks likely represents noise from the measurement rather than significant substances. Furthermore, the chromatogram demonstrates good separation of the major peak; it is well-resolved and symmetric with no significant adjacent peaks, ensuring that this component is distinctly separated from others, allowing for accurate measurement.

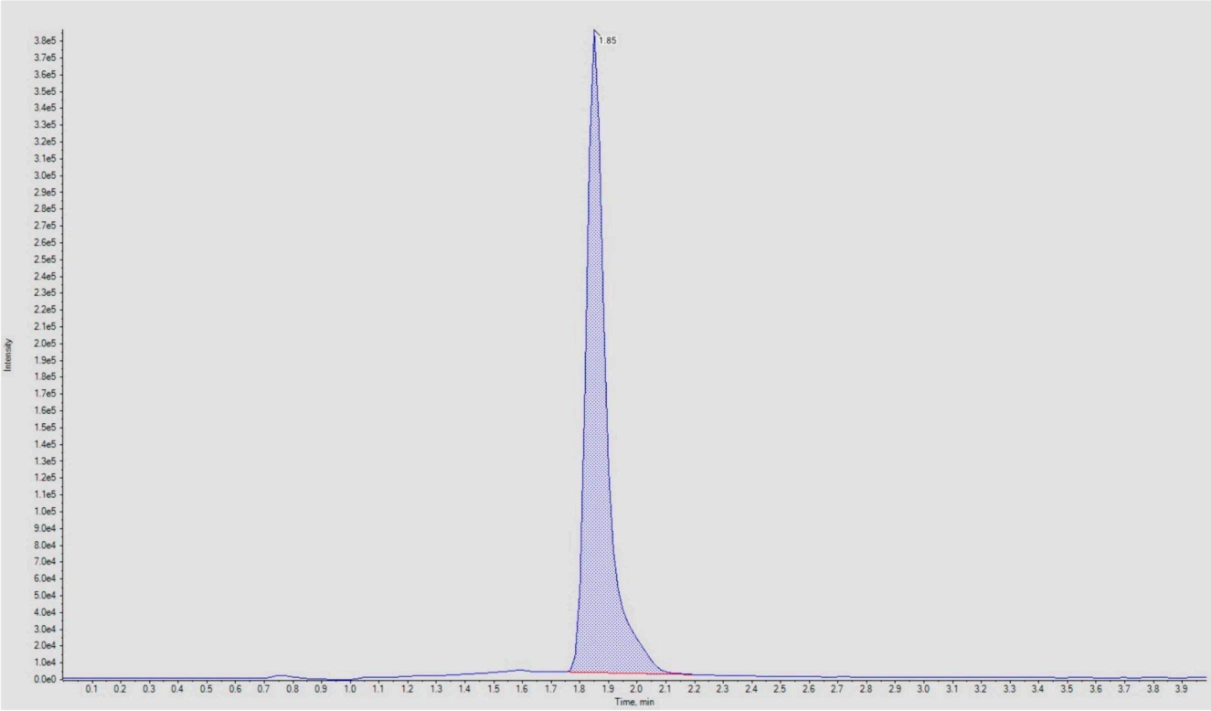

Supplementary Figure S5 Enrofloxacin (ENFX) chromatogram: The dominant peak on the graph occurs at a retention time of approximately 1.85 minutes, indicating the arrival time of this component at the detector. The

presence of smaller peaks likely represents noise from the measurement rather than significant substances. Furthermore, the chromatogram demonstrates good separation of the major peak; it is well-resolved and symmetric with no significant adjacent peaks and only with slight tailing, ensuring that this component is distinctly separated from others, allowing for accurate measurement.

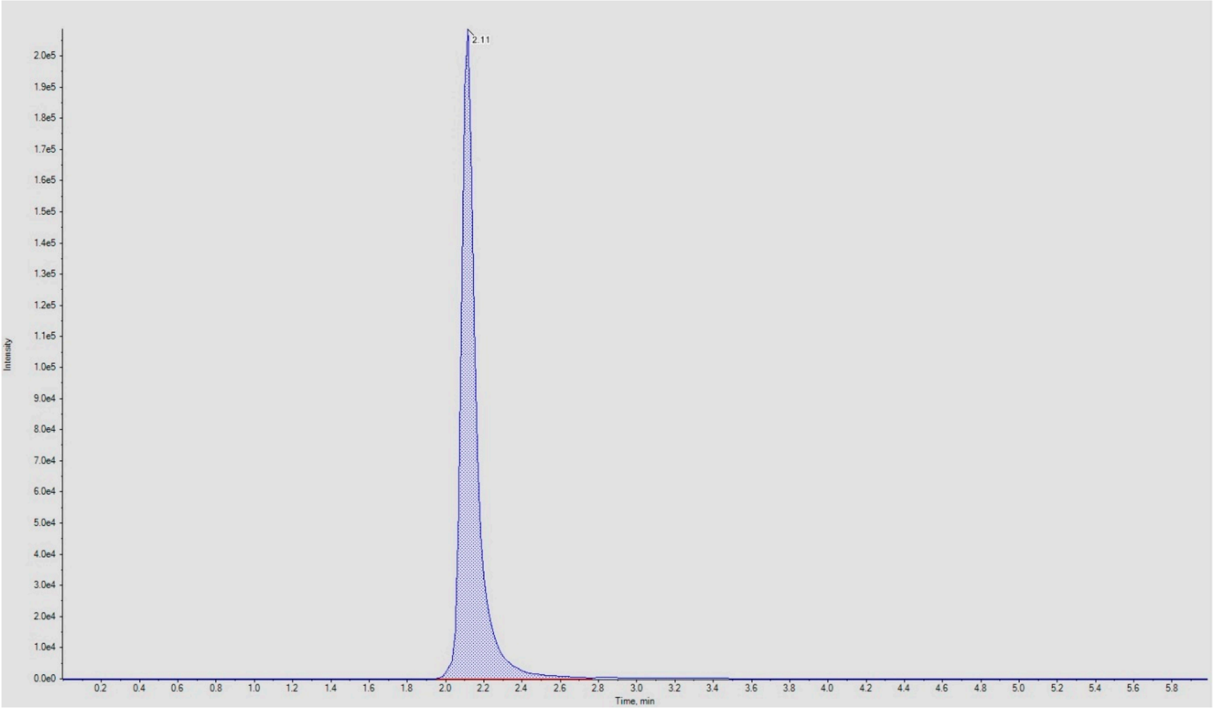

Supplementary Figure S6 Colistin (Polymixin E-2) The dominant peak on the graph occurs at a retention time of approximately 2.11 minutes, indicating the arrival time of this component at the detector. The presence of smaller peaks likely represents noise from the measurement rather than significant substances. Furthermore, the chromatogram demonstrates good separation of the major peak; it is well-resolved and symmetric with no significant adjacent peaks and only with slight tailing, ensuring that this component is distinctly separated from others, allowing for accurate measurement.

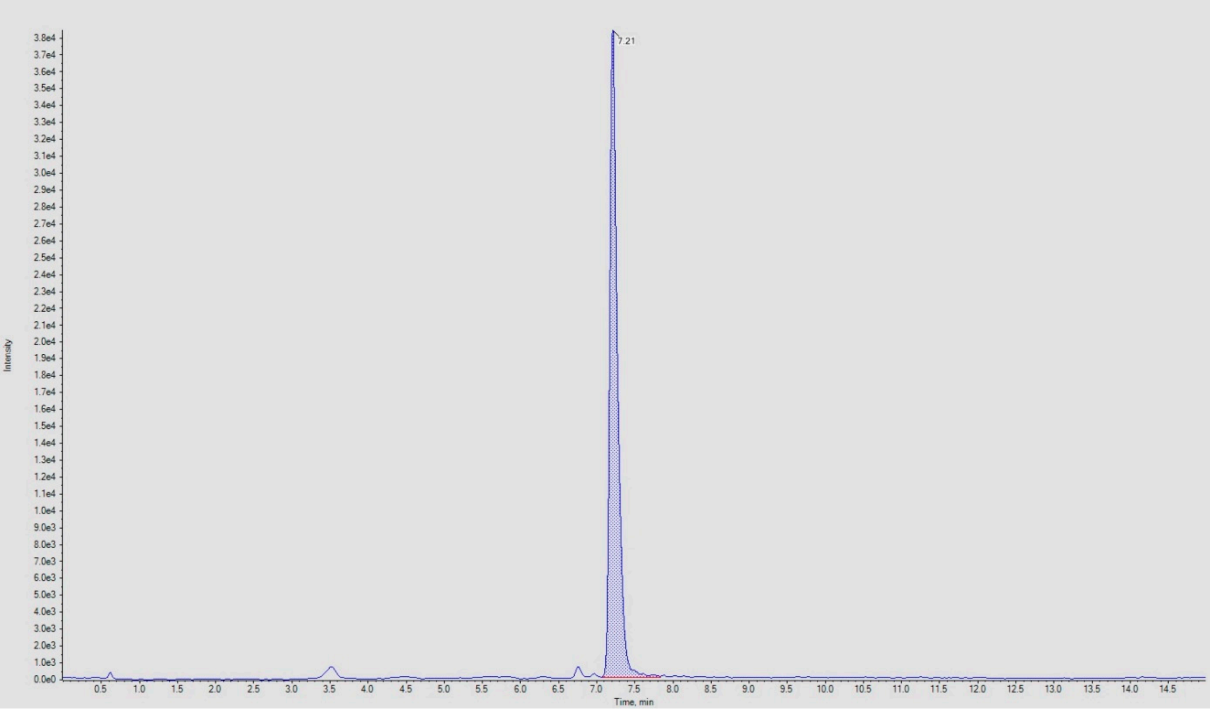

Supplementary Figure S7 Neomycin (NEO) Chromatogram: The dominant peak on the graph occurs at a retention time of approximately 7.11 minutes, indicating the arrival time of this component at the detector. The presence of smaller peaks likely represents noise from the measurement rather than significant substances. Furthermore, the chromatogram demonstrates good separation of the major peak; it is well-resolved and symmetric with no significant adjacent peaks, ensuring that this component is distinctly separated from others, allowing for accurate measurement.

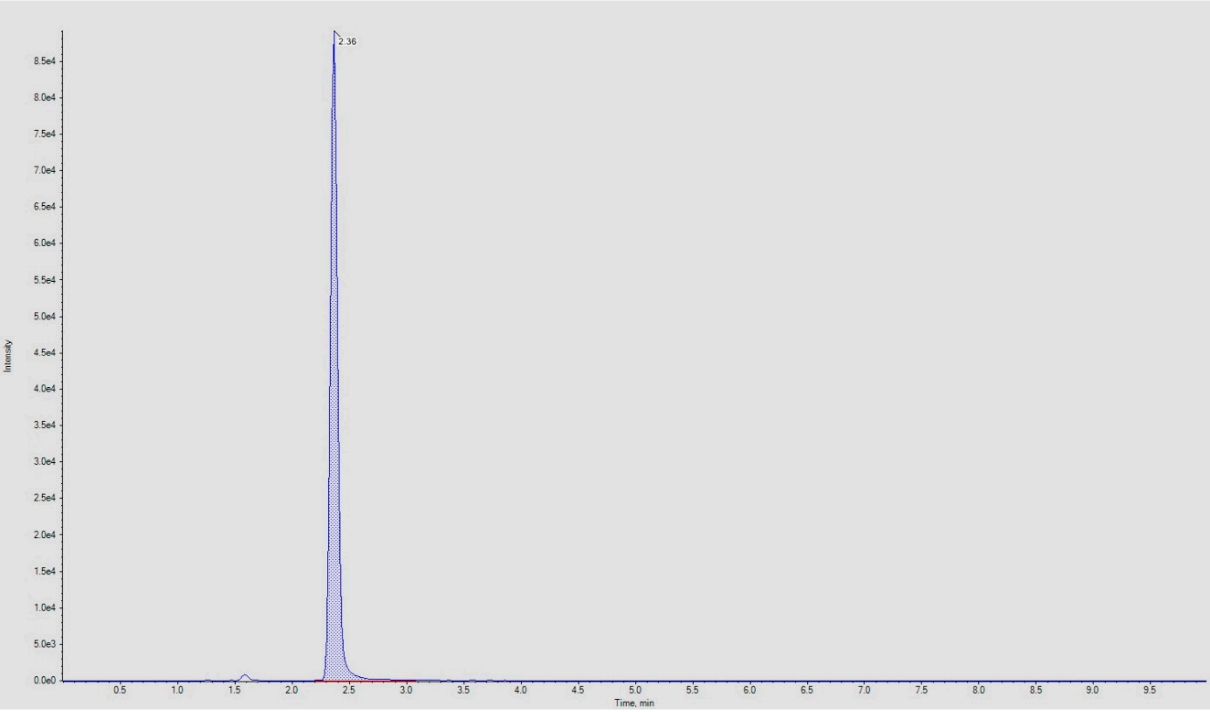

Supplementary Figure S8 Trimethoprim (TMP) Chromatogram: The dominant peak on the graph occurs at a retention time of approximately 2.36 minutes, indicating the arrival time of this component at the detector. The

presence of smaller peaks likely represents noise from the measurement rather than significant substances. Furthermore, the chromatogram demonstrates good separation of the major peak; it is well-resolved and symmetric with no significant adjacent peaks, ensuring that this component is distinctly separated from others, allowing for accurate measurement.

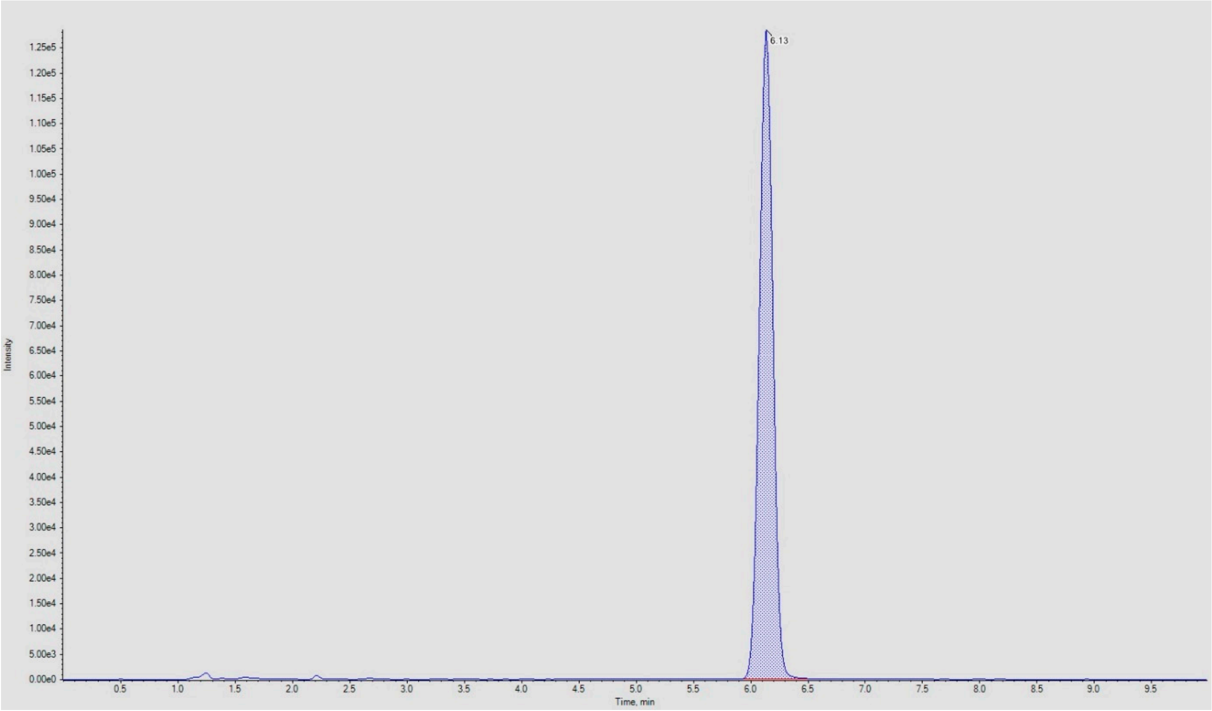

Supplementary Figure S9 Sulfamethoxazole (SMEOX) Chromatogram: The dominant peak on the graph occurs at a retention time of approximately 6.13 minutes, indicating the arrival time of this component at the detector. The presence of smaller peaks likely represents noise from the measurement rather than significant substances. Furthermore, the chromatogram demonstrates good separation of the major peak; it is well-resolved and symmetric with no significant adjacent peaks, ensuring that this component is distinctly separated from others, allowing for accurate measurement.

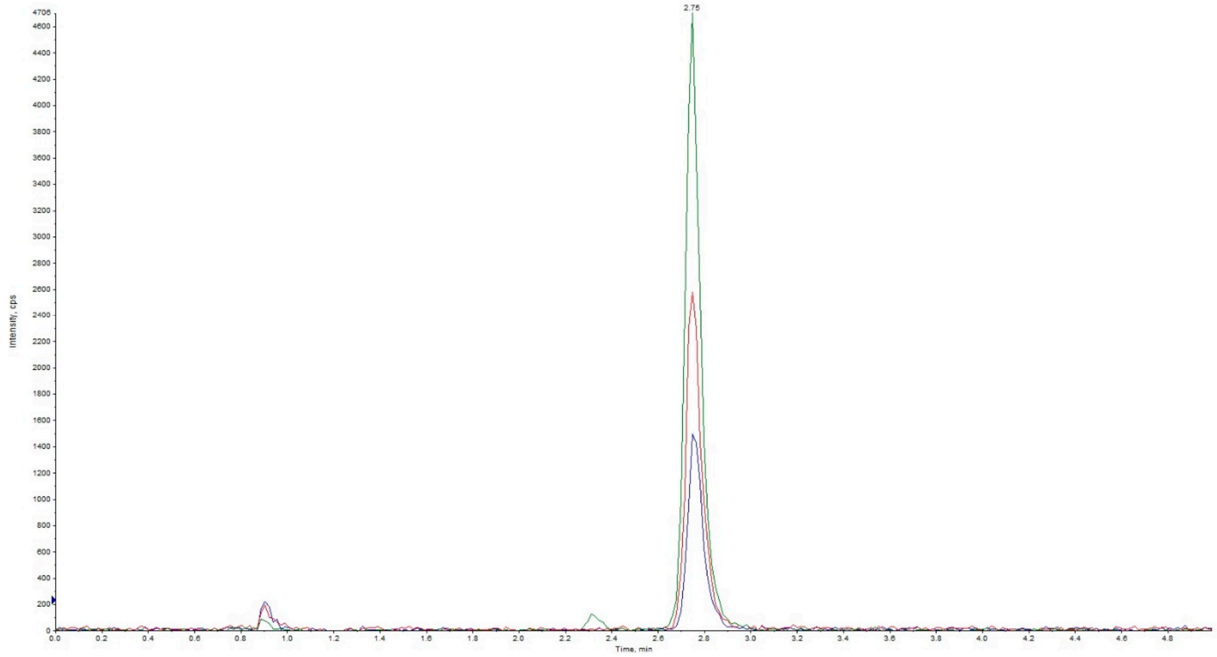

Supplementary Figure S10 Amoxicillin (AMX) Overlay chromatogram: The dominant peak on the graph occurs at a retention time of approximately 2.75 minutes, indicating the arrival time of this component at the detector.

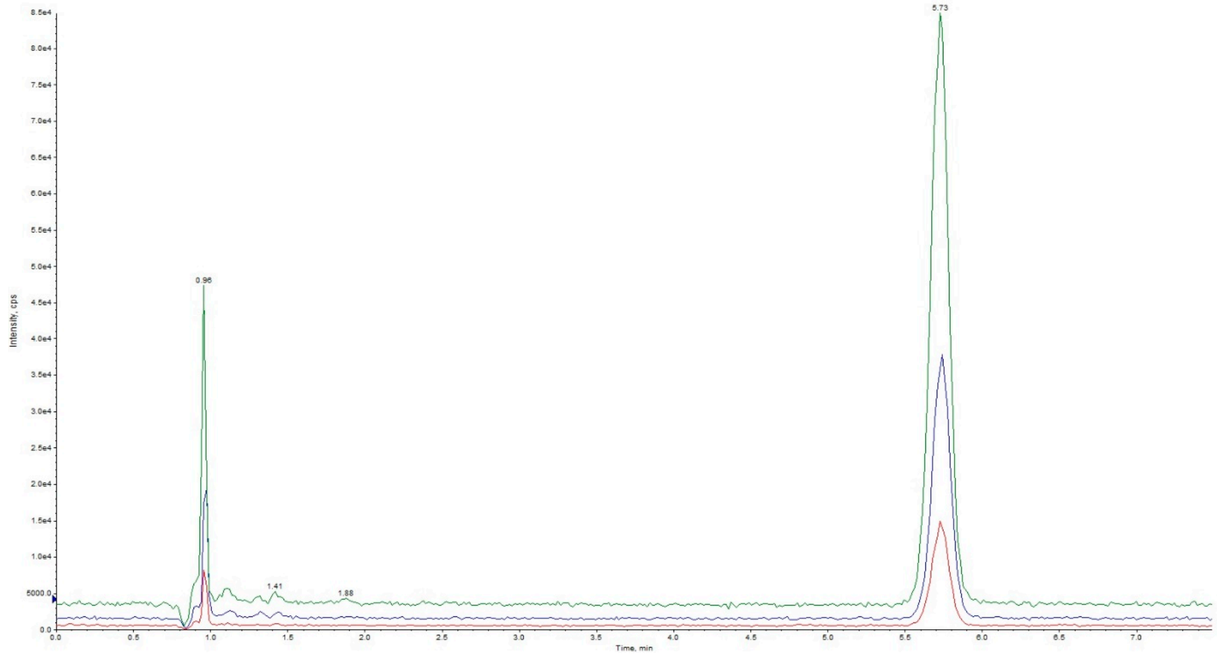

Supplementary Figure S11 Cefotaxime (CTXM) Overlay chromatogram*: The dominant peak on the graph occurs at a retention time of approximately 5.73 minutes, indicating the arrival time of this component at the detector.*

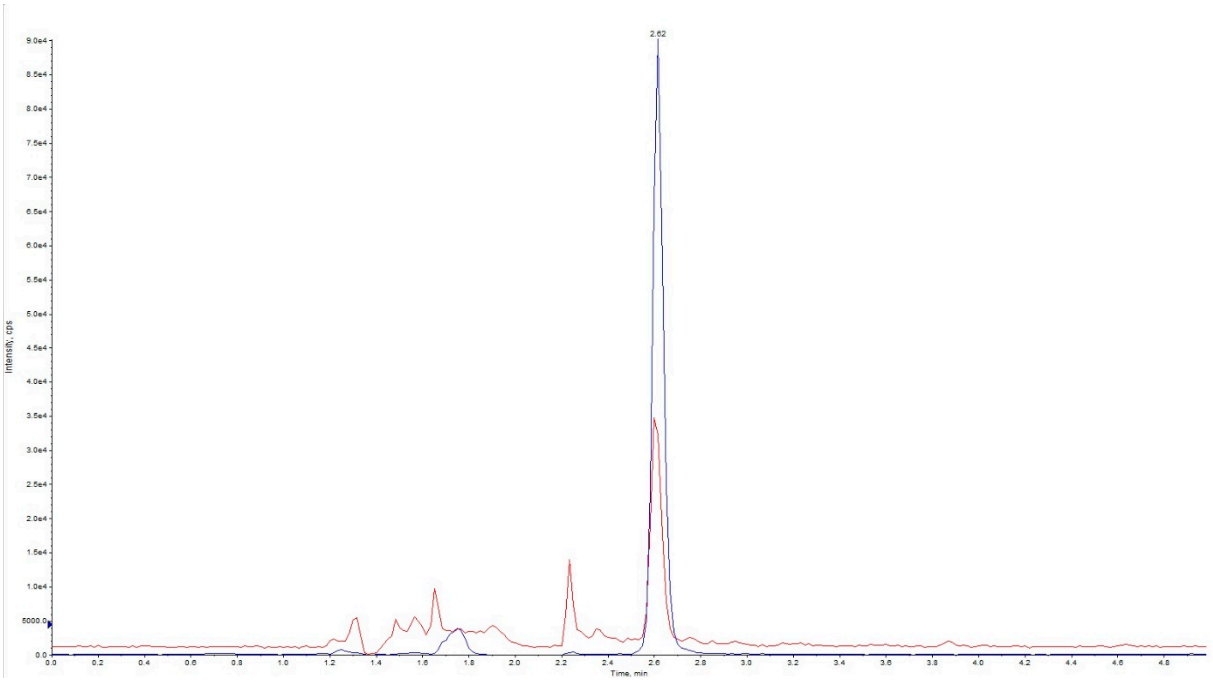

Supplementary Figure S12 Oxytetracycline (OTC) Overlay chromatogram*: The dominant peak on the graph occurs at a retention time of approximately 2.62 minutes, indicating the arrival time of this component at the detector.*

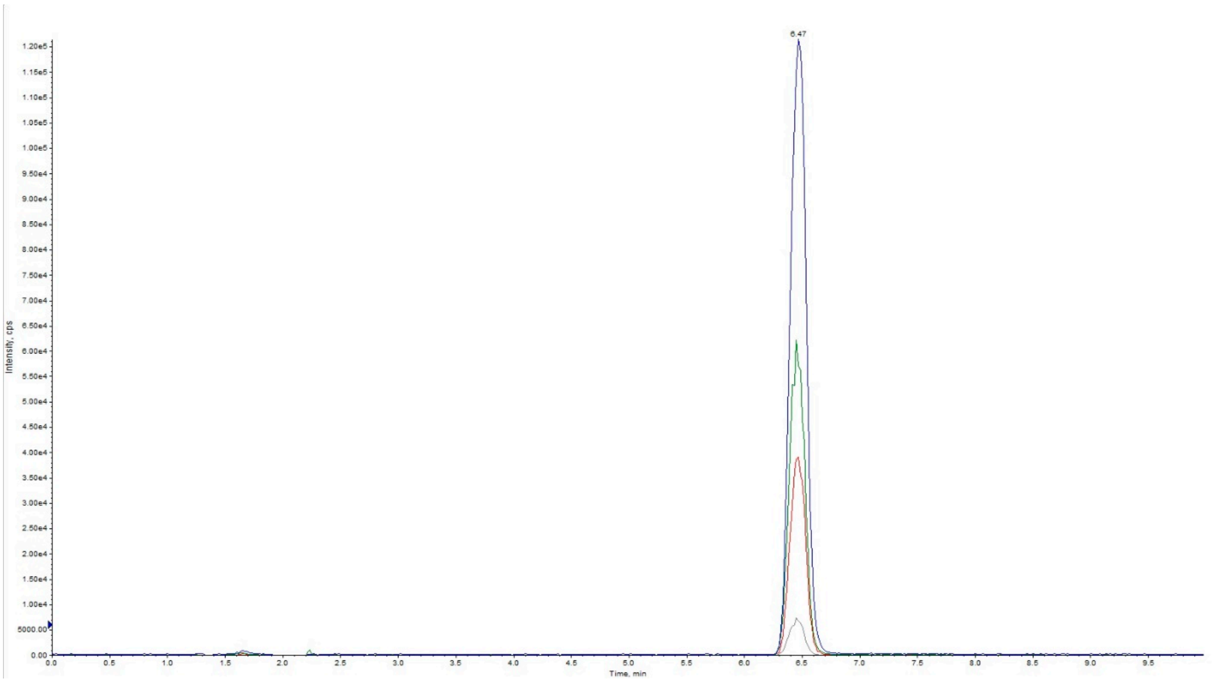

Supplementary Figure S13 Florfenicol (FLO) Overlay chromatogram*: The dominant peak on the graph occurs at a retention time of approximately 6.47 minutes, indicating the arrival time of this component at the detector.*

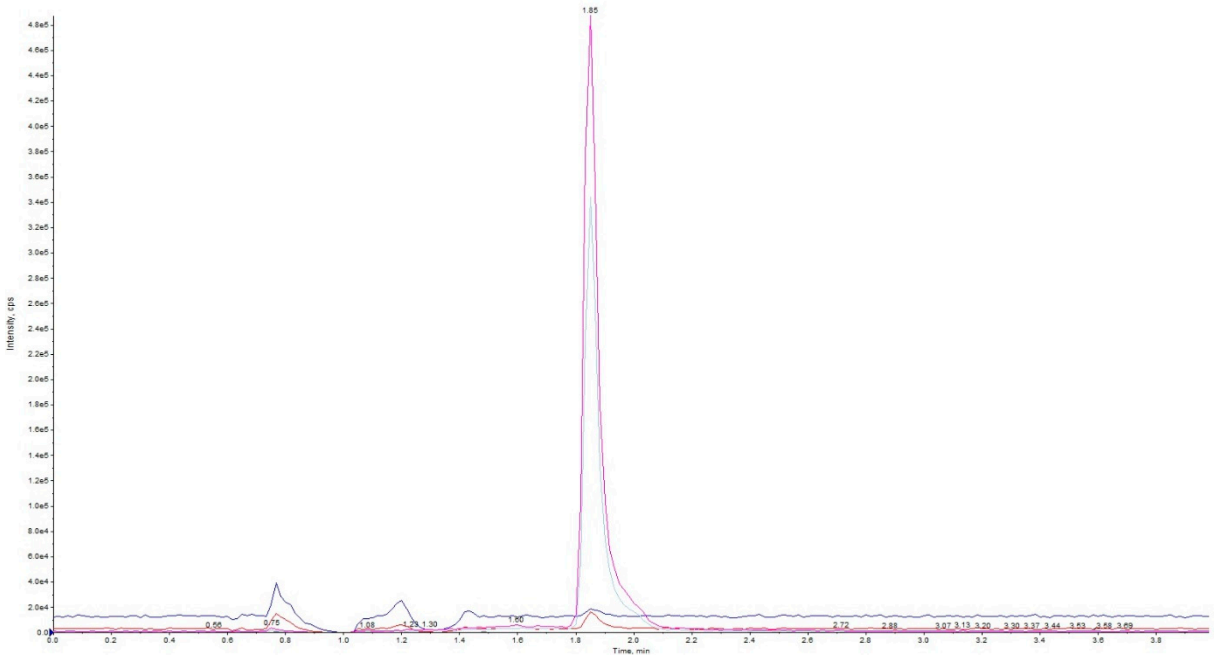

Supplementary Figure S14 Enrofloxacin (ENFX) Overlay chromatogram*: The dominant peak on the graph occurs at a retention time of approximately 1.85 minutes, indicating the arrival time of this component at the detector. Significantly more transition chromatograms are visible because the method was originally developed for the simultaneous determination of levofloxacin, enrofloxacin, ciprofloxacin, and moxifloxacin.*

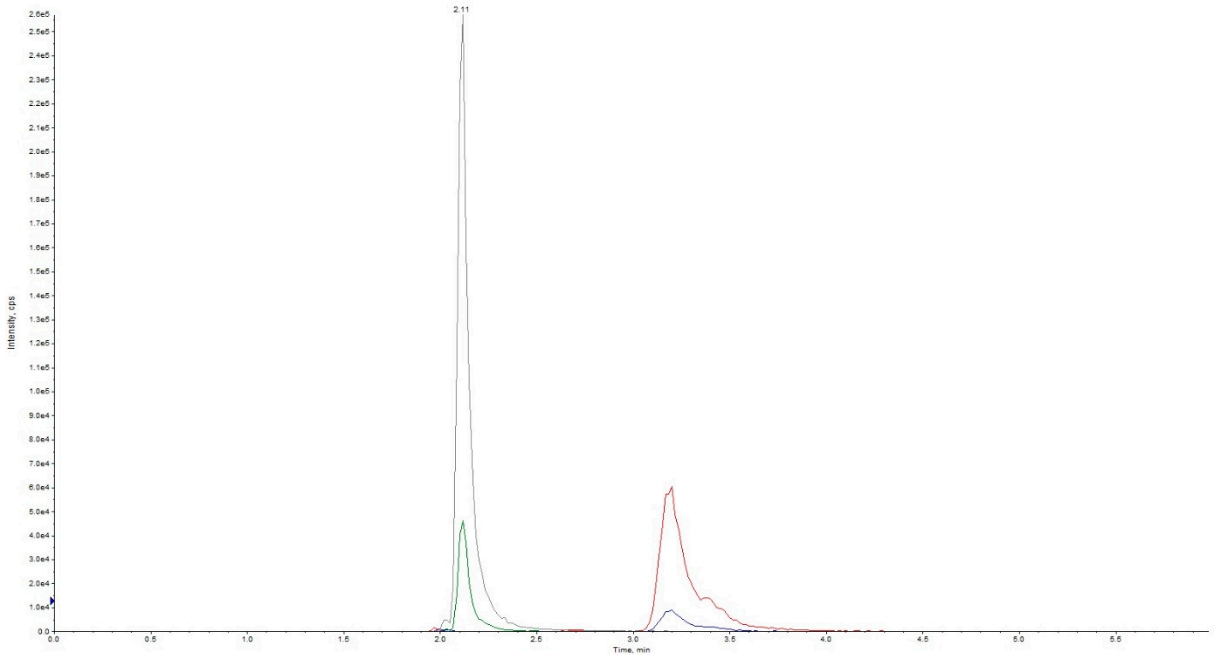

Supplementary Figure S15 Colistin (COL) Overlay chromatogram: *The dominant peak on the graph occurs at a retention time of approximately 2.11 minutes, indicating the arrival time of this component at the detector. The second peak pair corresponds to Polymyxin E-1, while we examined Polymyxin E-2 (more sensitive, constant ratio due to the standard).*

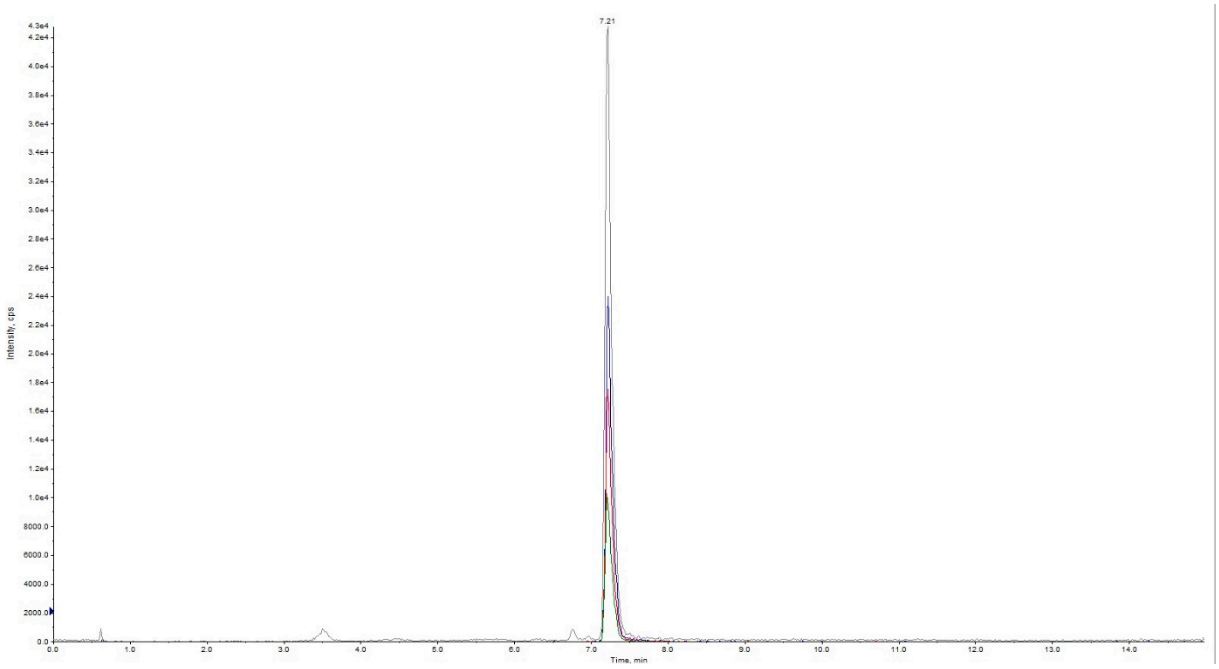

Supplementary Figure S16 Neomycin (NEO) Overlay chromatogram*: The dominant peak on the graph occurs at a retention time of approximately 7.21 minutes, indicating the arrival time of this component at the detector.*

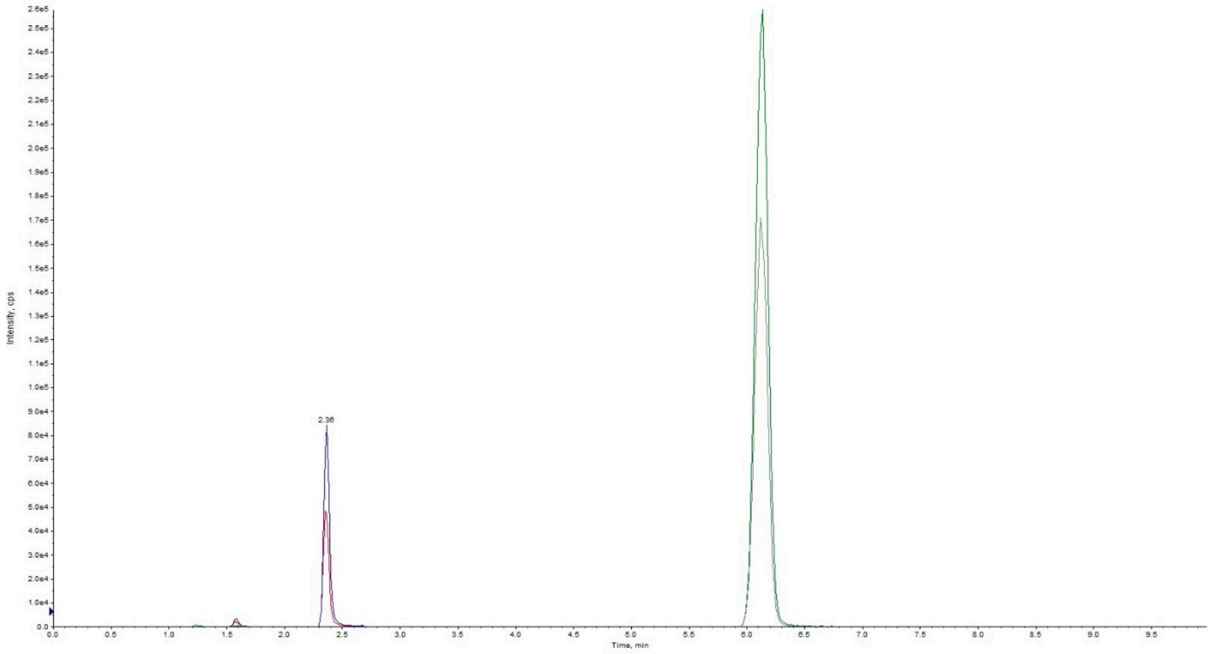

Supplementary Figure S17 Trimethoprim (TMP) Overlay chromatogram*: The dominant peak on the graph occurs at a retention time of approximately 2.36 minutes, indicating the arrival time of this component at the detector.*

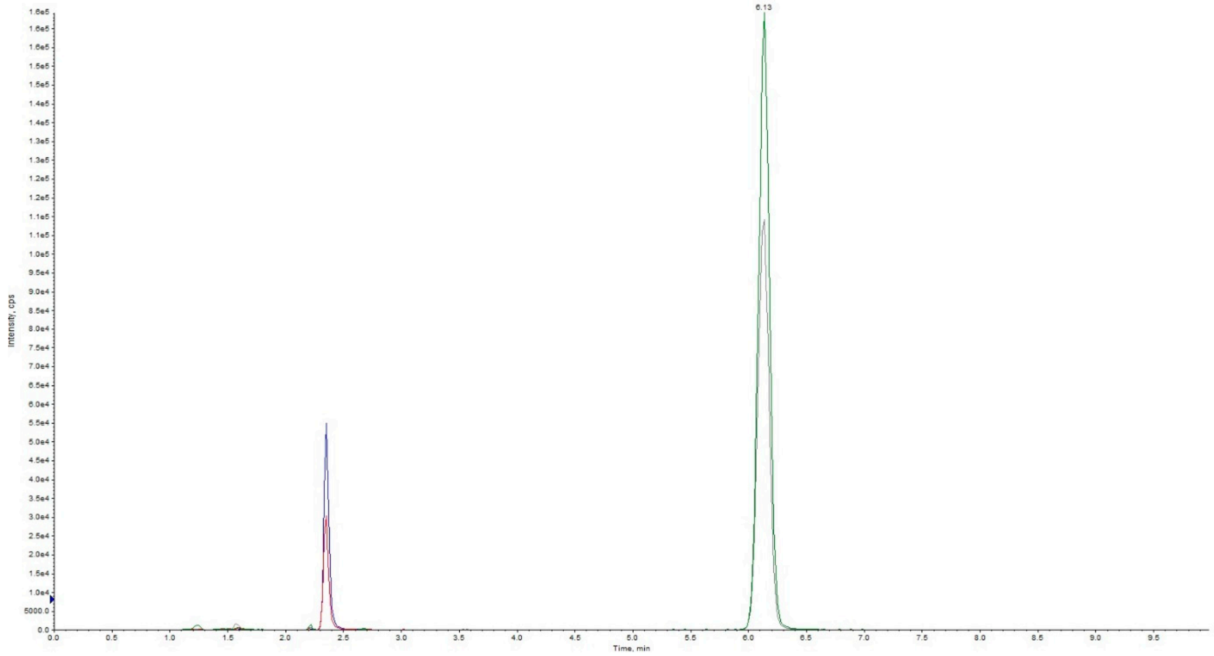

Supplementary Figure S18 Sulfamethoxazole (SMEOX) Overlay chromatogram*: The dominant peak on the graph occurs at a retention time of approximately 6.13 minutes, indicating the arrival time of this component at the detector.*

Supplementary Table S49 Stability of aqueous stock solutions and dilution series per active substance on each measurement day

			Mean % of initial concentration ± RSD* %						
**Active substance**	**Concentration**	**Solvent**	**Release**	**Day 1**	**Day 2**	**Day 5**	**Day 7**	**Day 9**	**Day 12**
Amoxicillin	10,000 µg/mL	H_2O	100 ± 2.1	82.9 ± 2.7	56.4 ± 1.3	28.7 ± 0.9	19.5 ± 0.7	11.1 ± 0.0	6.2 ± 0.5
	1000 µg/mL	TSB	100 ± 3.3	54.3 ± 5.5	27.7 ± 1.8	13.0 ± 0.2	11.1 ± 0.8	7.1 ± 0.3	4.8 ± 0.3
	100 µg/mL	TSB	100 ± 0.1	52.7 ± 1.0	24.0 ± 0.4	12.6 ± 0.2	10.3 ± 0.7	7.2 ± 0.3	4.7 ± 0.1
	10 µg/mL	TSB	100 ± 0.9	54.8 ± 0.8	23.8 ± 0.9	14.0 ± 0.5	10.6 ± 0.3	6.7 ± 0.3	5.1 ± 0.3
	1 µg/mL	TSB	100 ± 1.0	58.5 ± 0.2	18.1 ± 1.8	14.5 ± 1.8	11.5 ± 0.9	7.1 ± 0.4	5.9 ± 0.4
Cefotaxime	5000 µg/mL	H_2O	100 ± 0.2	66.5 ± 1.7	40.8 ± 1.2	12.6 ± 1.0	6.9 ± 0.5	3.3 ± 0.2	2.0 ± 0.1
	500 µg/mL	TSB	100 ± 1.9	70.3 ± 1.4	43.6 ± 0.6	12.7 ± 1.3	7.0 ± 0.0	3.3 ± 0.2	2.0 ± 0.0
	50 µg/mL	TSB	100 ± 1.0	70.2 ± 2.1	43.3 ± 1.5	12.4 ± 0.7	7.1 ± 0.1	3.1 ± 0.1	1.8 ± 0.1
	5 µg/mL	TSB	100 ± 2.0	72.6 ± 2.8	46.3 ± 0.9	13.1 ± 0.0	7.7 ± 0.4	3.3 ± 0.2	1.4 ± 0.0
	0.5 µg/mL	TSB	100 ± 7.8	77.5 ± 3.4	41.4 ± 2.0	14.0 ± 1.5	11.0 ± 0.3	9.4 ± 0.4	9.3 ± 0.6
Neomycin	40,000 µg/mL	H_2O	100 ± 0.4	89.3 ± 2.4	109.7 ± 0.5	101.9 ± 2.1	91.0 ± 4.1	99.4 ± 1.0	94.0 ± 0.3
	4000 µg/mL	TSB	100 ± 1.8	79.6 ± 0.2	89.0 ± 2.2	80.7 ± 1.8	63.5 ± 0.5	61.1 ± 1.3	52.7 ± 0.6
	400 µg/mL	TSB	100 ± 0.9	72.2 ± 5.1	72.3 ± 2.6	55.4 ± 2.5	43.9 ± 0.7	42.5 ± 1.3	31.2 ± 0.7
	40 µg/mL	TSB	100 ± 1.9	63.6 ± 0.4	61.8 ± 0.5	48.4 ± 3.2	33.2 ± 1.6	34.8 ± 0.3	21.2 ± 0.2
	4 µg/mL	TSB	100 ± 3.0	71.9 ± 11.5	22.2 ± 3.8	18.8 ± 0.4	22.5 ± 0.2	18.1 ± 1.5	11.7 ± 0.9
Oxytetracycline	5000 µg/mL	H_2O	100 ± 1.4	96.5 ± 3.7	74.8 ± 1.7	50.6 ± 0.8	42.9 ± 0.2	37.3 ± 0.6	37.2 ± 2.7
	500 µg/mL	TSB	100 ± 2.4	79.0 ± 3.2	50.9 ± 3.0	10.0 ± 0.6	4.4 ± 0.2	2.0 ± 0.0	2.0 ± 0.2
	50 µg/mL	TSB	100 ± 4.3	75.0 ± 1.0	45.8 ± 2.3	10.5 ± 0.2	4.6 ± 0.1	2.1 ± 0.1	1.7 ± 0.2
	5 µg/mL	TSB	100 ± 1.5	74.2 ± 1.5	45.0 ± 0.1	9.8 ± 0.4	4.0 ± 0.1	1.9 ± 0.1	1.7 ± 0.2
	0.5 µg/mL	TSB	100 ± 0.1	75.0 ± 2.7	44.6 ± 0.8	12.2 ± 0.3	5.2 ± 0.1	3.1 ± 0.0	2.7 ± 0.0
Florfenicol	40,000 µg/mL	H_2O	100 ± 0.9	99.8 ± 1.2	101.4 ± 0.6	101.5 ± 1.4	102.3 ± 3.3	103.9 ± 2.2	105.7 ± 2.7
	4000 µg/mL	TSB	100 ± 0.4	94.7 ± 5.2	93.3 ± 4.1	92.9 ± 1.2	95.4 ± 4.3	97.4 ± 1.6	97.4 ± 1.8
	400 µg/mL	TSB	100 ± 0.8	98.5 ± 2.5	101.4 ± 0.0	102.9 ± 1.2	102.3 ± 0.1	102.3 ± 4.4	103.7 ± 3.8
	40 µg/mL	TSB	100 ± 1.8	99.3 ± 3.7	101.8 ± 3.9	102.2 ± 5.8	104.0 ± 0.3	103.8 ± 2.1	103.0 ± 3.6
	4 µg/mL	TSB	100 ± 0.8	100.1 ± 0.4	101.9 ± 3.7	103.0 ± 1.7	102.8 ± 1.2	104.1 ± 1.7	102.2 ± 3.1

RSD: relative standard deviation; TSB: tryptone-soy broth

Supplementary Table S50 Stability of aqueous stock solutions and dilution series per active substance on each measurement day (continued)

			Mean % of initial concentration ± RSD %						
**Active substance**	**Concentration**	**Solvent**	**Release**	**Day 1**	**Day 2**	**Day 5**	**Day 7**	**Day 9**	**Day 12**
Enrofloxacin	150 µg/mL	H_2O	100 ± 0.6	101.0 ± 3.8	101.8 ± 3.8	89.6 ± 0.7	95.2 ± 1.2	91.6 ± 3.8	89.6 ± 2.8
	15 µg/mL	TSB	100 ± 10.3	95.9 ± 11.9	99.6 ± 9.2	94.6 ± 12.3	98.6 ± 9.6	93.2 ± 8.0	94.8 ± 6.2
	1.5 µg/mL	TSB	100 ± 1.7	98.1 ± 7.7	91.5 ± 8.9	82.3 ± 0.4	87.2 ± 1.5	81.1 ± 1.4	81.8 ± 0.1
	0.15 µg/mL	TSB	100 ± 2.1	96.6 ±1.6	94.1 ± 3.8	85.6 ± 3.7	86.2 ± 3.6	85.8 ± 2.9	86.4 ± 0.5
	0.015 µg/mL	TSB	100 ± 20.4	101.8 ± 5.8	100.1 ± 12.4	93.5 ± 10.5	92.4 ± 6.0	91.3 ± 9.2	91.7 ± 10.3
Colistin	2500 µg/mL	H_2O	100 ± 4.1	89.2 ± 5.7	73.5 ± 5.8	67.7 ± 2.5	62.9 ± 4.3	54.0 ± 7.0	40.5 ± 0.1
	250 µg/mL	TSB	100 ± 7.1	76.5 ± 5.4	57.9 ± 6.6	53.2 ± 0.9	48.9 ± 0.9	43.2 ± 0.1	34.3 ± 1.1
	25 µg/mL	TSB	100 ± 3.9	74.6 ± 2.6	53.4 ± 5.9	48.6 ± 1.7	37.1 ± 1.8	33.3 ± 3.1	24.9 ± 2.2
	2.5 µg/mL	TSB	100 ± 2.0	50.2 ± 2.9	41.1 ± 4.0	31.4 ± 3.2	28.5 ± 2.8	21.8 ± 0.2	15.7 ± 0.2
	0.25 µg/mL	TSB	100 ± 0.5	65.5 ± 3.5	44.3 ± 0.8	33.0 ± 0.9	30.3 ± 0.4	26.5 ± 0.5	21.3 ± 1.9
Sulfamethoxazole	19,047.5 µg/mL	H_2O	100 ± 1.2	102.2 ± 1.8	102.2 ± 1.3	101.0 ± 1.9	104.3 ± 1.2	104.6 ± 2.8	96.5 ± 6.1
	1905 µg/mL	TSB	100 ± 0.2	99.4 ± 0.3	102.0 ± 1.4	98.9 ± 4.2	97.5 ± 0.7	95.4 ± 0.3	91.2 ± 0.5
	190.5 µg/mL	TSB	100 ± 1.4	101.9 ± 1.6	100.5 ± 0.2	99.3 ± 2.0	98.9 ± 1.0	97.3 ± 0.0	94.4 ± 7.5
	19.1 µg/mL	TSB	100 ± 1.6	104.7 ± 0.1	101.3 ± 3.3	96.3 ± 0.5	92.9 ± 3.3	94.0 ± 0.7	92.2 ± 0.4
	1.9 µg/mL	TSB	100 ± 1.9	103.3 ± 0.2	105.4 ± 3.6	93.8 ± 1.5	90.4 ± 1.2	87.0 ± 1.9	86.6 ± 1.4
Trimethoprim	952.5 µg/mL	H_2O	100 ± 2.3	101.2 ± 0.4	100.1 ± 2.6	100.7 ± 0.1	99.3 ± 0.6	100.6 ± 1.6	98.8 ± 3.3
	95 µg/mL	TSB	100 ± 0.9	101.0 ± 1.0	103.5 ± 3.8	97.7 ± 4.0	96.9 ± 2.5	96.1 ± 0.3	93.2 ± 2.5
	9.5 µg/mL	TSB	100 ± 0.9	99.1 ± 1.4	99.5 ± 5.0	98.7 ± 0.3	98.0 ± 0.9	96.7 ± 1.6	92.8 ± 2.0
	0.95 µg/mL	TSB	100 ± 0.1	98.9 ± 1.7	101.0 ± 0.6	98.0 ± 1.9	94.9 ± 3.8	93.0 ± 1.7	90.1 ± 2.5
	0.095 µg/mL	TSB	100 ± 0.2	98.2 ± 0.8	100.8 ± 0.4	95.0 ± 1.8	90.1 ± 0.5	86.7 ± 1.2	85.0 ± 0.5

RSD: relative standard deviation; TSB: tryptone-soy broth
